# Self-assembled orthoester cryptands: orthoester scope, post-functionalization, kinetic locking and tunable degradation kinetics†
†Electronic supplementary information (ESI) available: Experimental procedures, characterization and spectral data, crystallographic data. CCDC 1823126, 1823728, 1823730, 1823774, 1823819, 1823845, 1824092 and 1832928. For ESI and crystallographic data in CIF or other electronic format see DOI: 10.1039/c8sc01750f


**DOI:** 10.1039/c8sc01750f

**Published:** 2018-04-27

**Authors:** Henrik Löw, Elena Mena-Osteritz, Max von Delius

**Affiliations:** a Institute of Organic Chemistry and Advanced Materials , University of Ulm , Albert-Einstein-Allee 11 , 89081 Ulm , Germany . Email: max.vondelius@uni-ulm.de

## Abstract

Dynamic adaptability and biodegradability are key features of functional, 21^st^ century host–guest systems. We have recently discovered a class of tripodal supramolecular hosts, in which two orthoesters act as constitutionally dynamic bridgeheads. Having previously demonstrated the adaptive nature of these hosts, we now report the synthesis and characterization – including eight solid state structures – of a diverse set of orthoester cages, which provides evidence for the broad scope of this new host class. With the same set of compounds, we demonstrated that the rates of orthoester exchange and hydrolysis can be tuned over a remarkably wide range, from rapid hydrolysis at pH 8 to nearly inert at pH 1, and that the Taft parameter of the orthoester substituent allows an adequate prediction of the reaction kinetics. Moreover, the synthesis of an alkyne-capped cryptand enabled the post-functionalization of orthoester cryptands by Sonogashira and CuAAC “click” reactions. The methylation of the resulting triazole furnished a cryptate that was kinetically inert towards orthoester exchange and hydrolysis at pH > 1, which is equivalent to the “turnoff” of constitutionally dynamic imines by means of reduction. These findings indicate that orthoester cages may be more broadly useful than anticipated, *e.g.* as drug delivery agents with precisely tunable biodegradability or, thanks to the kinetic locking strategy, as ion sensors.

## Abstract 2

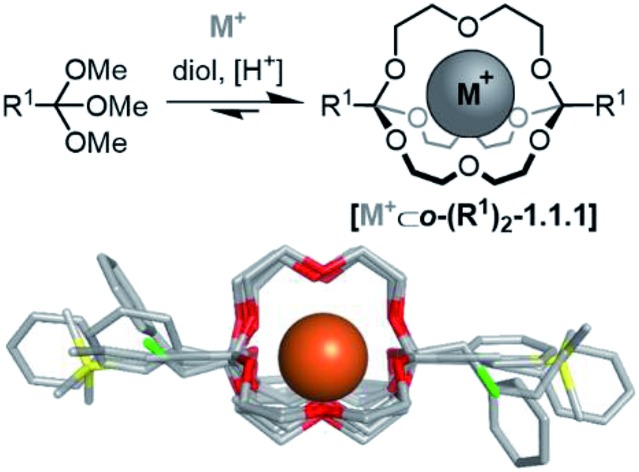
Self-assembled orthoester cryptands offer appealing properties for applications in ion sensing and transport, such as convenient post-functionalization and tunable biodegradation.

## Introduction

**Scheme 1:**
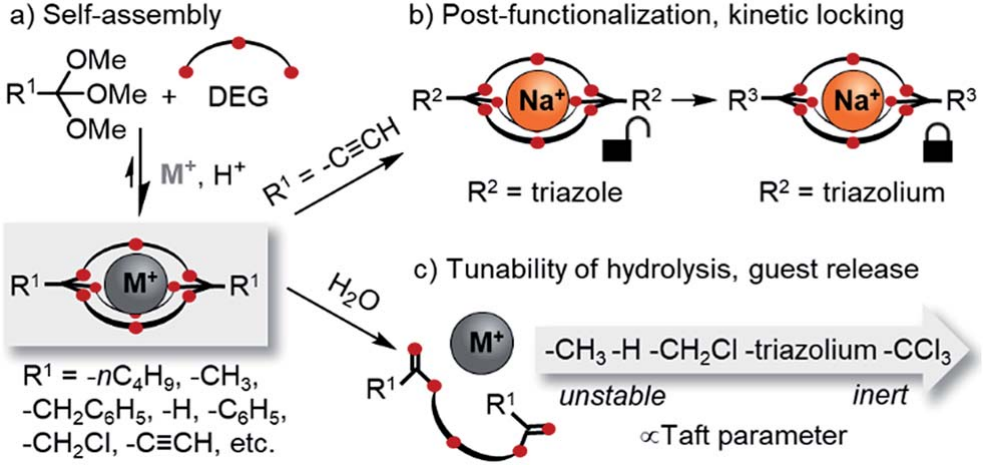
Overview on the scope of this study. (a) Template-directed self-assembly of orthoester cryptates: scope of orthoesters (R^1^) investigated in this contribution; (b) post-functionalization (R^2^) and kinetic locking (R^3^) of orthoester cryptates; (c) tunability of the degradation kinetics. DEG: diethylene glycol. M^+^ in this work: Na^+^ or Li^+^.

The development of new macro(bi)cyclic compounds has been a key driving force of progress in supramolecular chemistry.^1^ The discovery of pillararenes,^2^ for instance, has opened up new avenues in supramolecular polymer chemistry,^3^ and the rational design of cyanostar^4^ and triazolophane macrocycles^5^ enabled remarkable binding affinities for hard-to-bind anions such as PF_6_^–^, as well as unprecedented rotaxane syntheses4a,^6^ and fundamental insights on the nature of hydrogen bonding.^7^ In this article, we follow up on our discovery of a new class of self-assembled macrobicyclic host^8^ and report comprehensive data on the scope, structure and properties of these compounds (Scheme 1).

Dynamic combinatorial chemistry^9^ has been used extensively for generating macrocycles and cages from smaller subcomponents,9g,^10^ yet prior to our discovery of orthoester cryptands,^8^ there were only few reports on the self-assembly of purely organic dynamic covalent cages suitable to encapsulate single^11^ cationic^12^ or anionic^13^ guests. Self-assembled macrobicyclic hosts beyond dynamic covalent chemistry have been reported, most notably metallosupramolecular cryptates^14^ as well as clathrochelates.^15^ However, these compounds share with conventional cryptands the feature that their host framework is no longer dynamic and stimuli-responsive once the self-assembly process is complete. This is different for orthoester cryptands, which under anhydrous acidic conditions are adaptive to their environment,^16^ which allowed us to demonstrate that a wide range of metal ions selectively directs the self-assembly of their thermodynamically preferred host.^17^

Another interesting feature of orthoester cryptands is that, in contrast to conventional cryptands featuring nitrogen bridgeheads, their synthesis requires only a single step whose yield can be as high as 92%, as we demonstrate in this work (Scheme 1a). Moreover, orthoester bridgeheads possess a substituent (R^1^, Scheme 1) that should allow modulating the stability, solubility and lipophilicity of the cages and provide a convenient handle for adding further functionality. However, prior to this work, only three such substituents (R^1^ = –CH_3_, –CH_2_CH_3_, –H) have been explored.^8^

When considering applications in the emerging field of supramolecular medicinal chemistry,^18^ the most important feature of orthoester cryptands is their inherent tendency to hydrolyze^19^ under general acid catalysis to ring-opened esters, which leads to the irreversible release of the encapsulated guest. Although earlier studies have investigated the kinetics of orthoester hydrolysis and its pH dependency,^20^ a systematic study on a wide range of orthoester substituents (R^1^, Scheme 1) is still elusive, and the kinetics of orthoester exchange as a new addition to the toolbox of dynamic covalent chemistry^21^ remain unexplored.

Herein, we report a comprehensive study on the scope and generality of self-assembled orthoester cryptands (Scheme 1a) as well as their post-functionalization and post-synthetic stabilization against hydrolysis or exchange reactions (“kinetic locking”, Scheme 1b). Moreover, we explored the question to which extent the kinetics of orthoester exchange and hydrolysis can be tuned as a function of different orthoester substituents and whether the observed trends can be rationalized on the basis of linear free energy relationships (Scheme 1c).^22^

## Results and discussion

### Scope of orthoester cryptand self-assembly

**Scheme 2:**
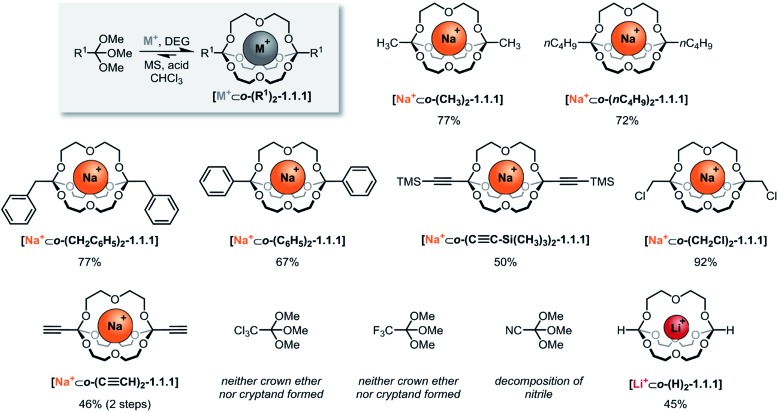
Scope of self-assembled orthoester cryptands. Reaction conditions: 60 μmol sodium tetrakis[3,5-bis(trifluoromethyl)phenyl]borate (BArF) or LiBArF, 180 μmol diethylene glycol, 120 μmol orthoester, 1.2 μmol TFA (R^1^ = –CH_2_Cl: 12 μmol TFA) in CHCl_3_, 7–13 days (for further details, see ESI†). Percent values indicate isolated yields. M^+^: Na^+^; Li^+^. DEG: diethylene glycol. MS: 5 Å molecular sieves.

The systematic exploration of the scope and generality of the synthesis of orthoester cryptands was the first objective of this study, because previous investigations had been limited to orthoformates (R^1^ = H), orthoacetates (R^1^ = CH_3_) and orthopropanoates (R^1^ = CH_2_CH_3_), and the self-assembly had only been successful for the two latter starting materials.^8^,^17^ We therefore chose five commercial and six non-commercial^23^ orthoesters with diverse functional groups ranging from electron-donating (*e.g.* R^1^ = –*n*C_4_H_9_) to aromatic (*e.g.* R^1^ = –C_6_H_6_) and strongly electron-withdrawing (*e.g.* R^1^ = –CF_3_). Furthermore, an alkynyl substituted orthoester was prepared in view of a possible post-functionalization of the self-assembled host. As shown in Scheme 2, these eleven orthoesters were subjected to standard reaction conditions for orthoester exchange. Specifically, these reaction conditions entail the use of diethylene glycol (DEG; grey box in Scheme 2) as a ligand for cation binding and substrate for orthoester/alcohol exchange, as well as trifluoroacetic acid (TFA) as catalyst. In addition, a suitable alkali metal template (*e.g.* NaBArF) and molecular sieves (5 Å) are required to provide a driving force for cryptate self-assembly and to maintain anhydrous reaction conditions.

By optimizing our procedures for preparing anhydrous starting materials (see ESI†), we were able to minimize the undesired hydrolysis of orthoesters to esters and to improve the isolated yield of previously reported orthoacetate cryptate **[Na^+^⊂*o*-(CH_3_)_2_-1.1.1]**^8^ from 67% to 77%. Yields in the range of 50% to 92% were obtained for five new orthoester cryptates (R^1^ = –*n*C_4_H_9_, –CH_2_C_6_H_5_, –C_6_H_5_, –C≡C–Si(CH_3_)_3_ and –CH_2_Cl; Scheme 2), demonstrating that alkyl, benzyl and phenyl substituted orthoester cryptates can be accessed in a straight-forward manner. The reaction times were relatively long (7 to 13 days), and we had previously speculated that this is mainly due to the slow uptake of methanol by molecular sieves, which presumably provides an additional entropic driving force for self-assembly.^24^

Our study on the kinetics of orthoester exchange supports this hypothesis, by showing that the self-assembly reactions require significantly more time than simpler exchange reactions of the same orthoester substrates (*vide infra*). Purification of the crude cryptates was possible by passing a chloroform solution through a short plug of silica gel. The highly efficient synthesis of a chloromethyl-substituted cage shows that mildly electron-withdrawing substituents are tolerated, even though their presence presumably destabilizes the oxonium ion intermediate of the exchange reaction. In fact, the observed isolated yield of 92% might well be a consequence of this effect, because, for the same reason, this cryptate is particularly stable against hydrolysis (*vide infra*), which minimizes any losses during purification.

The valuable alkynyl-substituted cryptate **[Na^+^⊂*o*-(C≡CH)_2_-1.1.1]** could be obtained in 46% yield over two steps after deprotection of the corresponding TMS-alkynyl-substituted cryptate. We were unable to self-assemble this compound directly from the corresponding orthoester (R^1^ = C≡CH), which is likely a result of the stronger electron-withdrawing effect of the free alkyne, in comparison to the silyl-protected analogue.‡
‡In principle, oxonium ions of alkynyl-substituted orthoesters could undergo 1,4-addition with alcohol nucleophiles, thus generating tetrasubstituted allene derivatives. However, our results suggest that such side reactions do not occur. For instance, the orthoester with R^1^ = –C≡CH undergoes clean exchange in the presence of 10% TFA (Table 2). In line with this reasoning, we did not observe any evidence for cryptand formation starting from even more electron-deficient orthoesters (R^1^ = –CCl_3_, –CF_3_, –CN). It should be noted that, in model experiments (*vide infra*), we were able to induce orthoester exchange in two of these substrates, but the required strong acid catalysts (*e.g.*, trifluoromethanesulfonic acid) appear to be too harsh to allow for template-directed cryptate self-assembly.

Arguably the most intriguing case in this scope study is the reaction of trimethyl orthoformate (R^1^ = H) with diethylene glycol. In our initial communication on cryptate **[Na^+^⊂*o*-(CH_3_)_2_-1.1.1]** we had reported that trimethyl orthoformate (H–C(OCH_3_)_3_) under standard conditions gives rise to the self-assembly of crown ether complexes **[Na^+^⊂*o*-(H)_2_-(OCH_3_)_2_-16-crown-6]**, but not to the corresponding cryptands. This was a surprising finding, because the cryptate self-assembly would simply require the addition of one additional DEG bridge to the crown ether complex. We have now discovered that an orthoformate cryptate **[Li^+^⊂*o*-(H)_2_-1.1.1]** can in fact be obtained, but only if a lithium, instead of a sodium template is used.

To better understand this unexpected result, we isolated empty cryptand ***o*-(H)_2_-1.1.1** by treatment of **[Li^+^⊂*o*-(H)_2_-1.1.1]** with anion exchange resin (Cl form; for further details, see ESI†) to study the thermodynamics of lithium and sodium binding. Therefore, we performed ^1^H NMR titrations with NaBArF and LiBArF in acetonitrile at different concentrations (1 to 10 mM) and fitted the resulting binding isotherms with a 1 : 1 stoichiometric model on the website ; supramolecular.org (original data is available for download in the spirit of “open-science”,^26^ see ESI†). The quality of the data was evaluated based on triplicate titrations (95% confidence interval, Table 1) and statistical analyses of the goodness of fit (see ESI†). The resulting association constants show that lithium binds to ***o*-(H)_2_-1.1.1** about 200 times more strongly than sodium (see Table 1). A comparison with data on methyl-substituted cryptand ***o*-(CH_3_)_2_-1.1.1** (ref. 25) (Table 1, right column) reveals that the substitution of methyl for hydrogen in ***o*-(H)_2_-1.1.1** leads to stronger binding of lithium and weaker binding of sodium. These *K*_a_ values provide a compelling explanation for our unexpected observation that cryptand ***o*-(H)_2_-1.1.1** can be obtained with a lithium template, but not with a sodium template, even though titrations were performed in acetonitrile and self-assembly reactions are carried out in chloroform,§
§NMR titrations had to be carried out in acetonitrile to guarantee fast exchange on the NMR timescale, as well as sufficient solubility of all starting materials. where the relative magnitudes of ion–dipole interactions are typically more pronounced than in acetonitrile. It will be interesting to address the question why cryptand ***o*-(H)_2_-1.1.1** exhibits such outlier thermodynamic behavior, but this will likely require high-level computational studies that are beyond the scope of this contribution.

**Table 1:** Anomalous Li^+^/Na^+^ affinity of orthoformate cryptand ***o*-(H)_2_-1.1.1** (*K*_a_ values)^*a*^

Entry		***o*-(H)_2_-1.1.1**	***o*-(CH_3_)_2_-1.1.1** (ref. 25)
1	Li^+^	13 000 ± 1000 M^–1^	1700 M^–1^
2	Na^+^	60 ± 20 M^–1^	1300 M^–1^

^*a*^Titrations were performed using BArF salts in acetonitrile at different host concentrations (1 to 10 mM). Association constants were determined by ^1^H NMR titrations and fitting of isotherms on supramolecular.org. Estimates of uncertainty reflect 95% confidence intervals (triplicate titrations). For further details, see ESI.

### Solid state structures

**Chart 1:**
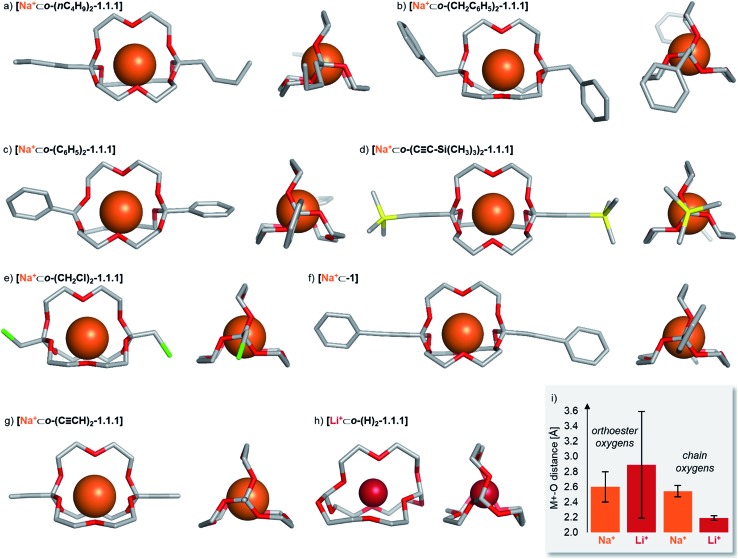
Solid-state structures of eight orthoester cryptands. Single crystals were obtained by the layering method (hexane/chloroform). Hydrogen atoms, anions, solvent and disorder (where applicable) are omitted for clarity. Metal ions are displayed at 100% of effective ionic radius.^27^ (a) Crystal system: triclinic. Na–O distance (orthoester oxygen): 2.45–2.92 Å. Na–O distance (chain oxygen): 2.48–2.53 Å. (b) Crystal system: monoclinic. Na–O distance (orthoester oxygen): 2.47–2.76 Å. Na–O distance (chain oxygen): 2.51–2.57 Å. (c) Crystal system: orthorhombic. Na–O distance (orthoester oxygen): 2.32–3.53 Å. Na–O distance (chain oxygen): 2.37–2.46 Å. (d) Crystal system: triclinic. Na–O distance (orthoester oxygen): 2.48–2.59 Å. Na–O distance (chain oxygen): 2.54–2.67 Å. (e) Crystal system: monoclinic. Na–O distance (orthoester oxygen): 2.47–2.89 Å. Na–O distance (chain oxygen): 2.48–2.57 Å. (f) Crystal system: triclinic. Na–O distance (orthoester oxygen): 2.43–2.69 Å. Na–O distance (chain oxygen): 2.56–2.61 Å. (g) Crystal system: triclinic. Na–O distance (orthoester oxygen): 2.46–2.66 Å. Na–O distance (chain oxygen): 2.53–2.67 Å. (h) Crystal system: orthorhombic. Li–O distance (orthoester oxygen): 1.96–3.67 Å. Li–O distance (chain oxygen): 2.16–2.23 Å. (i) Comparison of average Na–O distance of all sodium-based solid-state structures with average Li–O distance in **[Li^+^⊂*o*-(H)_2_-1.1.1]**, including standard deviation. For further details, see ESI.†

To gain structural insights into the prepared macrobicyclic compounds, we attempted to crystallize all new orthoester cryptates by slow diffusion of hexane into chloroform solutions. To our delight, we were able to obtain single crystals suitable for X-ray crystallography of eight new orthoester cryptates (see Chart 1). All sodium-based architectures, including previously reported **[Na^+^⊂*o*-(CH_3_)_2_-1.1.1]**,^8^ feature the encapsulation of the alkali metal ion at the centre of the cavity, held in place by nine Na–O bonds. The only exception to this mode of binding is **[Na^+^⊂*o*-(C_6_H_5_)_2_-1.1.1]**, in which only eight Na–O bonds are observed. One orthoester oxygen is more distant (3.53 Å) and two are closer to the metal ion (2.32, 2.40 Å) than in all other sodium-containing structures (2.43–2.92 Å). Due to this slight distortion, caused most likely by packing effects, the three chain oxygen atoms are closer to the metal ion (2.37–2.46 Å) than in the other seven sodium cryptates (2.48–2.67 Å; see Chart 1i for a statistical analysis of bond lengths). Most of the orthoester cryptates crystallize in a triclinic crystal system (space group *P*11̄), but monoclinic (), but monoclinic (*P*2_1_/*n* or *P*2_1_/*c*) and orthorhombic (*Pbca*) systems were also observed.

We were also able to obtain X-ray crystallographic data on a cryptate encapsulating a lithium ion for the first time. Numerous previous attempts at crystallizing **[Li^+^⊂*o*-(CH_3_)_2_-1.1.1]** had been met with failure, presumably due to the relatively low binding constant (Table 1) and (degenerate) binding dynamics in this host.^25^ In stark contrast to the sodium-based structures, cryptate **[Li^+^⊂*o*-(H)_2_-1.1.1]** features only five Li–O bonds. This binding mode leads to rather short Li–O bonds of the three chain oxygen atoms (2.16–2.23 Å) and even shorter contacts for the two orthoester oxygen atoms that are involved in Li–O bonds (1.96 Å). Hence, the remaining four orthoester oxygen atoms are significantly more distant (2.94–3.67 Å) than in the sodium-containing solid state structures (see Chart 1i). The crystal system observed for **[Li^+^⊂*o*-(H)_2_-1.1.1]** is orthorhombic (*P*2_1_2_1_2_1_).

### Kinetics of orthoester exchange and hydrolysis

To shed light on the differences in reactivity and stability of orthoesters and their corresponding cryptands, we decided to investigate the kinetics of the exchange reactions of a diverse set of orthoesters with ethanol (see Table 2). To this end, 13 trimethyl orthoesters (**A_3_**) were treated with three equivalents of ethanol (**B**) and acid catalyst under standardized conditions and the reaction progress was monitored for up to 16 hours by ^1^H NMR spectroscopy. To account for the vast differences in reactivity between the orthoesters, both the amount of acid, as well as the type of acid had to be varied. For example, the CCl_3_– and CF_3_–substituted orthoesters were found to be so inert that even large quantities of trifluoroacetic acid did not lead to discernible exchange products. Hence, whenever an orthoester was found to be too inert to react within 16 h under mild reaction conditions, the next harsher set of reaction conditions was investigated (and so on). Besides this step-wise enhancement of acid catalysis, the second crucial parameter in Table 2 is the equilibration time *t*, which we defined as the first data point at which ≥99% conversion to the equilibrium distribution was reached based on ^1^H NMR integration. The kinetic data allowed us to order the 13 studied orthoesters from most reactive to most inert (top to bottom in Table 2).

**Table 2:** Kinetics and scope of orthoester exchange^*a*^

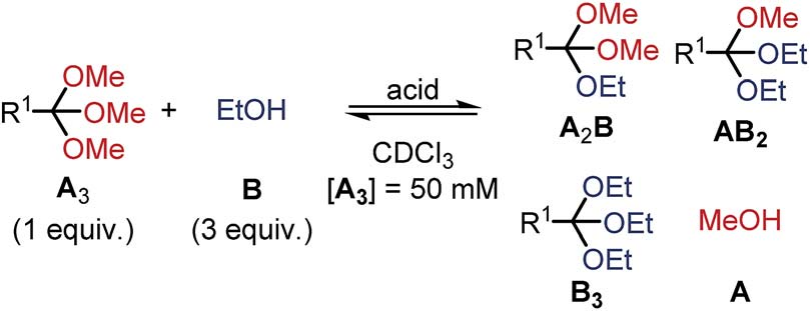				
Acid	R^1^	*t* ^*b*^ [min]	Product ratio (**A_3_** : **A_2_B** : **AB_2_** : **B_3_**)	Taft^22^
0.01% TFA	–*n*C_4_H_9_	10	n.d.	–0.13
0.01% TFA	–CH_3_	280	26 : 44 : 24 : 6	0.00
0.1% TFA	–CH_2_C_6_H_5_	650	23 : 41 : 29 : 6	0.22
1% TFA	–H	40	19 : 43 : 31 : 7	0.49
1% TFA	–C_6_H_5_	50	23 : 42 : 27 : 7	0.60
1% TFA	–C≡C–TMS	90	22 : 41 : 29 : 7	—
1% TFA	–Triazole^*c*^	130	n.d.	—
1% TFA	–CH_2_Cl	640	16 : 42 : 32 : 10	1.05
10% TFA	–C≡CH	300	32 : 35 : 26 : 6	2.18
50% TfOH	–CCl_3_	330	n.d.	2.65
50% TfOH	–Triazolium^*d*^	490	16 : 39 : 33 : 11	—
100% TfOH	–CF_3_	>1000	n.d.	2.61
100% TfOH	–CN	Decomposition		3.30

^*a*^Reaction conditions: orthoester (**A_3_**, 37.5 μmol, 1.0 equiv.), alcohol (**B**, 112.5 μmol, 3.0 equiv.), internal standard (9.41 μmol) and acid catalyst (0.01 to 100 mol%) were added to the reaction vessel from stock solutions. CDCl_3_ was added to obtain a total volume of 750 μL. The reaction was monitored by ^1^H NMR spectroscopy.

^*b*^ *t*: equilibration time, defined as data point when 99% conversion to plateau-level of methanol **A** was exceeded for the first time; n.d.: not determined due to peak overlap in NMR spectrum. Estimated error: ±5%.

^*c*^1-Benzyl-4-(trimethoxymethyl)-1*H*-1,2,3-triazole.

^*d*^1-Benzyl-3-methyl-4-(trimethoxymethyl)-1*H*-1,2,3-triazol-3-ium.

A comparison of the equilibration times reveals that electron-rich orthoesters are vastly more reactive than electron-deficient orthoesters and that the reactivity of orthoesters can be fine-tuned over a remarkably wide range. For instance, trimethyl orthovalerate (R^1^ = –*n*C_4_H_9_, see Table 2) equilibrates with ethanol in only 13 min with 0.01% TFA, whereas the reaction of 1,1,1-trifluoro-2,2,2-trimethoxyethane (R^1^ = –CF_3_, see Table 2) did not reach equilibrium after 16 h, even with 100 mol% trifluoromethanesulfonic acid (TfOH).

Because the results summarized in Table 2 represent the first account of a reactivity sequence for orthoester exchange, we attempted to identify a linear free energy relationship (LFER) that would correlate with the observed trend. We quickly noticed that the Hammett substituent parameter (*σ*) and variations thereof are a poor fit, which is not too surprising given that the Hammett reference reaction is rather unlike orthoester exchange. As can be deduced from Table 2, the Taft parameter for the R^1^ group does, however, correlate very well with the observed reactivity trend with the only exception (CCl_3_*vs.* CF_3_, see Table 2) being based on a very narrow margin and not relevant to templated cryptate self-assembly. From a mechanistic perspective, this finding makes good sense, because the Taft LFER is based on the acid-catalysed hydrolysis of aliphatic esters, which proceeds *via* the same oxonium intermediates as orthoester exchange.^28^ Because many orthoester derivatives are commercially available, this correlation with the Taft LFER will be very valuable for designing and optimizing future self-assemblies based on orthoester exchange.

In respect to the scope of self-assembled orthoester cryptates (*vide supra*), these results imply that there is a threshold where self-assembly is no longer possible, because the R^1^ substituent is too strongly electron-withdrawing and that this threshold lies between –CH_2_Cl (Taft 1.05, see Table 2) and –C≡CH (Taft 2.18, see Table 2). Nevertheless, we would like to emphasize that cryptands equipped with functional groups with Taft values > 1 are accessible *via* post-functionalization methods (*e.g.* R^1^ = –C≡CH by deprotection or –triazolium by methylation, *vide infra*).

When considering the thermodynamic, rather than kinetic implications of these results, it is evident from Table 2 that the observed equilibrium compositions (**A_3_** : **A_2_B** : **AB_2_** : **B_3_**) are mostly unaffected by the R^1^ substituent and the acid catalyst. Yet to our surprise, when plotting the evolution of individual species over time, we found that the thermodynamically least favoured product (**B_3_**) generally reaches its plateau level first. This finding can be rationalized by conflicting steric and electronic effects: the sterically more demanding ethyl group makes **B_3_** the thermodynamically least stable product, yet the oxonium ion that leads to the formation of **B_3_** is the most stable intermediate, which means that the conversion of **A_2_B** into **AB_2_** is slower than the conversion of **AB_2_** into **B_3_**. See ESI† for further details and kinetic simulations of this interesting “bottleneck” in the evolution of the chemical network over time,^29^ including the exchange reaction with 2-chloroethanol, which confirms the above reasoning.

In view of possible applications of orthoester cryptates in drug delivery,^19^,^30^ we turned our attention towards the hydrolytic degradation of orthoesters and the corresponding self-assembled cryptands (Table 3). Six representative orthoesters were dissolved in phosphate buffer solutions of varying pH. The reactions were monitored by ^1^H NMR spectroscopy to determine the half-life *t*_1/2_ and the kinetic rate *k*_obs_ of the hydrolysis reaction under pseudo first order conditions. As expected, the kinetics of orthoester hydrolysis follow the same trend that was observed for orthoester exchange. While electron-rich orthoesters (*e.g.*, R^1^ = –CH_3_, see Table 3, entry 1) hydrolyze readily even at neutral pH, more electron-deficient orthoesters (*e.g.*, R^1^ = –triazolium, –CCl_3_, see Table 3, entries 5 and 6) are remarkably stable: even at pH 1 their half-lives are beyond 16 h. In the context of drug delivery, we believe that orthoformates (R^1^ = –H; *t*_1/2_ 70 min at pH 6, see Table 3, entry 2) and chloromethyl-substituted orthoesters (R^1^ = –CH_2_Cl; *t*_1/2_ 121 min at pH 5 and 34 min at pH 4, see Table 3, entry 3) offer the most appealing hydrolysis profiles.¶
¶The toxicity profile of all possible hydrolysis products will need to be considered for applications in drug delivery. For instance, DEG will likely have to be replaced with a less toxic derivative (proof-of-principle reported in ref. 17) and R^1^ = CH_2_Cl might pose a major liability due to the potential formation of toxic chloroacetic acid.


**Table 3:** Hydrolysis of orthoesters and a representative orthoester cryptand^*a*^

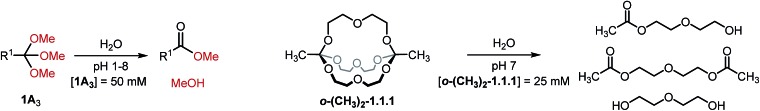									
Entry	R^1^		pH 8	pH 7	pH 6	pH 5	pH 4	pH 3	pH 1
1	–CH_3_	*t*_1/2_ [min], *k*_obs_ [s^–1^]	60, 1.8 × 10^–4^	10, 1.3 × 10^–3^	2, 6.1 × 10^–3^	<1			
2	–H	*t*_1/2_ [min], *k*_obs_ [s^–1^]	>1000	510, 2.5 × 10^–5^	70, 1.6 × 10^–4^	20, 8.8 × 10^–4^	7, 2.0 × 10^–3^	<1	
3	–CH_2_Cl	*t*_1/2_ [min], *k*_obs_ [s^–1^]		>1000	660, 1.7 × 10^–5^	120, 8.6 × 10^–5^	30, 2.9 × 10^–4^	<1	
4	–Triazole^*b*^	*t*_1/2_ [min]							<1^*c*^
5	–Triazolium^*d*^	*t*_1/2_ [min], *k*_obs_ [s^–1^]						Inert	>10 000, 1.6 × 10^–6^
6	–CCl_3_	*t*_1/2_ [min]							>1000^*c*^
7	***o*-(CH_3_)_2_-1.1.1**	*t*_1/2_ [min], *k*_obs_ [s^–1^]		30, 3.5 × 10^–4^					

^*a*^Reaction conditions: orthoester (**A_3_**, 37.5 μmol) or cryptand (***o*-(CH_3_)_2_-1.1.1**, 18.75 μmol) and internal standard (12.3 μmol) were added to the reaction vessel. Buffer solution was added to obtain a total volume of 750 μL. The reaction was monitored by ^1^H NMR spectroscopy. *t*_1/2_: half-life of starting material, defined as point when 50% of starting material were consumed. Estimated error: ±4%.

^*b*^1-Benzyl-4-(trimethoxymethyl)-1*H*-1,2,3-triazole.

^*c*^400 μL DMSO added to increase solubility of starting material. Comparison of measurements in pure buffer and with addition of DMSO revealed that *k*_obs_ is decreased by *ca.* one order of magnitude upon addition of the co-solvent (for further details, see ESI).

^*d*^1-Benzyl-3-methyl-4-(trimethoxymethyl)-1*H*-1,2,3-triazol-3-ium.

Finally, we monitored the hydrolysis of cryptand ***o*-(CH_3_)_2_-1.1.1** under comparable reaction conditions (see Table 3, entry 7). As anticipated,^8^ the observed hydrolysis rate for cryptand ***o*-(CH_3_)_2_-1.1.1** is somewhat lower than that for the simple orthoester but in the same range, indicating that our kinetic findings are transferable from orthoesters to orthoester cryptands.

### Post-functionalization and kinetic locking of orthoester cryptands

**Scheme 3:**
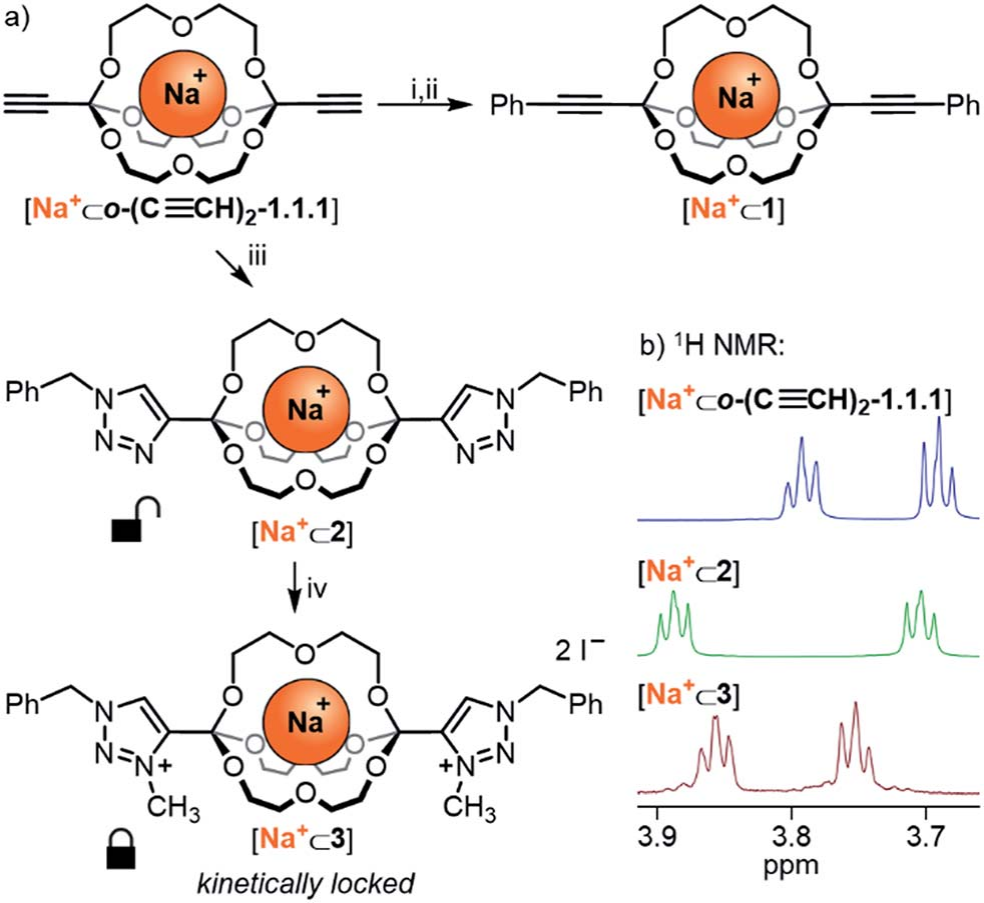
Post-functionalization of orthoester cryptands. (a) Reaction conditions: (i) **[Na^+^⊂*o*-(C≡CH)_2_-1.1.1]** (11.8 μmol, 1.0 equiv.), iodobenzene (23.6 μmol, 2.0 equiv.), NEt_3_ (35.4 μmol, 3.0 equiv.), CuI (0.12 μmol, 0.01 equiv.), Pd(PPh_3_)_4_ (0.12 μmol, 0.01 equiv.), THF, 40 °C, 4 d, 94%. (ii) **1** (9.28 μmol, 1.0 equiv.), NaBArF (9.28 μmol, 1.0 equiv.), CH_3_CN, r.t., 5 min, quant. (iii) **[Na^+^⊂*o*-(C≡CH)_2_-1.1.1]** (11.0 μmol, 1.0 equiv.), benzyl azide (22.0 μmol, 2.0 equiv.), Cu(MeCN)_4_PF_6_ (0.11 μmol, 0.01 equiv.), TBTA (0.11 μmol, 0.01 equiv.), MeOH, 70 °C, 24 h, 92%. (iv) **[Na^+^⊂2]** (3.9 μmol, 1.0 equiv.), MeI (excess), MeCN, 70 °C, 3 d, 83%. (b) Partial ^1^H NMR stacked plot (500 MHz, 298 K, CD_3_CN).

Having established the broad scope and the kinetic limitations of the self-assembly reaction, we turned our attention towards further diversifying orthoester cryptates by post-functionalization (see Scheme 3a).^31^ Cryptate **[Na^+^⊂*o*-(C≡CH)_2_-1.1.1]**, containing two functional alkyne handles, was subjected to a Sonogashira reaction with iodobenzene to furnish cryptand **1** in 94% yield. The basicity of the reaction conditions had the advantageous effect that no orthoester hydrolysis was observed, but also led to decomplexation, thus requiring a trivial second step to reintroduce the sodium guest, thus furnishing cryptate **[Na^+^⊂1]**. We believe that this type of reaction will be particularly useful for the preparation of intriguing degradable 1D polymers and for applications in ion sensing, because the aromatic substituents are in conjugation with the orthoester bridgeheads. The introduction of conjugated chromophores or fluorophores should result in pronounced differences in optical output depending on the nature of guest ions, and could also provide a theranostic readout^32^ for cage degradation and concomitant guest release.

An alternative method for post-functionalization was investigated by the CuAAC “click” reaction between **[Na^+^⊂*o*-(C≡CH)_2_-1.1.1]** and benzyl azide. This reaction proceeded in protocol, the product cryptate **[Na^+^⊂2]** was obtained in excellent yield (92%), and in contrast to the Sonogashira reaction without decomplexation of the metal guest. Although electronic communication between substituents and cage is likely weak in such bis-triazoles,^33^ we believe that this type of functionalization will prove valuable for installing functional moieties, for example for modulating lipophilicity, targeting biological binding motifs^34^ or stimuli-responsive triggering of cage decomposition.^35^

Encouraged by the efficient post-functionalization reactions and the observed differences in hydrolysis rates (Table 3), we wondered whether we could drastically stabilize compound **[Na^+^⊂2]** by methylation of the triazoles.^36^ At least in principle, the electron-withdrawing effect of the resulting triazolium on the orthoester bridgehead could lead to significantly reduced orthoester reactivity and pave the way towards applications of orthoester cryptands that require stability in water. As shown in Scheme 3a, treatment of **[Na^+^⊂2]** with an excess of iodomethane furnished the corresponding bis-triazolium cryptate **[Na^+^⊂3]** in 83% yield (Scheme 3a, bottom). In proof-of-principle experiments, the corresponding triazolium-substituted orthoester indeed proved to be inert towards hydrolysis at pH 3 (Table 3, entry 5) and underwent orthoester exchange only with 50% triflic acid (Table 2). At pH 1 the half-life for hydrolysis was beyond 16 h, whereas all other orthoesters suitable to prepare cryptates hydrolyzed rapidly under these conditions. The extrapolated half-life of the triazolium–orthoester in fact exceeded 10 000 min, whereas the half-life of the triazole–orthoester is below one minute, which accounts for an increase in stability of at least four orders of magnitude (Table 3, entries 4 and 5). As such, this post-stabilization method is akin to the kinetic locking of imines by means of reduction to the corresponding amines, which has found widespread use in the synthesis of mechanically interlocked molecules, organic cages, porous materials and other applications of dynamic covalent chemistry.^37^

## Conclusions

In this work, we have demonstrated that orthoester cryptands equipped with a broad range of substituents are accessible in one step *via* efficient, metal-templated self-assembly reactions. Cryptands substituted with strongly electron-withdrawing groups could not be obtained, but this limitation was circumvented by highly efficient post-functionalization methodologies based on a Sonogashira or CuAAC reaction of the bis-alkynyl cryptate. Methylation of a triazole-substituted cryptate furnished a supramolecular host that can be considered kinetically locked in respect to orthoester exchange and hydrolysis, but still binds effectively to cationic guests. In future studies, it could be beneficial to reverse this “locking” by site-selective *N*-dealkylation, which appears to be possible under relatively mild basic conditions.^38^ We also carried out detailed, comparative kinetic studies on orthoester exchange and hydrolysis, firmly establishing that the tunability of orthoester degradation makes these architectures interesting candidates for the (topical) delivery of ionic drugs, which strictly requires the biodegradability of otherwise toxic supramolecular hosts.^39^ Further studies on (an)ion encapsulation, sensing, transport and release are underway in our laboratory.

## Conflicts of interest

There are no conflicts to declare.

## Supplementary Material

Supplementary information

Crystal structure data
