# Supplementary material for: Self-assembled orthoester cryptands: orthoester scope, post-functionalization, kinetic locking and tunable degradation kinetics
Source: Chem Sci. 2018 Apr 27;9(21):4785–93. doi: 10.1039/c8sc01750f (PMC5982201; doi:10.1039/c8sc01750f)
Supplement: Supplementary file 1 [file SC-009-C8SC01750F-s001.pdf]

Electronic Supplementary Material (ESI) for Chemical Science.
This journal is © The Royal Society of Chemistry 2018

Supporting Information

Self-Assembled Orthoester Cryptands: Orthoester Scope, Post-Functionalization and Tunable Degradation Kinetics

Henrik Löw, Elena Mena-Osteritz and Max von Delius

Institute of Organic Chemistry and Advanced Materials, University of Ulm, Albert-Einstein-Allee 11, 89081 Ulm (Germany)

Email: max.vondelius@uni-ulm.de

Contents

1. General Experimental Section **S3**

2. Synthetic Procedures and Characterization Data **S5**

2.1 Syntheses of Orthoesters S5

Synthesis of (2,2,2-trimethoxyethyl)benzene S5

Synthesis of 3,3,3-trimethoxyprop-1-yne S6

Synthesis of 1-benzyl-4-(trimethoxymethyl)-1*H*-1,2,3-triazole S7

Synthesis of 1-benzyl-3-methyl-4-(trimethoxymethyl)-1H-1,2,3-triazol-3-ium methyl sulfate S9

2.2 Synthesis of Orthoester Cryptates S11

Optimized Synthesis of $[Na^+\subset o\text{-}(CH_3)_2\text{-}1.1.1]BArF^-$ S11

Synthesis of $o\text{-}(CH_3)_2\text{-}1.1.1$ S11

Synthesis of $[Na^+\subset o\text{-}(nC_4H_9)_2\text{-}1.1.1]BArF^-$ S11

Synthesis of $[Na^+\subset o\text{-}(CH_2C_6H_5)_2\text{-}1.1.1]BArF^-$ S13

Synthesis of $[Na^+\subset o\text{-}(C_6H_5)_2\text{-}1.1.1]BArF^-$ S14

Synthesis of $[Na^+\subset o\text{-}(C{\equiv}C\text{-}Si(CH_3)_3)_2\text{-}1.1.1]BArF^-$ S16

Synthesis of $[Na^+\subset o\text{-}(CH_2Cl)_2\text{-}1.1.1]BArF^-$ S17

Synthesis of $[Na^+\subset o\text{-}(C{\equiv}CH)_2\text{-}1.1.1]BArF^-$ S19

Synthesis of $[Li^+\subset o\text{-}(H)_2\text{-}1.1.1]BArF^-$ S20

Synthesis of $o\text{-}(H)_2\text{-}1.1.1$ S22

2.3 Post-Functionalization S24

Sonogashira Reaction S24

CuAAC “Click” Reaction S27

Triazole Methylation S29

3. Experimental Binding Studies **S31**

4. Monitoring of orthoester exchange and hydrolysis S35
Exchange reactions with ethanol S35
Product distribution plots S44
Influence of R^3 on equilibration time and product distribution S46
Simulation of product distributions S49
Hydrolysis rates S52
5. Crystallographic Data S61
Compound [Na^+⊂*o*-(*n*C_4H_9)$_2$-1.1.1]$BArF^-$ S61
Compound [Na^+⊂*o*-($CH_2C_6H_5$)$_2$-1.1.1]$BArF^-$ S62
Compound [Na^+⊂*o*-(C_6H_5)$_2$-1.1.1]$BArF^-$ S63
Compound [Na^+⊂*o*-(C≡C-Si(CH_3)$_3$)$_2$-1.1.1]$BArF^-$ S64
Compound [Na^+⊂*o*-(CH_2Cl)$_2$-1.1.1]$BArF^-$ S65
Compound [Na^+⊂*o*-(C≡CH)$_2$-1.1.1]$BArF^-$ S66
Compound [Na^+⊂1]$BArF^-$ S67
Compound [Li^+⊂*o*-(H)$_2$-1.1.1]$BArF^-$ S68
Comparison of M^+-O distances and O-C-O angles S69
6. References S71

1. General Experimental Section

Reagents and instruments

All commercially available reagents were purchased from Sigma Aldrich, Alfa Aesar, Acros Organics or TCI and were used without further purification. Molecular sieves and aluminum oxide were dried for 3 days at 150 °C under reduced pressure (10^{-2} mbar) before use. $CDCl_3$ was dried for at least 3 days over molecular sieves. All solvents were dried over molecular sieves for at least 24 hours. All orthoester exchange reactions (catalyzed by TFA) were carried out under nitrogen. NMR spectra were recorded on Bruker Avance 400 (^{1}H: 400 MHz) spectrometers at 298 K and referenced to the residual solvent peak (^{1}H: $CDCl_3$, 7.26 ppm; ^{13}C: $CDCl_3$, 77.0 ppm). Coupling constants (J) are denoted in Hz and chemical shifts (δ) in ppm. Mass spectra were obtained on a Bruker SolariX (HRMS-ESI$^+$, Solvent: methanol or acetonitrile) instrument.

1,1,1-trichloro-2,2,2-trimethoxyethane[1], 1,1,1-trifluoro-2,2,2-trimethoxyethane[2], 2,2,2-trimethoxy-acetonitrile[3], trimethyl(3,3,3-trimethoxyprop-1-yn-1-yl)silane[4] and Lithium tetrakis[3,5-bis(trifluoromethyl)phenyl]borate[5] were synthesized according to literature procedures.

Preparation of stock solutions

To achieve a high level of stoichiometric accuracy, all substrates and reagents in the orthoester exchange reactions were added from stock solutions. To obtain the **stock solution** for **General Procedure B**, **Orthoester** (375 µmol, 1.0 equiv.) and **alcohol** (1.125 mmol, 3.0 equiv.) were combined and $CDCl_3$ was added to obtain a total volume of 1.0 mL. To obtain the **stock solution of internal standard**, 50 µL toluene were topped up with $CDCl_3$ to obtain a total volume of 5 mL. To obtain the **acid stock solutions**, trifluoroacetic acid (375 µmol, 29.0 µL) was topped up with $CDCl_3$ to obtain a total volume of 1 mL. To obtain stock solutions for 1% to 0.01% acid catalysis this stock solution was diluted further. Triflic acid (375 µmol, 33.5 µL) was topped up with $CDCl_3$ to obtain a total volume of 1 mL. To obtain stock solutions for 1% acid catalysis this stock solution was diluted further. To obtain the **phosphate buffer stock solutions for General Procedure C**, phosphoric acid (100 mmol, 98.0 mg), potassium phosphate monobasic (100 mmol, 137.5 mg) and potassium phosphate dibasic (100 mmol, 177.1 mg) were dissolved in deuterium oxide (10 mL each). The phosphate stock solutions were mixed to obtain **buffer stock solutions** (pH 1-8). To obtain the **stock solution of internal standard for General Procedure C**, 50 µL THF were topped with deuterium oxide to obtain a total volume of 5 mL.

General Procedures

General Procedure A (cryptate syntheses):

Drying of starting materials: Sodium tetrakis[3,5-bis(trifluoromethyl)phenyl]borate or Lithium tetrakis[3,5-bis(trifluoromethyl)phenyl]borate was dissolved in anhydrous acetonitrile, trimethyl orthoacetate (1 mL per gram NaBArF) and a catalytic amount of TFA were added. The solvent was removed under reduced pressure, the salt was dried under high vacuum at 100 °C. Diethylene glycol and orthoesters were dried and stored over 3 Å MS and aluminum oxide.

Sodium tetrakis[3,5-bis(trifluoromethyl)phenyl]borate (60 µmol, 1.0 equiv., 54 mg) or Lithium tetrakis[3,5-bis(trifluoromethyl)phenyl]borate (60 µmol, 1.0 equiv., 52 mg), diethylene glycol (180 µmol, 3.0 equiv, 17.3 µL) and orthoester (120 µmol, 2.0 equiv) were dissolved in anhydrous chloroform (6 mL) under inert atmosphere. TFA stock solution (1.2-12 µmol, 0.01-0.1 equiv, 10-100 µL) was added. After equilibration of the reaction mixture, 5 Å MS was added. The reaction progress was monitored by ^{1}H-NMR spectroscopy. Upon completion, the reaction was quenched by addition of triethylamine and the solvent was removed under reduced pressure. The crude product was purified by passing it over a short plug of silica gel. The

plug was rinsed with anhydrous chloroform and removal of the solvent under reduced pressure gave the corresponding salts as colourless solids.

General Procedure B (NMR monitoring of orthoester exchange):

In an NMR tube with screw cap, Orthoester (**A**$_3$, 37.5 µmol, 1.0 equiv.), alcohol (**B**, 112.5 µmol, 3.0 equiv.), internal standard (9.41 µmol) and acid catalyst (0.01 to 100 mol%) were combined (from stock solutions, see above) and $CDCl_3$ was added to obtain a total volume of 750 µL. The NMR tube was inserted into an NMR spectrometer and the reaction progress was monitored every 1 to 10 minutes by ^{1}H NMR spectroscopy (temperature: 298 K). The ratio of R^3OH versus OR^3 was plotted over time until the development of R^3OH reached the plateau.

General Procedure C (NMR monitoring of orthoester hydrolysis):

In a standard NMR tube, internal standard (100 µL) and phosphate buffer (645 µL) were combined (from stock solutions, see above). Orthoester (**A**$_3$, 37.5 µmol) was added, the mixture was shaken and then inserted into an NMR spectrometer. The reaction was monitored every 1 to 5 minutes by ^{1}H NMR spectroscopy (temperature: 298 K). The ratio of hydrolysis product versus starting material was observed over time.

General Procedure D (NMR titrations):

Stock solutions of cryptand and metal salts (NaBArF or LiBArF) in CD_3CN were prepared. The precise quantity of cryptand or metal salt was determined with 1,4-dinitrobenzene as internal standard. A 5 mM cryptand stock solution was prepared via dilution. The concentration of metal salt was set to 75 mM with 5 mM cryptand to keep the concentration of cryptand during the titration constantly at 5 mM. 600 µL of cryptand stock solution were added to a standard NMR tube, varying amounts of metal salt stock solution were added. The titration was monitored by ^{1}H NMR spectroscopy (temperature: 298 K). Binding constants were fitted using Bindfit. All raw data, calculated fits and related data can be accessed via supramolecular.org.

2. Synthetic Procedures and Characterization Data

2.1 Syntheses of Orthoesters

Synthesis of (2,2,2-trimethoxyethyl)benzene

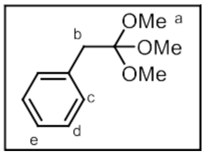

Benzylmagnesium chloride (18.9 mmol, 1.5 equiv., 2.0 M in THF, 9.5 mL) was added dropwise to a solution of 2,2,2-trimethoxyacetonitrile (12.6 mmol, 1.0 equiv., 1.9 mL) in THF (13 ml). The solution was stirred for 40 min, triethylamine was added to prevent hydrolysis. The solvent was removed under reduced pressure, diethyl ether (25 ml), water (25 mL) and brine (25 mL) were added. The phases were separated and the aqueous layer was extracted with diethyl ether (3x 25 mL). The combined organic phases were dried over $MgSO_4$, filtered and the solvent was removed under reduced pressure. The crude product was purified by flash chromatography (hexane/CH_2Cl_2/NEt_3 100:10:1) and vacuum distillation (21 mbar, 116 °C) to give the product as yellowish liquid (1.90 g, 77%). Spectral data are in accordance with those reported previously.[6]

^{1}H-NMR (400 MHz, $CDCl_3$, 293 K): δ (ppm) = 7.27 – 7.36 (m, 4H, c/d), 7.20 – 7.25 (m, 1H, e), 3.31 (s, 9H, a), 3.11 (d, J = 0.6 Hz, 2H, b).

^{1}H NMR spectrum (400 MHz, $CDCl_3$, 293 K):

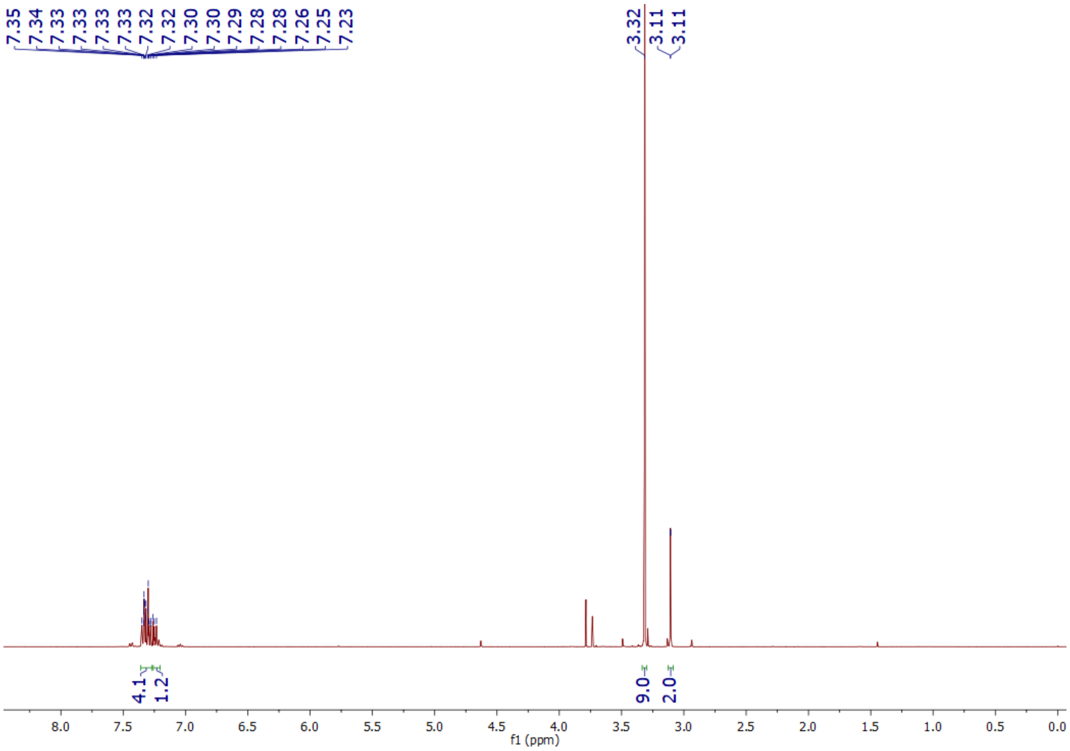

Synthesis of 3,3,3-trimethoxyprop-1-yne

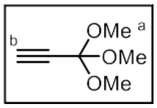

Trimethyl(3,3,3-trimethoxyprop-1-yn-1-yl)silane (2.30 mmol, 1.0 equiv., 465 mg) was suspended in 10% NaOH-solution (17 mL) and stirred for 21 h. Diethyl ether (20 mL) was added, the phases were separated. The aqueous phase was extracted with diethyl ether (2x 20 mL). The combined organic phases were washed with saturated $KHCO_3$-solution (10 mL) and 10% NaOH-solution (10 mL), dried over $MgSO_4$ and filtered. The solvent was removed under reduced pressure to give the product as colourless liquid (290 mg, 97%).

^{1}H-NMR (400 MHz, $CDCl_3$, 293 K): δ (ppm) = 3.39 (s, 9H, a), 2.59 (s, 1H, b).

^{13}C-NMR (100 MHz, $CDCl_3$, 293 K): δ (ppm) = 109.6, 76.5, 72.8, 50.8.

LRMS (ESI$^+$): *m/z* = 153.1 [M+Na]$^+$ (calcd. 153.1 for $C_6H_{10}NaO_3$).

^{1}H NMR spectrum (400 MHz, $CDCl_3$, 293 K):

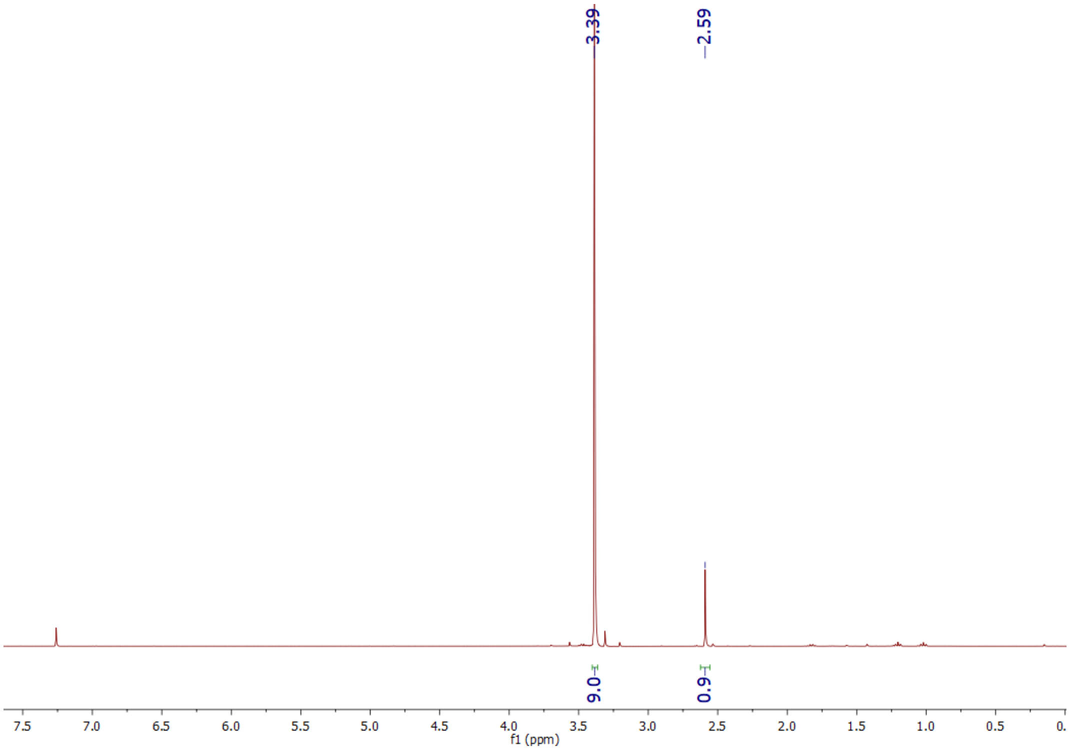

^{13}C NMR spectrum (100 MHz, $CDCl_3$, 293 K):

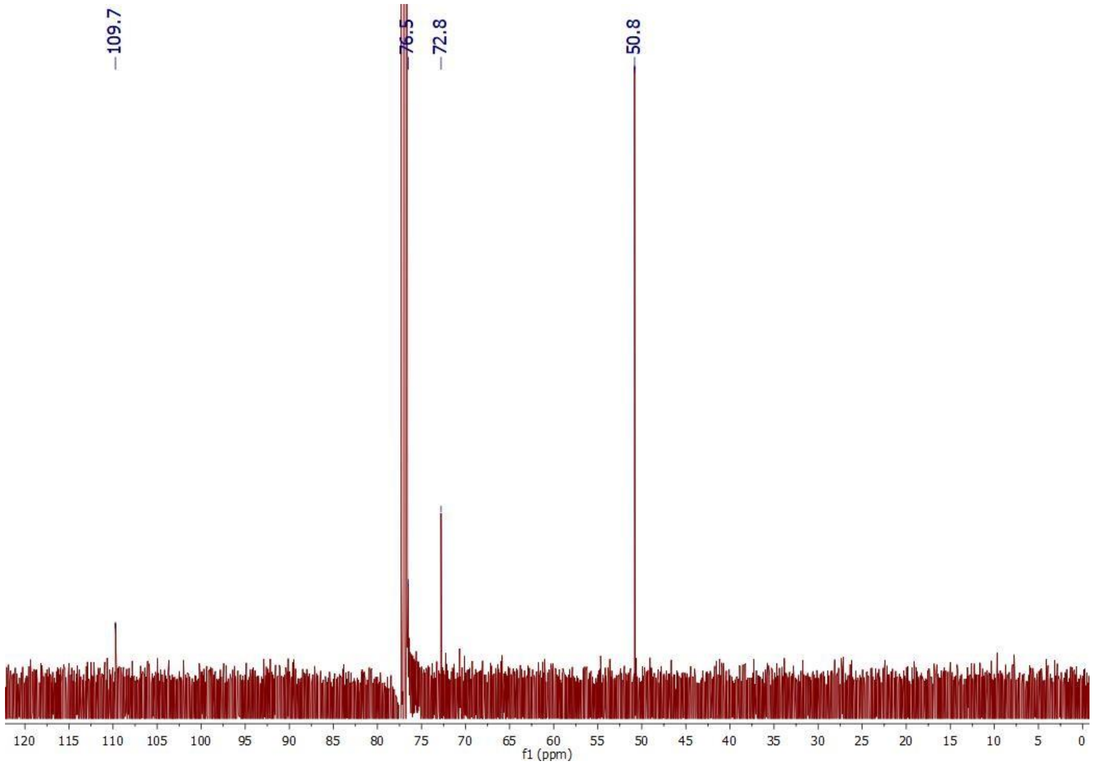

Synthesis of 1-benzyl-4-(trimethoxymethyl)-1*H*-1,2,3-triazole

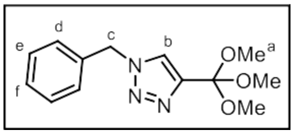

3,3,3-trimethoxyprop-1-yne (766 µmol, 1.0 equiv., 100 µL), $Cu(MeCN)_4PF_6$ (7.66 µmol, 0.01 equiv., 2.9 mg) and TBTA (7.66 µmol, 0.01 equiv., 4.1 mg) were dissolved in anhydrous methanol (10 mL). Benzyl azide (766 µmol, 1.0 equiv., 0.5 M in CH_2Cl_2, 1.53 mL) was added. The reaction mixture was heated to 70 °C for 48 h. The solvent was removed under reduced pressure and the crude product was purified by passing it over a short plug of silica gel. The plug was purged with acetonitrile, removal of the solvent gave the product as colourless solid (181 mg, 90%).

^{1}H NMR (400 MHz, CD_3CN, 293 K): δ (ppm) = 7.81 (s, 1H, b), 7.40 – 7.33 (m, 3H, d-f), 7.32 – 7.26 (m, 2H, d-f), 5.55 (s, 2H, c), 3.13 (s, 9H, a).

^{13}C-NMR (100 MHz, CD_3CN, 293 K): δ (ppm) = 145.3, 136.8, 129.9, 129.3, 128.8, 126.0, 113.2, 54.4, 50.2.

HRMS (ESI$^+$): *m/z* = 286.1162 $[M+Na]^+$ (calcd. 286.1162 for $C_{13}H_{17}N_3NaO_3$).

^{1}H NMR spectrum (400 MHz, CD_3CN, 293 K):

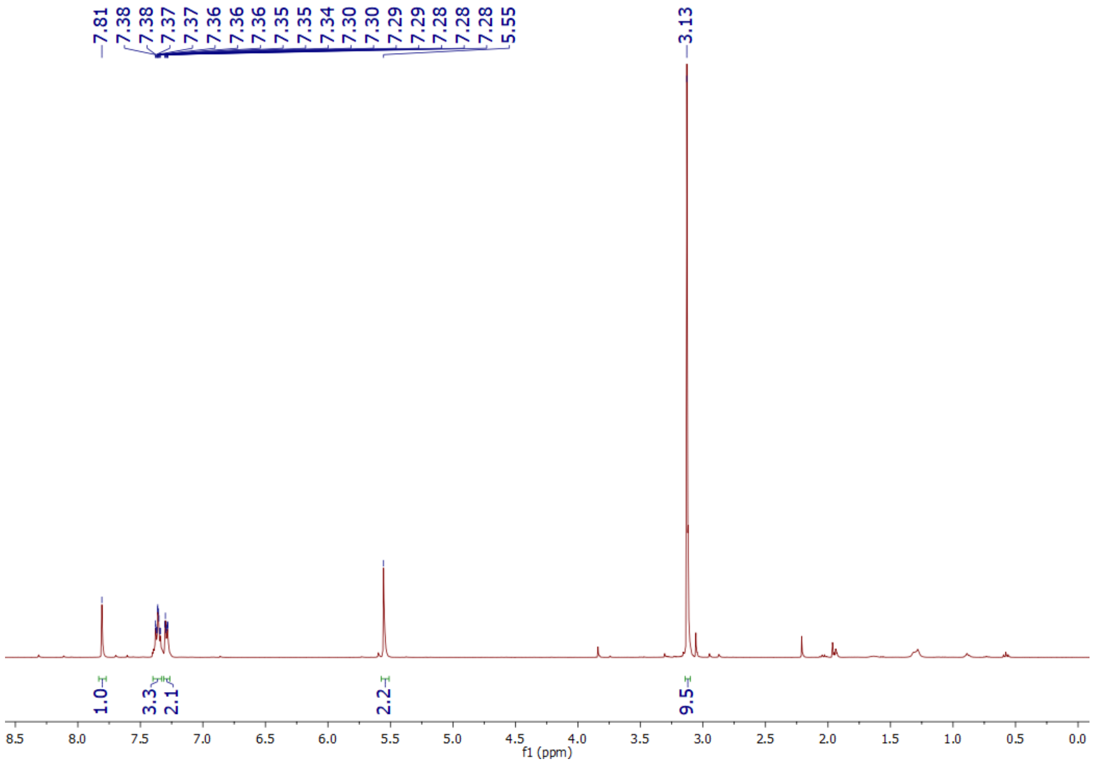

^{13}C NMR spectrum (100 MHz, CD_3CN, 293 K):

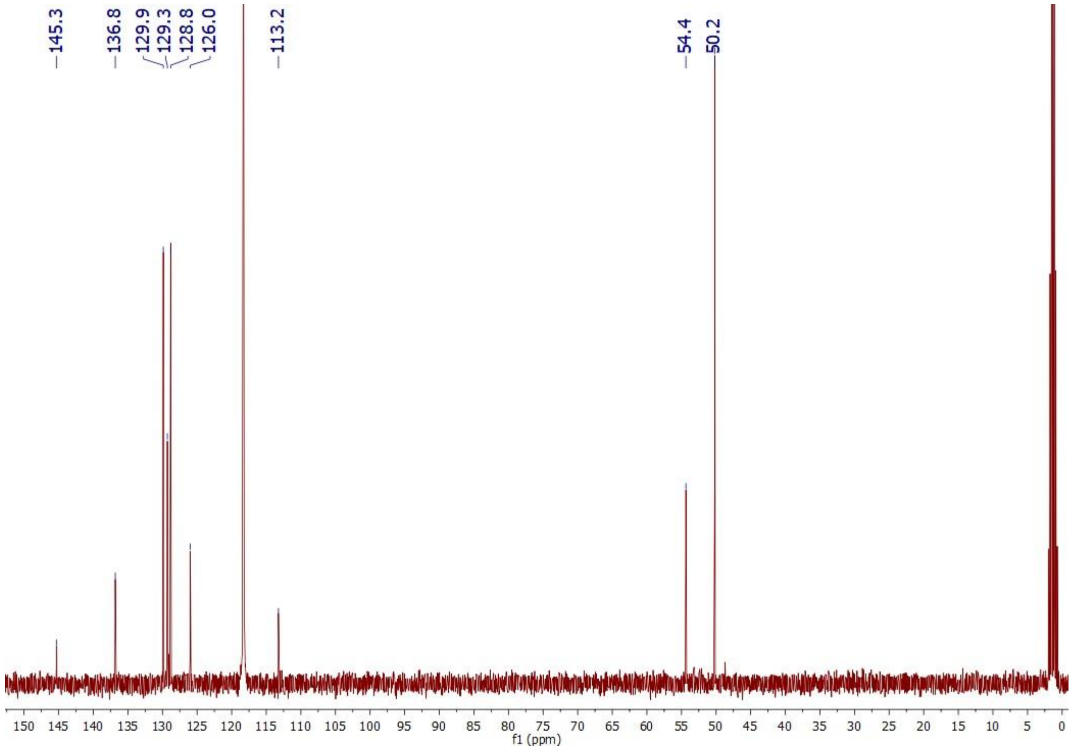

Synthesis of 1-benzyl-3-methyl-4-(trimethoxymethyl)-1*H*-1,2,3-triazol-3-ium methyl sulfate

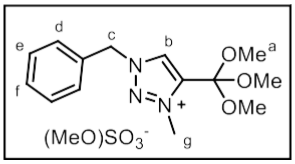

1-benzyl-4-(trimethoxymethyl)-1*H*-1,2,3-triazole (190 µmol, 1.0 equiv., 50 mg) was dissolved in anhydrous acetonitrile (5 mL). Dimethyl sulfate (190 µmol, 1.0 equiv., 18.0 µL) was added, the reaction mixture was heated to 80 °C for 3 d. The solvent was removed under reduced pressure to give the corresponding salt as slightly yellow oil (72 mg, 97%).

^{1}H NMR (400 MHz, CD_3CN, 293 K): δ (ppm) = 8.74 – 8.65 (m, 1H, b), 7.56 – 7.51 (m, 2H, d-f), 7.47 – 7.44 (m, 3H, d-f), 5.82 – 5.78 (m, 2H, c), 4.20 (s, 3H, g), 3.51 (s, 3H, $(MeO)SO3^-$), 3.22 (s, 9H, a).

^{13}C NMR (100 MHz, CD_3CN, 293 K): δ (ppm) = 139.9, 132.9, 132.7, 130.6, 130.5, 130.2, 58.2, 54.0, 51.1, 40.0.

HRMS (ESI$^+$): *m/z* = 278.1498 [M]$^+$ (calcd. 278.1499 for $C_{14}H_{20}N_3O_3$).

^{1}H NMR spectrum (400 MHz, CD_3CN, 293 K):

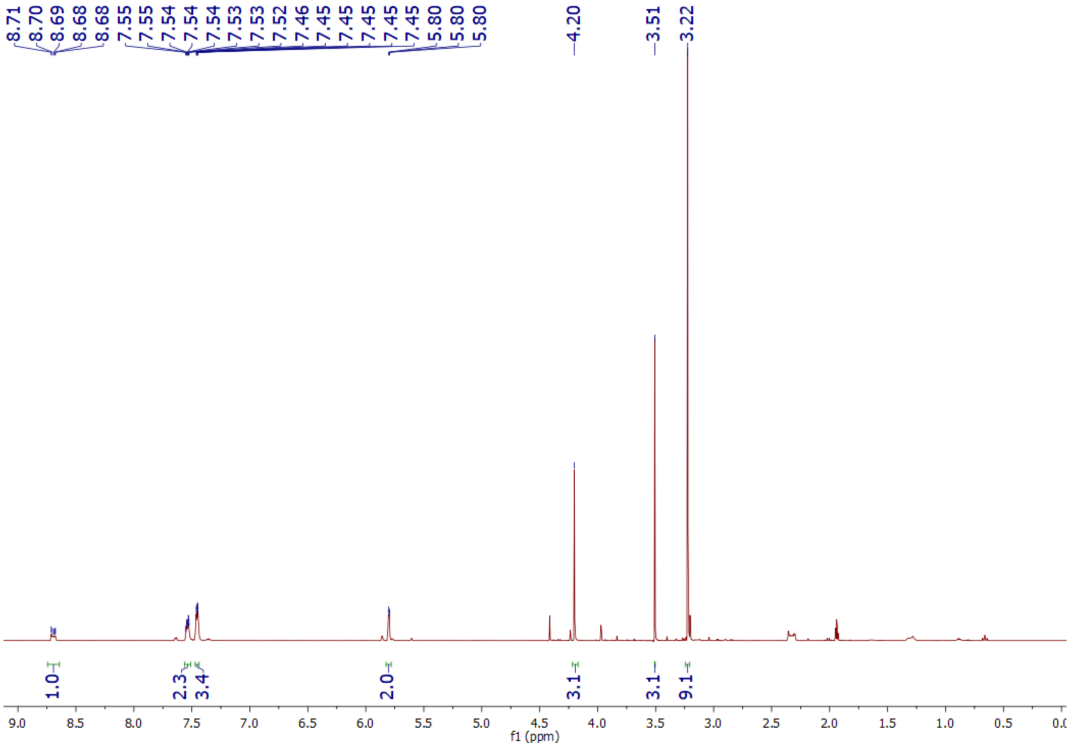

^{13}C NMR spectrum (100 MHz, CD_3CN, 293 K):

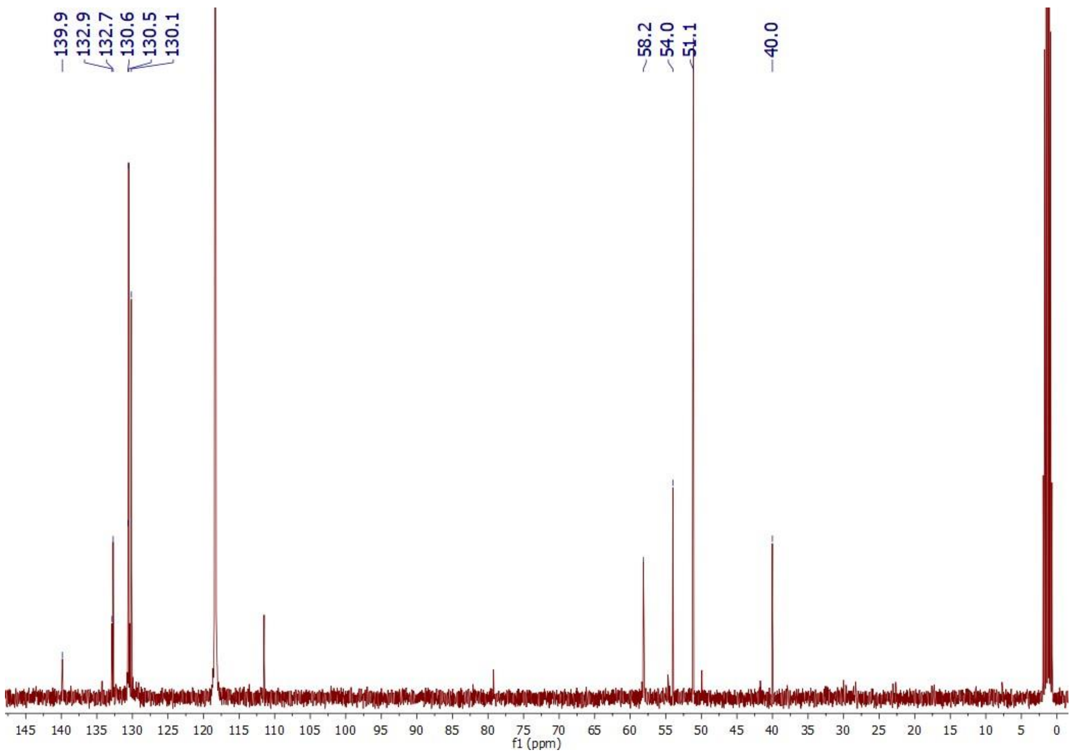

2.2 Synthesis of Orthoester Cryptates

Optimized Synthesis of [Na$^+$⊂*o*-$(CH_3)_2$-1.1.1]BArF$^-$

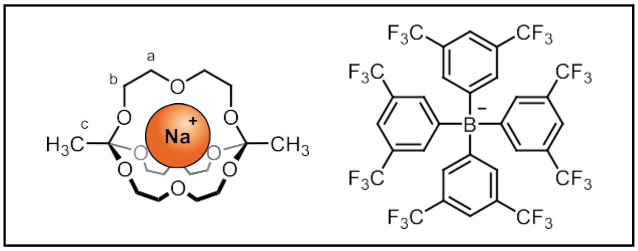

[Na$^+$⊂*o*-$(CH_3)_2$-1.1.1]BArF$^-$ was prepared according to general procedure A. The product was obtained as colourless solid (58 mg, 77%). Spectral data are in accordance with those reported previously.[7]

^{1}H-NMR (500 MHz, $CDCl_3$, 293 K): δ (ppm) = 7.72 – 7.67 (m, 8H), 7.55 – 7.52 (m, 4H), 3.85 – 3.78 (m, 12H, b), 3.55 – 3.47 (m, 12H, a), 1.46 (s, 6H, c).

Synthesis of *o*-$(CH_3)_2$-1.1.1

The synthesis of *o*-$(CH_3)_2$-1.1.1 was reported previously.[7]

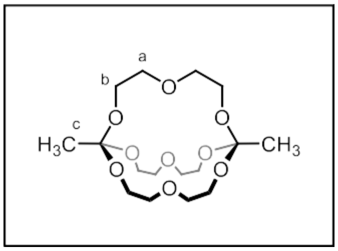

Synthesis of [Na$^+$⊂*o*-$(nC_4H_9)_2$-1.1.1]BArF$^-$

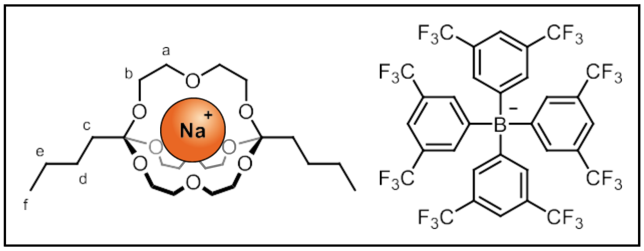

[Na$^+$⊂*o*-$(nC_4H_9)_2$-1.1.1]BArF$^-$ was prepared according to general procedure A. The product was obtained as colourless solid (57 mg, 72%).

^{1}H-NMR (500 MHz, $CDCl_3$, 293 K): δ (ppm) = 7.79 – 7.63 (m, 8H), 7.57 – 7.51 (m, 4H), 3.93 – 3.84 (m, 12H, b), 3.54 – 3.46 (m, 12H, a), 1.92 – 1.78 (m, 4H, c), 1.42 – 1.26 (m, 8H, d-e), 0.90 (t, J = 7.1 Hz, 6H, f).

^{13}C-NMR (125 MHz, $CDCl_3$, 293 K): *δ* (ppm) = 162.3, 161.9, 161.5, 161.1, 134.8, 129.3, 129.3, 129.3, 129.3, 129.1, 129.1, 129.0, 129.0, 128.8, 128.8, 128.8, 128.8, 128.6, 128.6, 128.5, 128.5, 127.8, 125.6, 123.5, 121.3, 117.5, 117.4, 117.4, 113.7, 69.5, 62.4, 33.7, 25.1, 22.6, 13.7.

HRMS (ESI$^+$): *m/z* = 473.2714 $[M+Na]^+$ (calcd. 473.2721 for $C_{22}H_{42}NaO_9$).

^{1}H NMR spectrum (500 MHz, $CDCl_3$, 293 K):

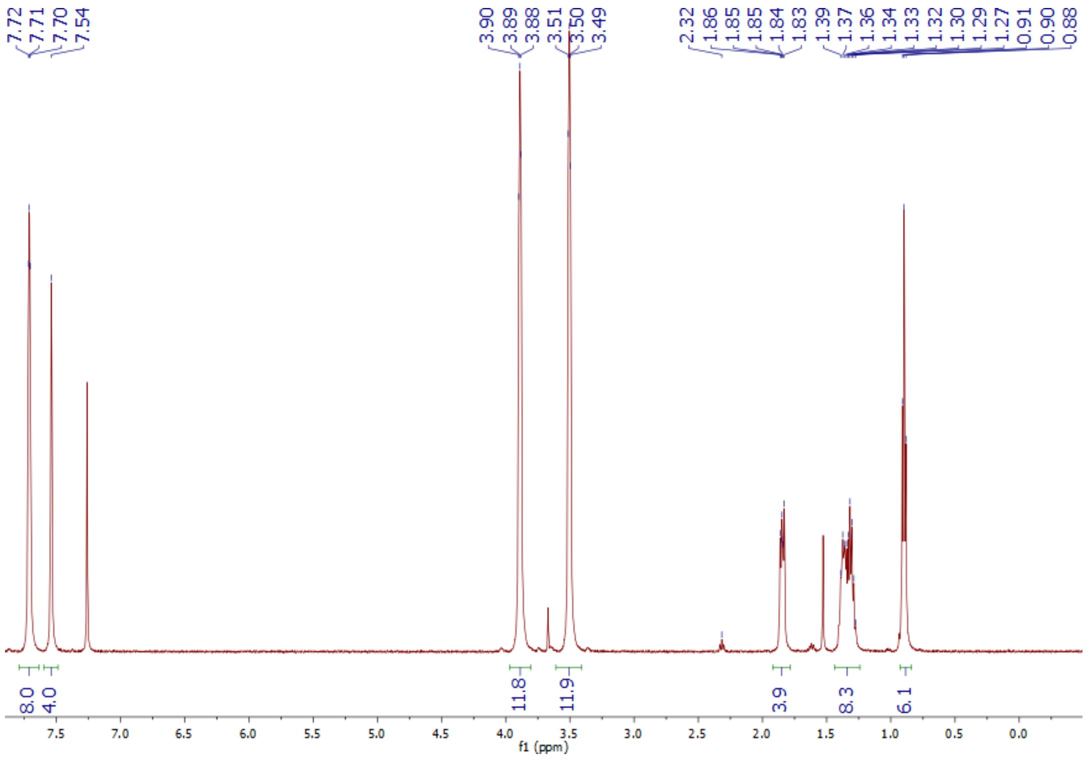

^{13}C NMR spectrum (125 MHz, $CDCl_3$, 293 K):

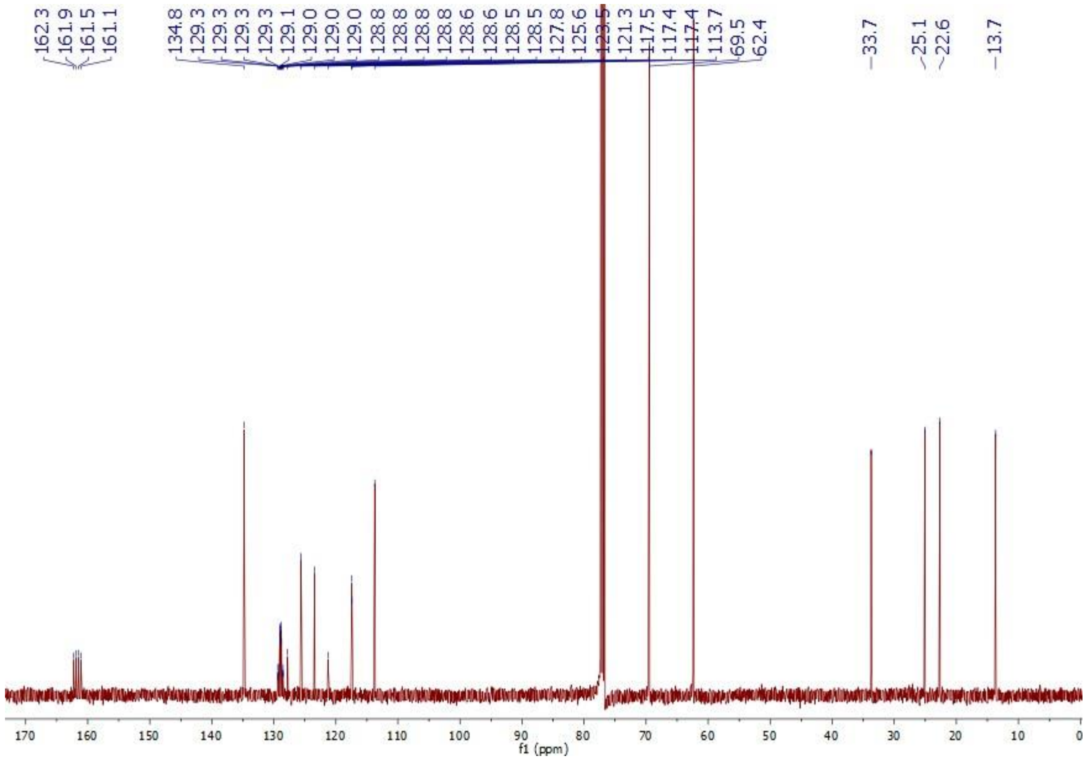

Synthesis of [Na$^+$⊂*o*-($CH_2C_6H_5$)$_2$-1.1.1]BArF$^-$

[Na$^+$⊂*o*-($CH_2C_6H_5$)$_2$-1.1.1]BArF$^-$ was prepared according to general procedure A. The product was obtained as colourless solid (65 mg, 77%).

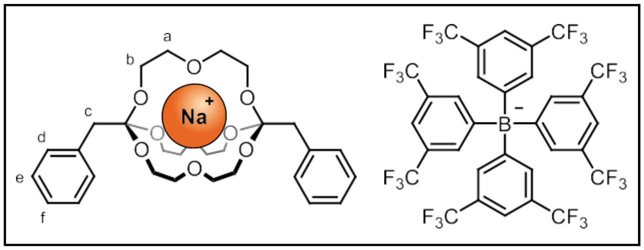

^{1}H-NMR (500 MHz, $CDCl_3$, 293 K): δ (ppm) = 7.74 – 7.71 (m, 8H), 7.55 – 7.53 (m, 4H), 7.32 – 7.27 (m, 6H, d-f), 7.21 – 7.17 (m, 4H, d-f), 3.80 – 3.75 (m, 12H, b), 3.50 – 3.44 (m, 12H, a), 3.10 (s, 4H, c).

^{13}C-NMR (125 MHz, $CDCl_3$, 293 K): δ (ppm) = 162.3, 161.9, 161.5, 161.1, 134.8, 132.6, 130.2, 129.2, 129.1, 129.1, 129.0, 129.0, 128.8, 128.8, 128.8, 128.7, 128.6, 128.6, 128.5, 128.4, 128.3, 127.8, 125.6, 123.5, 121.3, 117.5, 117.5, 117.4, 113.1, 69.3, 62.8, 39.1.

HRMS (ESI$^+$): *m/z* = 541.2398 [M+Na]$^+$ (calcd. 541.2408 for $C_{28}H_{38}NaO_9$).

^{1}H NMR spectrum (500 MHz, $CDCl_3$, 293 K):

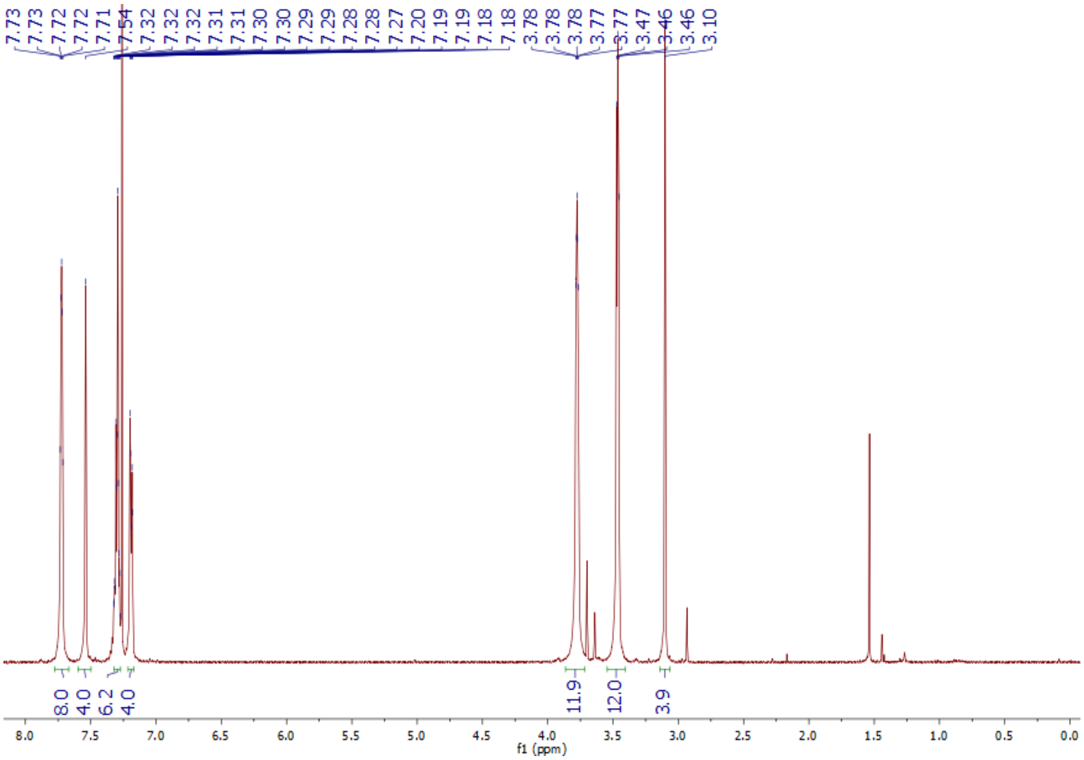

^{13}C NMR spectrum (125 MHz, $CDCl_3$, 293 K):

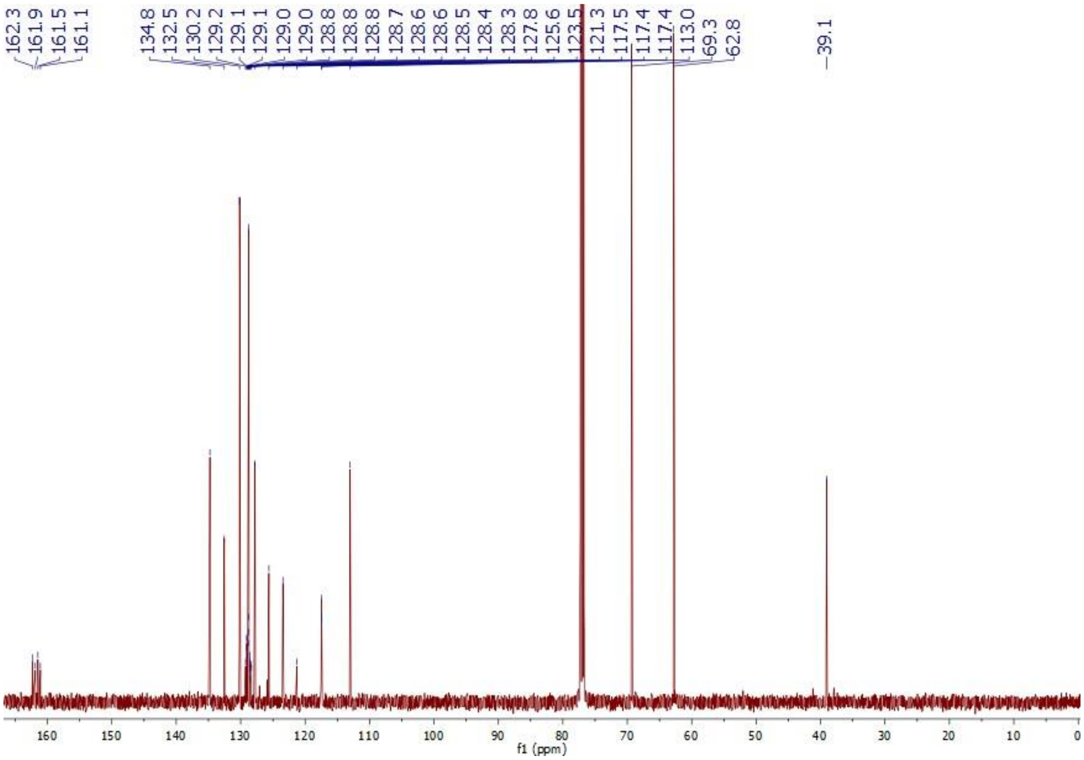

Synthesis of [Na$^+$⊂*o*-(C_6H_5)$_2$-1.1.1]BArF$^-$

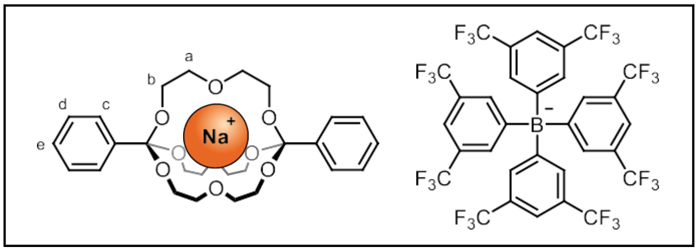

[Na$^+$⊂*o*-(C_6H_5)$_2$-1.1.1]BArF$^-$ was prepared according to general procedure A. The product was obtained as colourless solid (55 mg, 67%).

^{1}H-NMR (500 MHz, $CDCl_3$, 293 K): δ(ppm) = 7.72 – 7.68 (m, 8H), 7.53 – 7.51 (m, 4H), 7.47 – 7.41 (m, 6H, c-e), 7.40 – 7.36 (m, 4H, c-e), 4.04 – 3.97 (m, 12H, b), 3.68 – 3.61 (m, 12H, a).

^{13}C-NMR (125 MHz, $CDCl_3$, 293 K): *δ* (ppm) = 162.3, 161.9, 161.5, 161.1, 134.8, 130.4, 129.6, 129.3, 129.3, 129.3, 129.0, 128.8, 128.8, 128.8, 126.3, 125.6, 123.5, 117.5, 117.5, 117.4, 117.4, 112.4, 69.9, 63.5.

HRMS (ESI$^+$): *m/z* = 513.2093 [M+Na]$^+$ (calcd. 513.2095 for $C_{26}H_{34}NaO_9$).

^1H NMR spectrum (500 MHz, $CDCl_3$, 293 K):

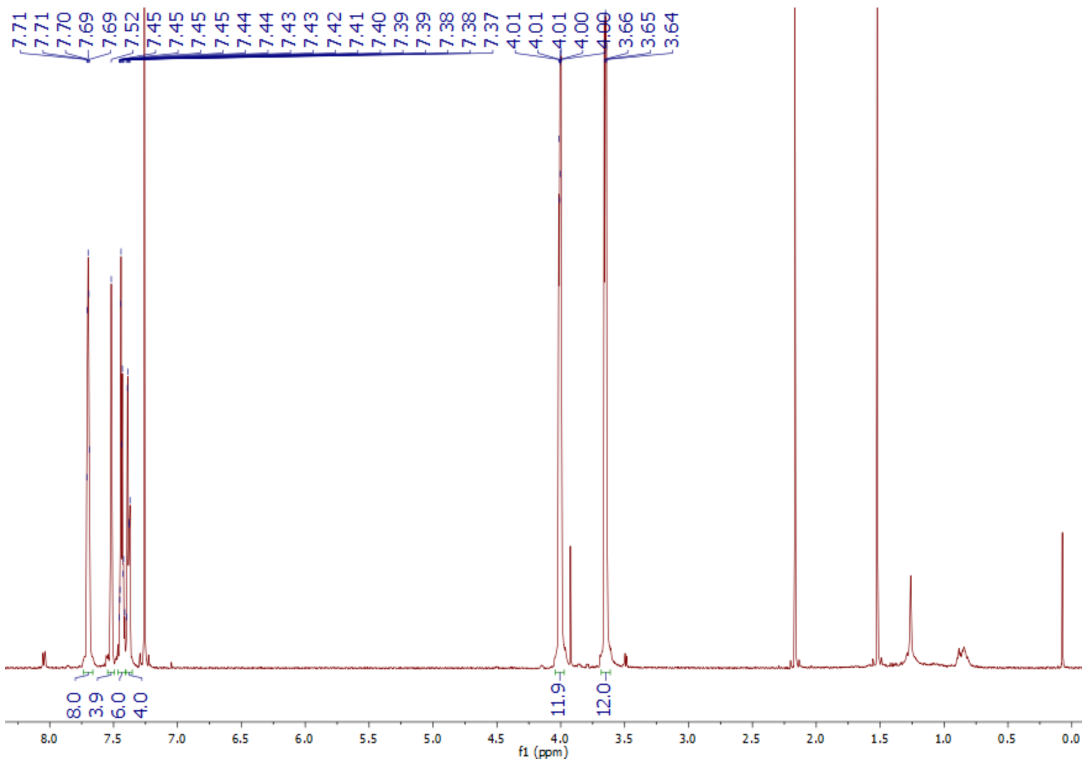

^{13}C NMR spectrum (125 MHz, $CDCl_3$, 293 K):

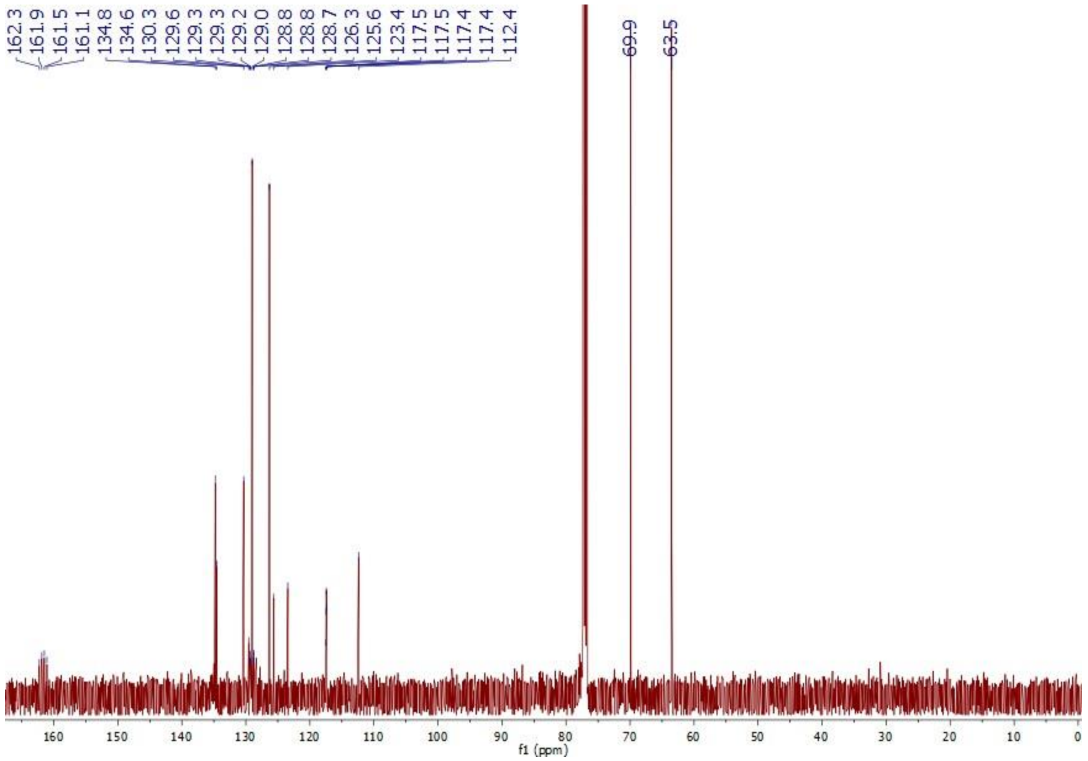

Synthesis of [Na$^+$⊂*o*-(C≡C-Si(CH$_3$)$_3$)$_2$-1.1.1]BArF$^-$

[Na$^+$⊂*o*-(C≡C-Si(CH$_3$)$_3$)$_2$-1.1.1]BArF$^-$ was prepared according to general procedure A. The product was obtained as colourless solid (38 mg, 50%).

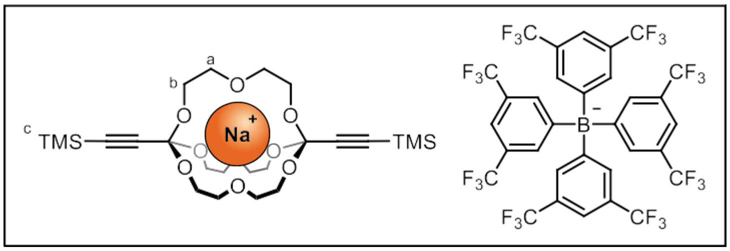

^{1}H-NMR (400 MHz, CDCl$_3$, 293 K): δ (ppm) = 7.73 – 7.68 (m, 8H), 7.55 – 7.50 (m, 4H), 4.00 – 3.85 (m, 12H, b), 3.58 – 3.49 (m, 12H, a), 0.20 (s, 18H, c).

^{13}C-NMR (100 MHz, CDCl$_3$, 293 K): *δ* (ppm) = 134.8, 129.0, 128.7, 125.9, 123.2, 117.4, 103.8, 93.5, 93.2, 68.7, 62.6, -0.7.

HRMS (ESI$^+$): *m/z* = 553.2266 [M+Na]$^+$ (calcd. 553.2260 for $C_{24}H_{42}NaO_9Si_2$).

^{1}H NMR spectrum (400 MHz, CDCl$_3$, 293 K):

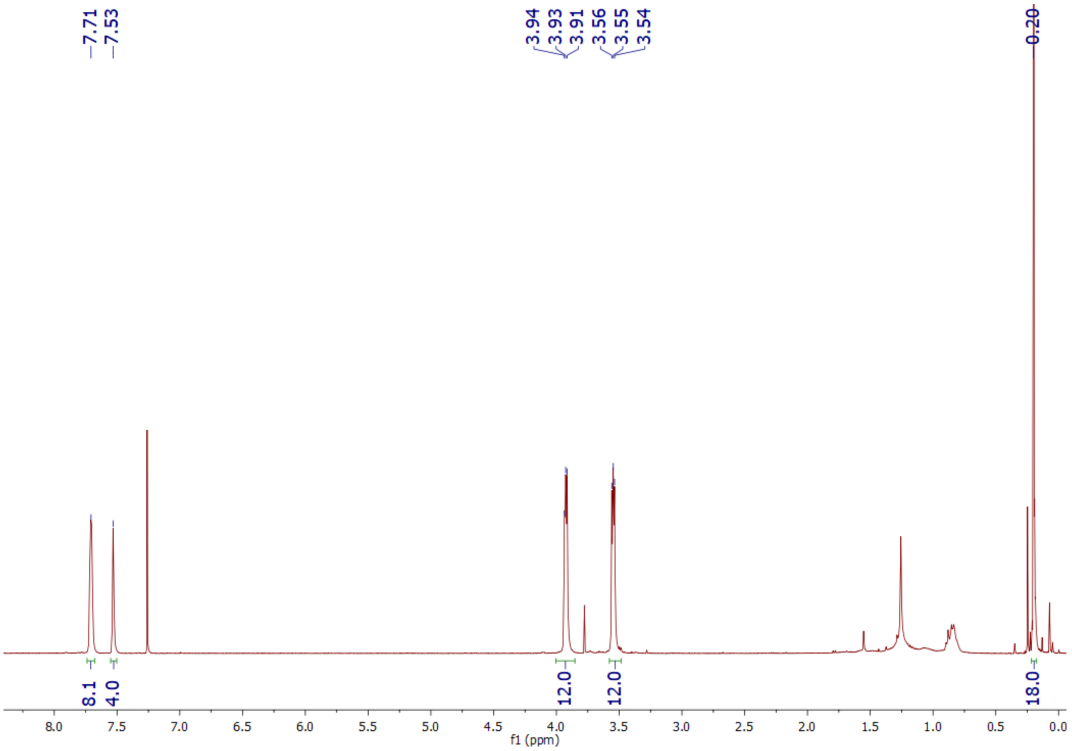

^{13}C NMR spectrum (100 MHz, $CDCl_3$, 293 K):

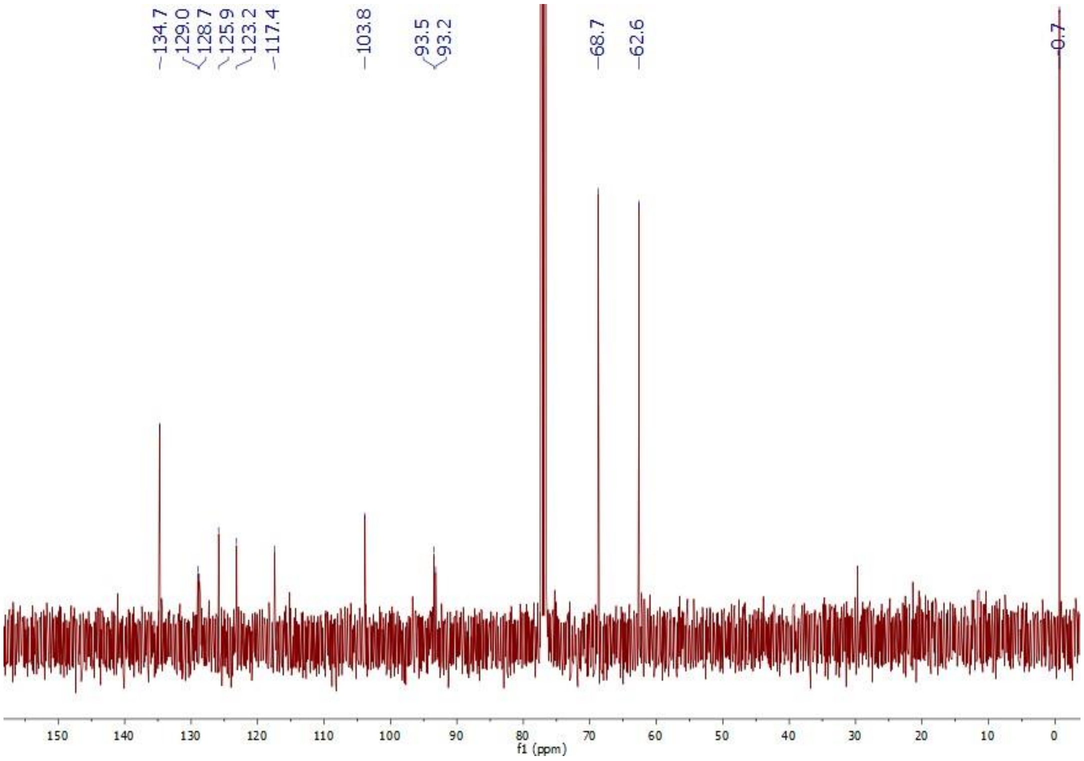

Synthesis of [Na^+⊂*o*-$(CH_2Cl)_2$-1.1.1]$BArF^-$

[Na^+⊂*o*-$(CH_2Cl)_2$-1.1.1]$BArF^-$ was prepared according to general procedure A. The product was obtained as colourless solid (73 mg, 92%).

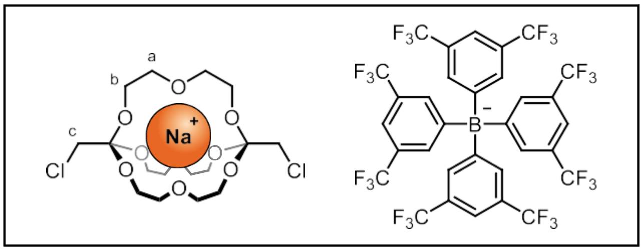

^1H-NMR (400 MHz, $CDCl_3$, 293 K): δ (ppm) = 7.75 – 7.66 (m, 8H), 7.54 (s, 4H), 4.07 – 3.97 (m, 12H, b), 3.69 (s, 4H, c), 3.50 – 3.57 (m, 12H, a).

^{13}C-NMR (100 MHz, $CDCl_3$, 293 K): δ (ppm) = 162.4, 161.9, 161.4, 160.9, 134.7, 129.1, 129.1, 129.0, 129.0, 128.8, 128.8, 128.7, 128.7, 128.6, 125.9, 123.1, 120.4, 117.5, 117.5, 117.5, 117.4, 110.2, 69.1, 63.3, 42.9.

HRMS (ESI^+): *m/z* = 457.0999 $[M+Na]^+$ (calcd. 457.1003 for $C_{16}H_{28}Cl_2NaO_9$).

^{1}H NMR spectrum (400 MHz, $CDCl_3$, 293 K):

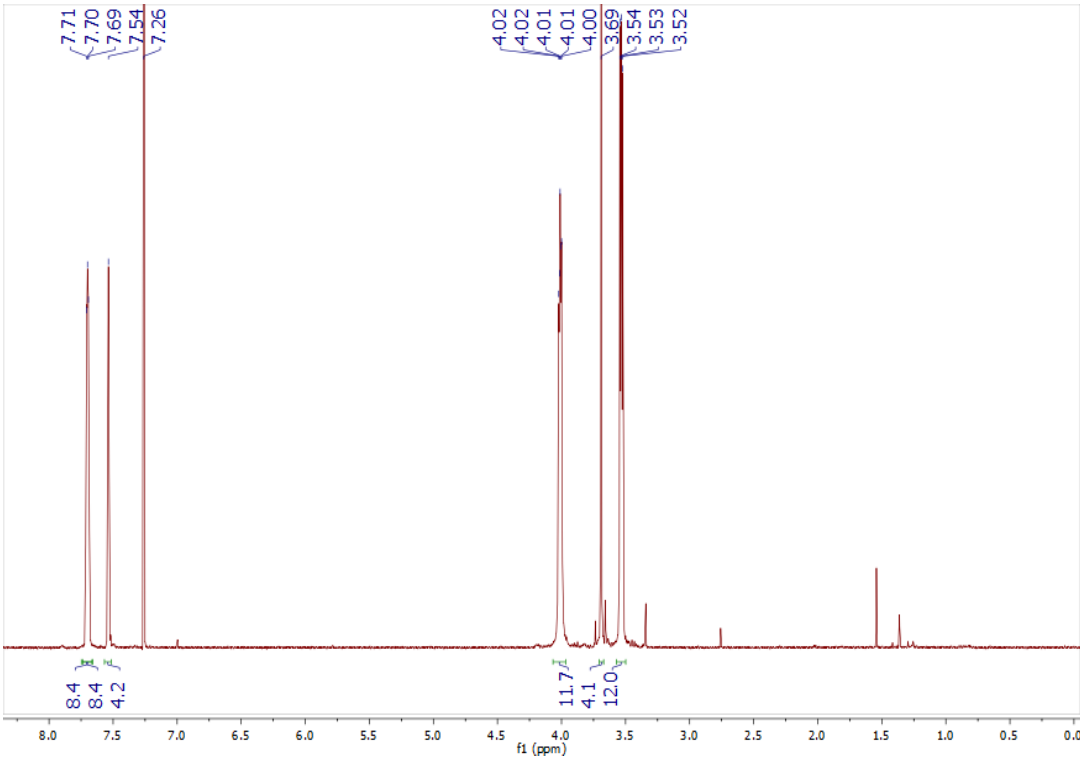

^{13}C NMR spectrum (100 MHz, $CDCl_3$, 293 K):

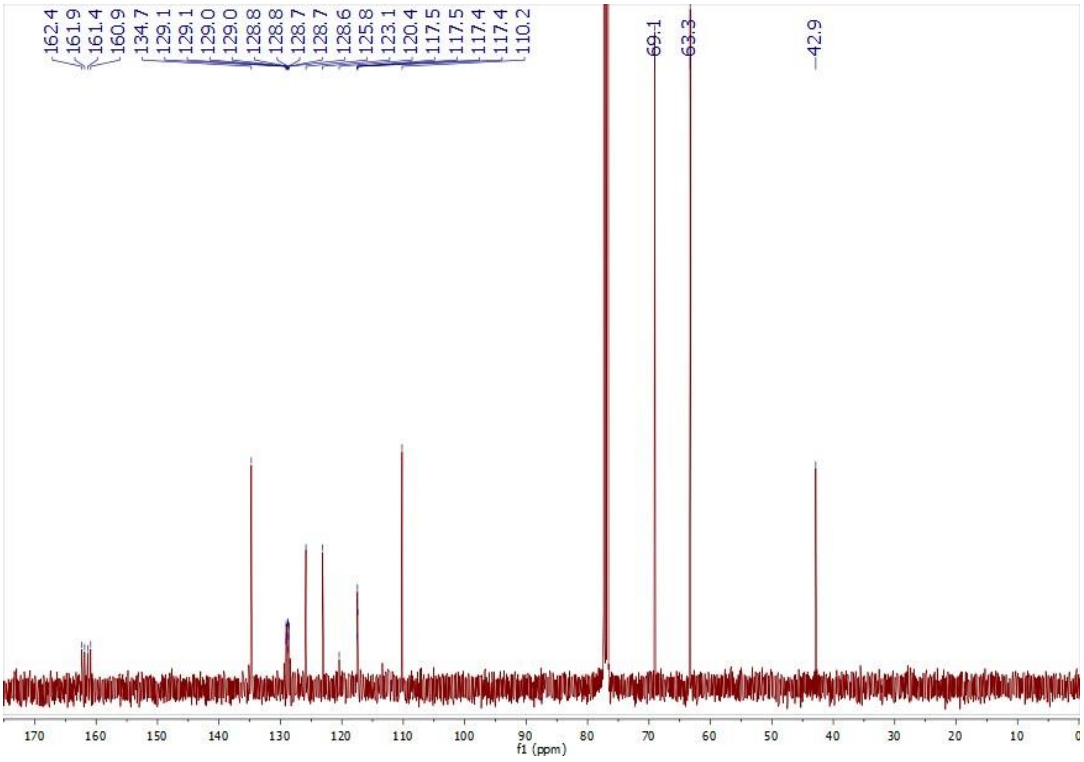

Synthesis of [Na^+⊂*o*-(C≡CH)$_2$-1.1.1]BArF$^-$

[Na^+⊂*o*-(C≡C-Si(CH_3)$_3$)$_2$-1.1.1]BArF$^-$ (28.9 µmol, 1.0 equiv., 41 mg) was dissolved in MeOH (5 mL), K_2CO_3 (86.7 µmol, 3.0 equiv., 12.0 mg) was added and the reaction mixture was stirred for 15 h. The solvent was removed under reduced pressure, H_2O (5 mL) and $CHCl_3$ (5 mL) were added. The phases were separated and the aqueous layer was extracted with $CHCl_3$ (3x 5 mL). The combined organic phases were dried over $MgSO_4$, filtered and the solvent was removed under reduced pressure to give the product as colourless solid (34 mg, 92%).

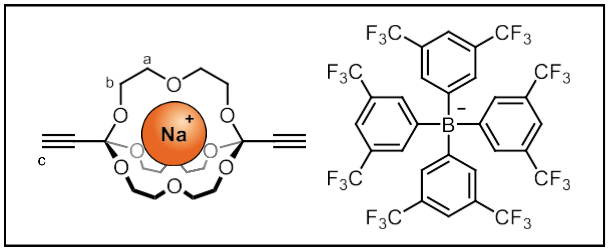

^{1}H-NMR (400 MHz, $CDCl_3$, 293 K): δ (ppm) = 7.73 – 7.69 (m, 8H), 7.57 – 7.51 (m, 4H), 3.96 – 3.93 (m, 12H, b), 3.59 – 3.53 (m, 12H, a), 2.69 (s, 2H, c).

^{13}C-NMR (100 MHz, $CDCl_3$, 293 K): δ (ppm) = 162.4, 161.9, 161.4, 160.9, 134.8, 134.7, 129.1, 129.1, 129.0, 129.0, 128.8, 128.8, 128.7, 128.7, 125.9, 123.1, 117.4, 103.8, 75.3, 73.3, 68.7, 62.7.

HRMS (ESI$^+$): m/z = 409.1480 [M+Na]$^+$ (calcd. 409.1469 for $C_{18}H_{26}NaO_9$).

^{1}H NMR spectrum (400 MHz, $CDCl_3$, 293 K):

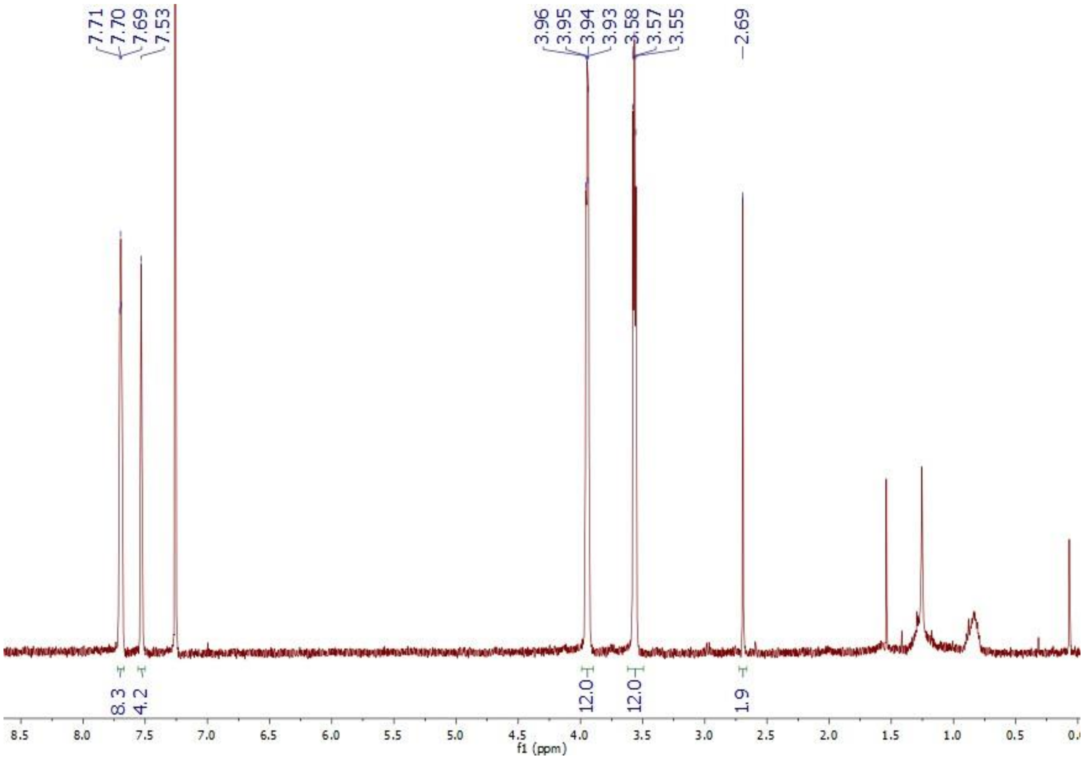

^{13}C NMR spectrum (100 MHz, $CDCl_3$, 293 K):

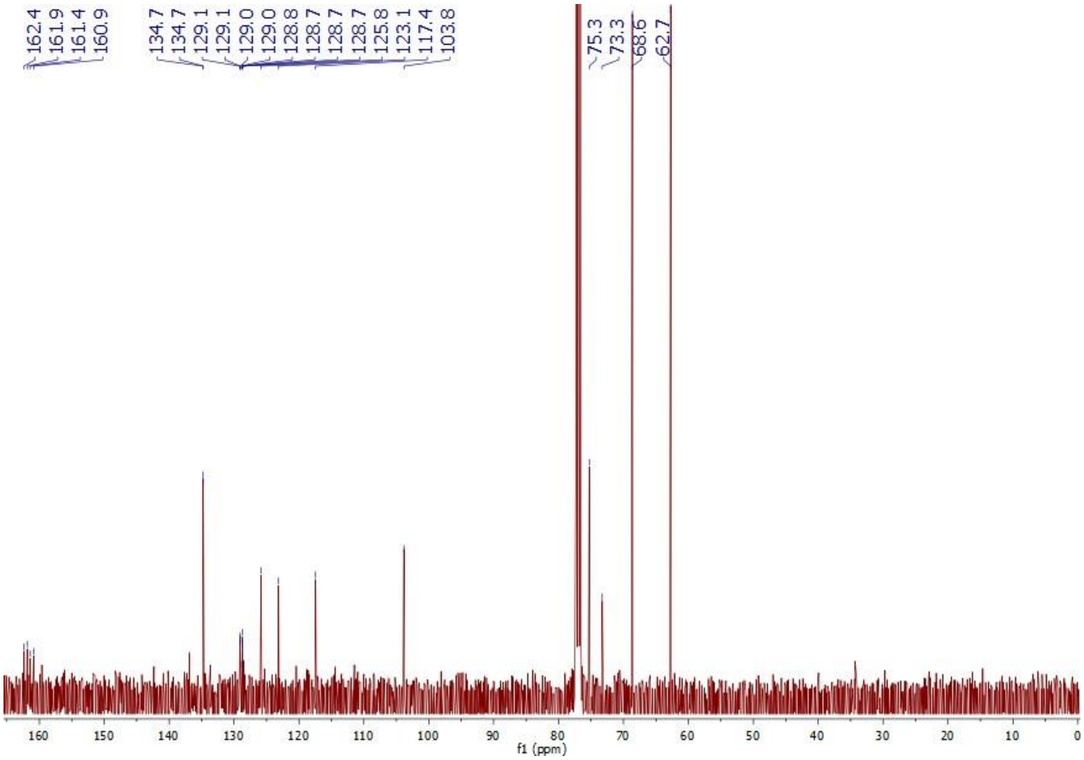

Synthesis of [$Li^+\subset$*o*-$(H)_2$-1.1.1]BArF$^-$

[$Li^+\subset$*o*-$(H)_2$-1.1.1]BArF$^-$ was prepared according to general procedure A. The product was obtained as colourless solid (33 mg, 45%).

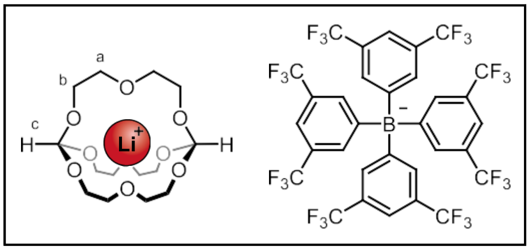

^{1}H-NMR (500 MHz, $CDCl_3$, 293 K): δ (ppm) = 7.72 – 7.68 (m, 8H), 7.55 – 7.53 (m, 4H), 5.32 (s, 2H, c), 3.88 – 3.84 (m, 12H, b), 3.66 – 3.63 (m, 12H, a).

^{13}C-NMR (125 MHz, $CDCl_3$, 293 K): δ (ppm) = 161.1, 148.8, 142.1, 134.8, 125.6, 123.4, 117.5, 111.0, 71.7, 63.4.

HRMS (ESI$^+$): m/z = 345.1755 $[M+Li]^+$ (calcd. 345.1713 for $C_{14}H_{26}LiO_9$).

^{1}H NMR spectrum (500 MHz, $CDCl_3$, 293 K):

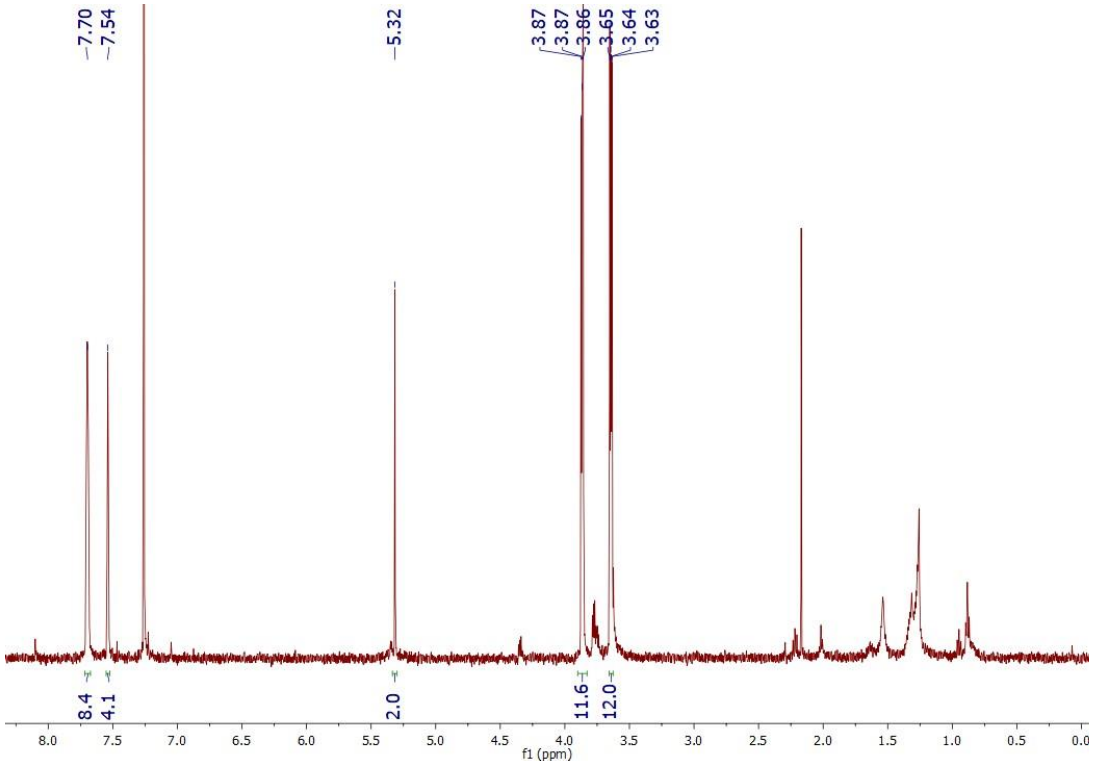

^{13}C NMR spectrum (125 MHz, $CDCl_3$, 293 K):

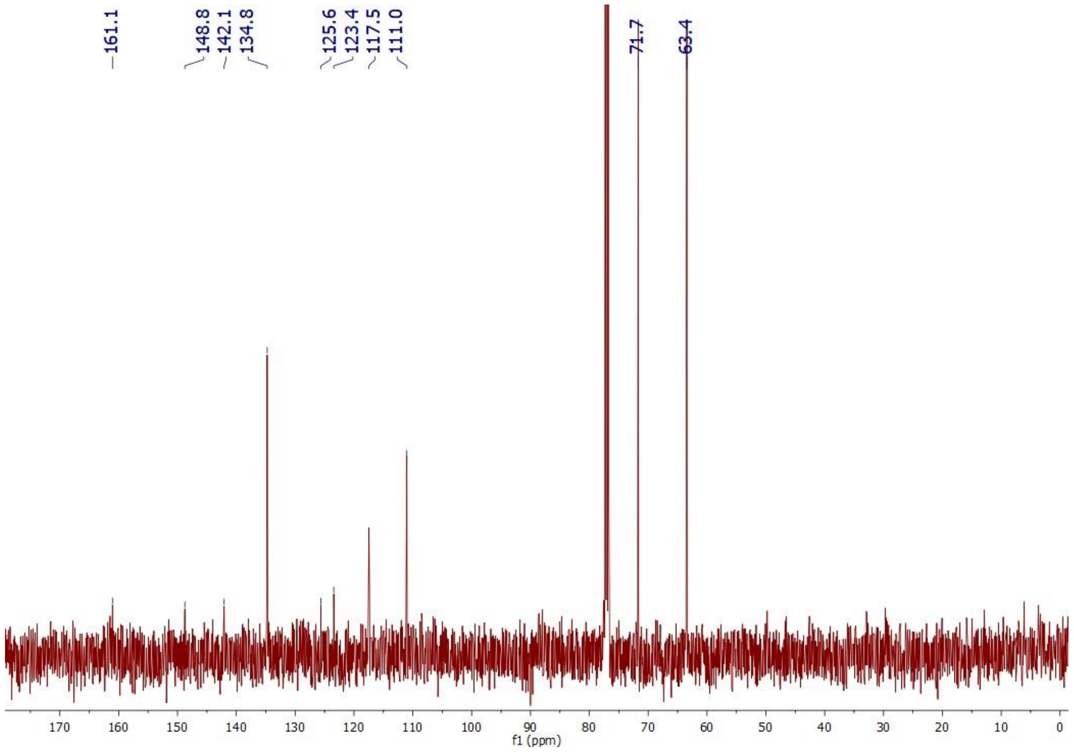

Synthesis of *o*-(H)$_2$-1.1.1

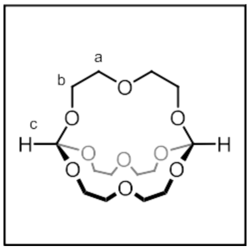

[Li$^+$⊂*o*-(H)$_2$-1.1.1]BArF$^-$ (44.7 µmol, 54 mg) was stirred in $CHCl_3$ with chloride-loaded anion exchange resin (Lewatite MP-64) for 12 h. The resin was removed by filtration and the solvent was removed under reduced pressure to give the product as colourless oil (15 mg, quant.).

^{1}H-NMR (500 MHz, $CDCl_3$, 293 K): δ (ppm) = 5.26 (s, 2H, c), 3.80 (s, 24H, a-b).

^{13}C-NMR (125 MHz, $CDCl_3$, 293 K): δ (ppm) = 111.9, 70.5, 63.8.

^{1}H NMR spectrum (500 MHz, $CDCl_3$, 293 K):

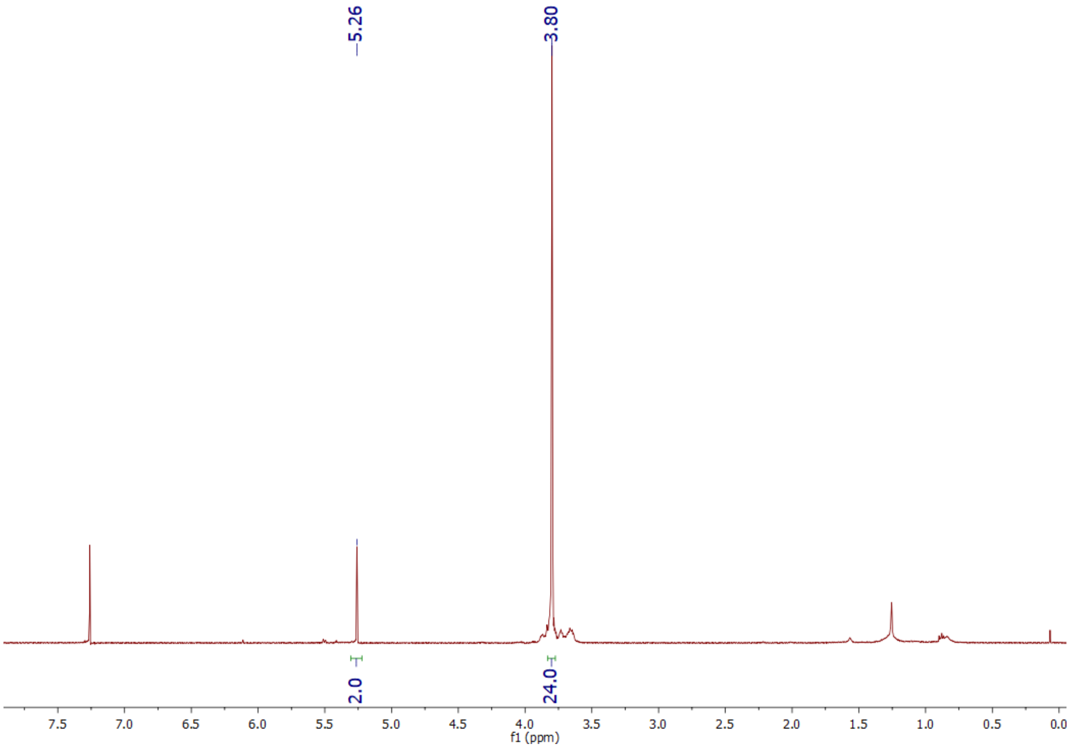

^{13}C NMR spectrum (125 MHz, $CDCl_3$, 293 K):

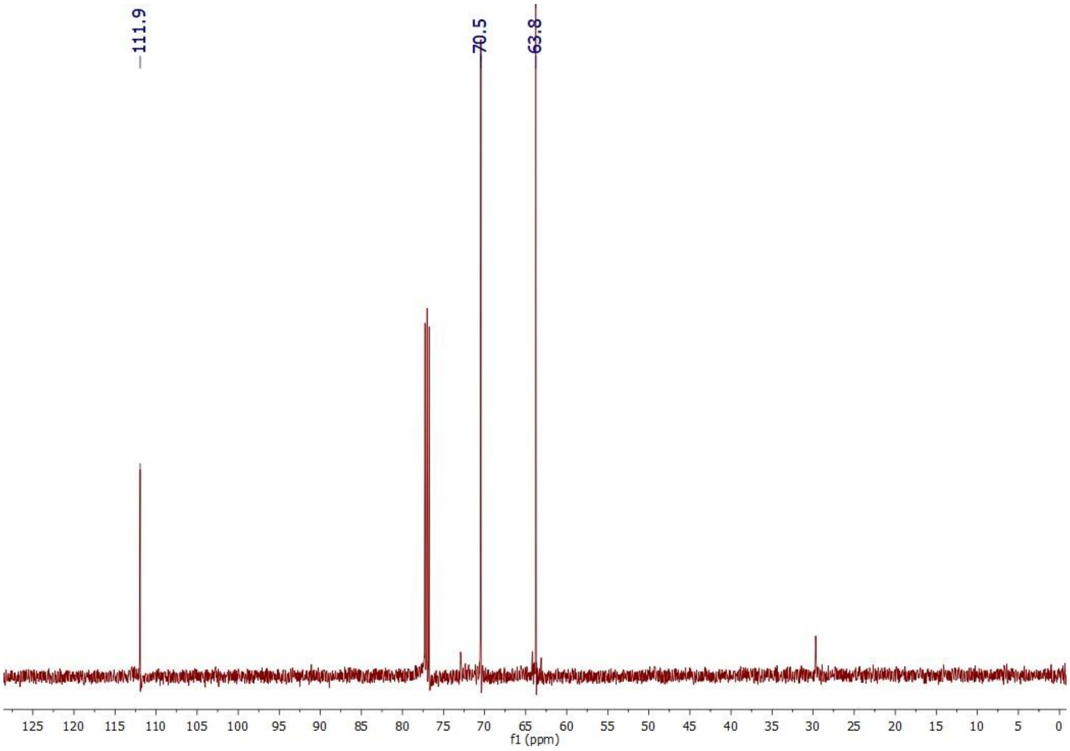

2.3 Post-Functionalization

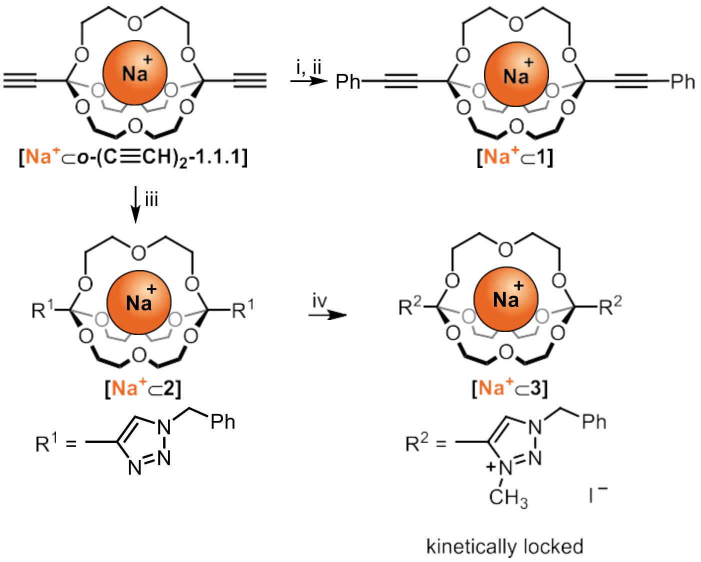

Scheme S1: Post-functionalization of orthoester cryptates.

Sonogashira Reaction

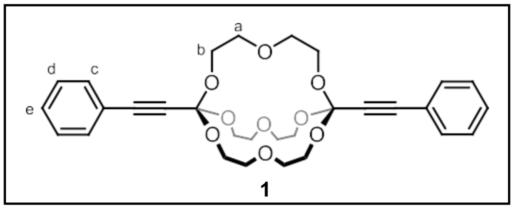

[Na$^+$⊂*o*-(C≡CH)$_2$-1.1.1]BArF$^-$ (11.8 µmol, 1.0 equiv., 15.0 mg) was dissolved in anhydrous THF (1.5 mL), triethylamine (35.4 µmol, 3.0 equiv., 4.98 µL), copper iodide (0.12 µmol, 0.01 equiv., 0.04 mg), tetrakis-(triphenylphosphine)palladium(0) (0.12 µmol, 0.01 equiv., 0.15 mg) and iodobenzene (23.6 µmol, 2.0 equiv., 2.70 µL) were added. The reaction mixture was heated to 40 °C for 4 d. The solvent was removed under reduced pressure. The crude product was purified by passing it over a short plug of silica gel and washing the plug with anhydrous chloroform and triethylamine. Removal of the solvent gave the product **1** as colourless solid (6.0 mg, 94%). Note: this purification procedure resulted in the unintended removal of the guest ion, which can be useful if the cryptand is desired. See below, for reintroduction of sodium.

^{1}H-NMR (500 MHz, $CDCl_3$, 293 K): δ (ppm) = 7.52 – 7.44 (m, 4H, c-e), 7.39 – 7.28 (m, 6H, c-e), 3.99 – 3.96 (m, 12H, b), 3.85 – 3.82 (m, 12H, a).

^{13}C-NMR: (125 MHz, $CDCl_3$, 293 K): δ(ppm) = 132.1, 129.2, 128.3, 121.1, 109.5, 84.1, 82.6, 70.5, 63.9.

^{1}H NMR spectrum (500 MHz, $CDCl_3$, 293 K):

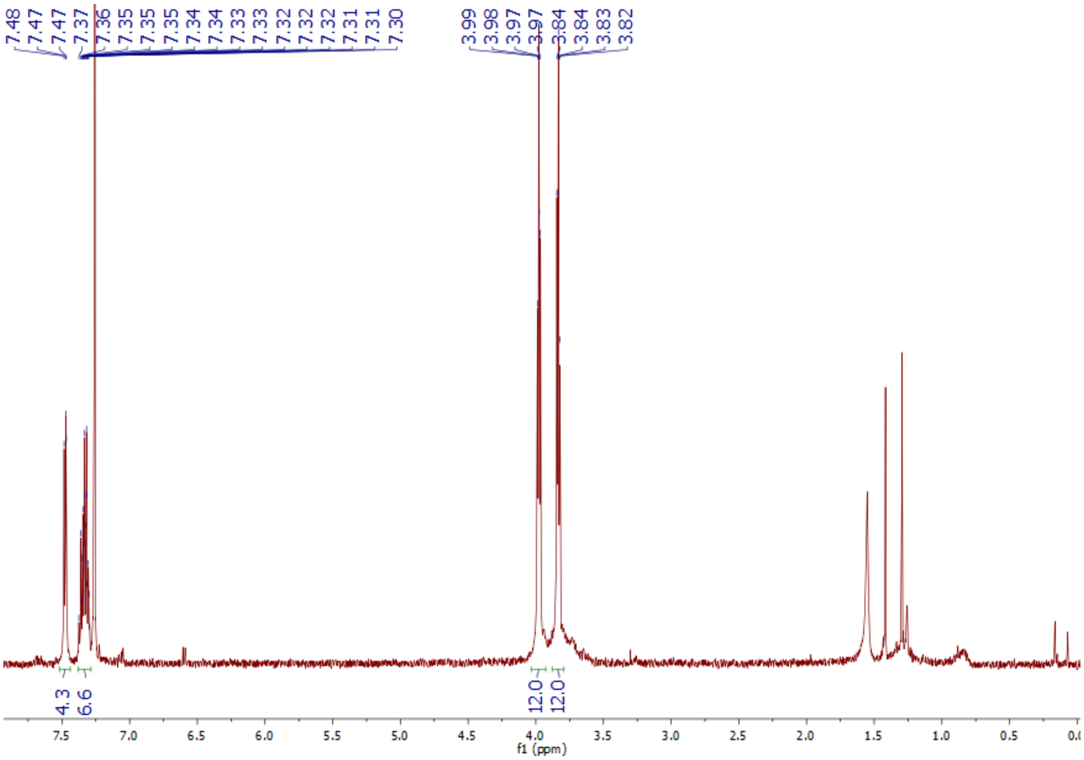

^{13}C NMR spectrum (125 MHz, $CDCl_3$, 293 K):

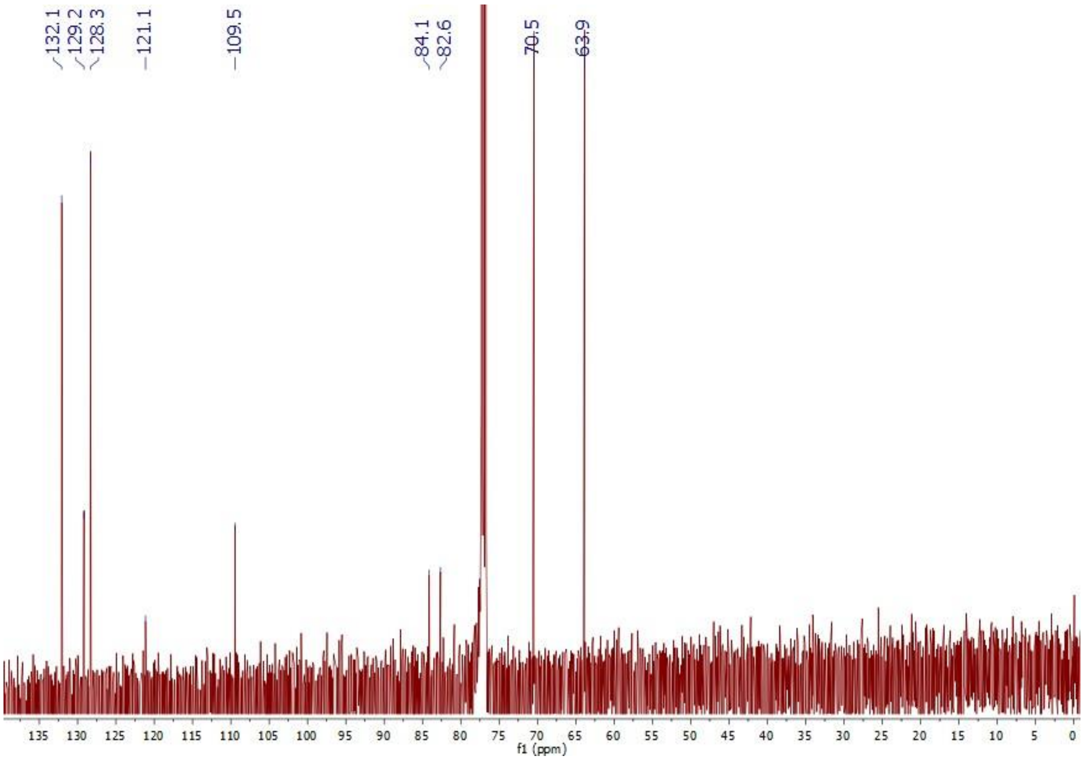

The Sonogashira cryptand **1** (9.28 µmol, 1.0 equiv., 5.0 mg) was dissolved in acetonitrile and NaBArF (9.28 µmol, 1.0 equiv., 8.2 mg) was added. The mixture was stirred for 5 min. The solvent was removed under reduced pressure and the crude product was purified by passing it over a short plug of silica gel. The plug was purged with anhydrous chloroform, removal of the solvent gave the product **[Na$^+$⊂1]BArF$^-$** as colourless solid (13.2 mg, quant.).

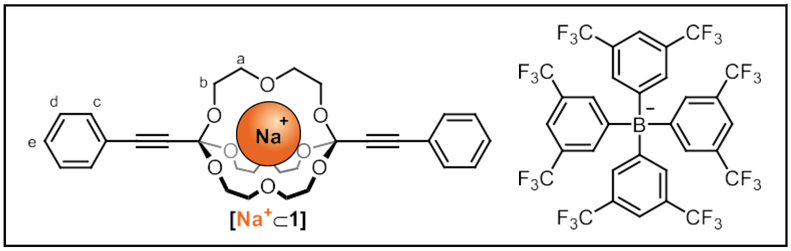

^{1}H-NMR (500 MHz, $CDCl_3$, 293 K): δ (ppm) = 7.71 (s, 8H), 7.52 (s, 4H), 7.49 – 7.41 (m, 6H, c-e), 7.40 – 7.33 (m, 4H, c-e), 4.08 – 4.04 (m, 12H, b), 3.65 – 3.61 (m, 12H, a).

^{13}C-NMR (125 MHz, $CDCl_3$, 293 K): δ (ppm) = 134.8, 132.1, 130.3, 128.6, 125.6, 123.5, 121.3, 117.4, 105.1, 87.2, 68.9, 62.8.

HRMS (ESI$^+$): *m/z* = 561.2104 $[M+Na]^+$ (calcd. 561.2095 for $C_{30}H_{34}NaO_9$).

^{1}H NMR spectrum (500 MHz, $CDCl_3$, 293 K):

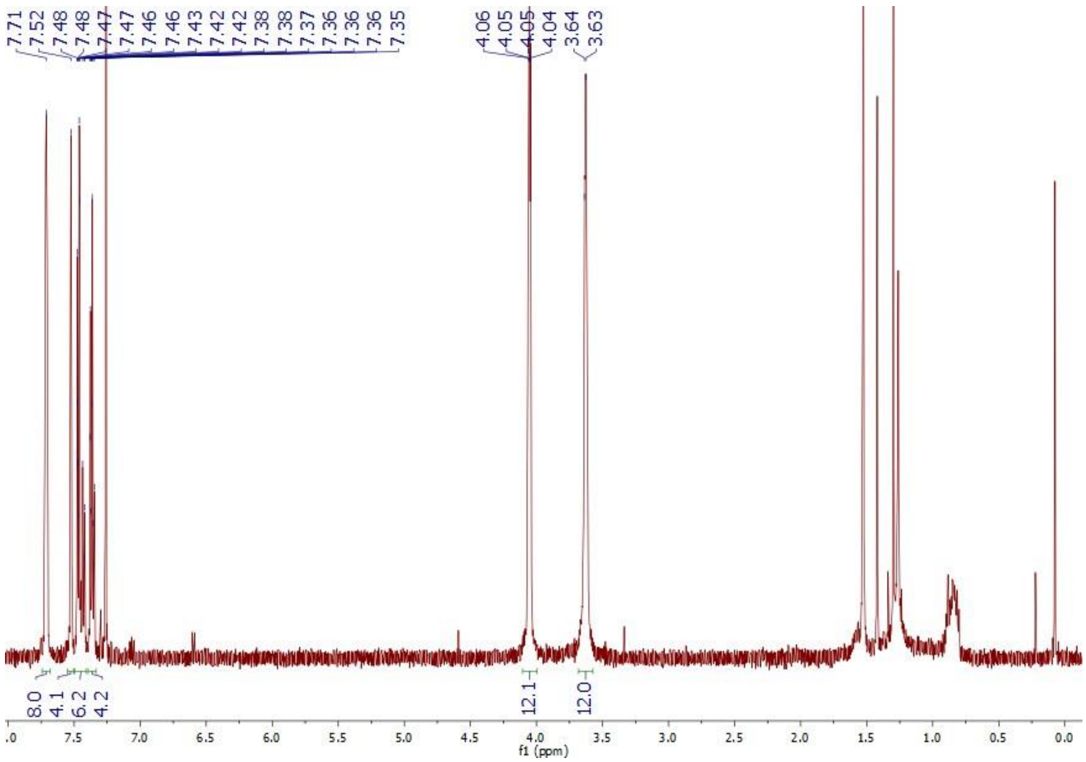

^{13}C NMR spectrum (125 MHz, $CDCl_3$, 293 K):

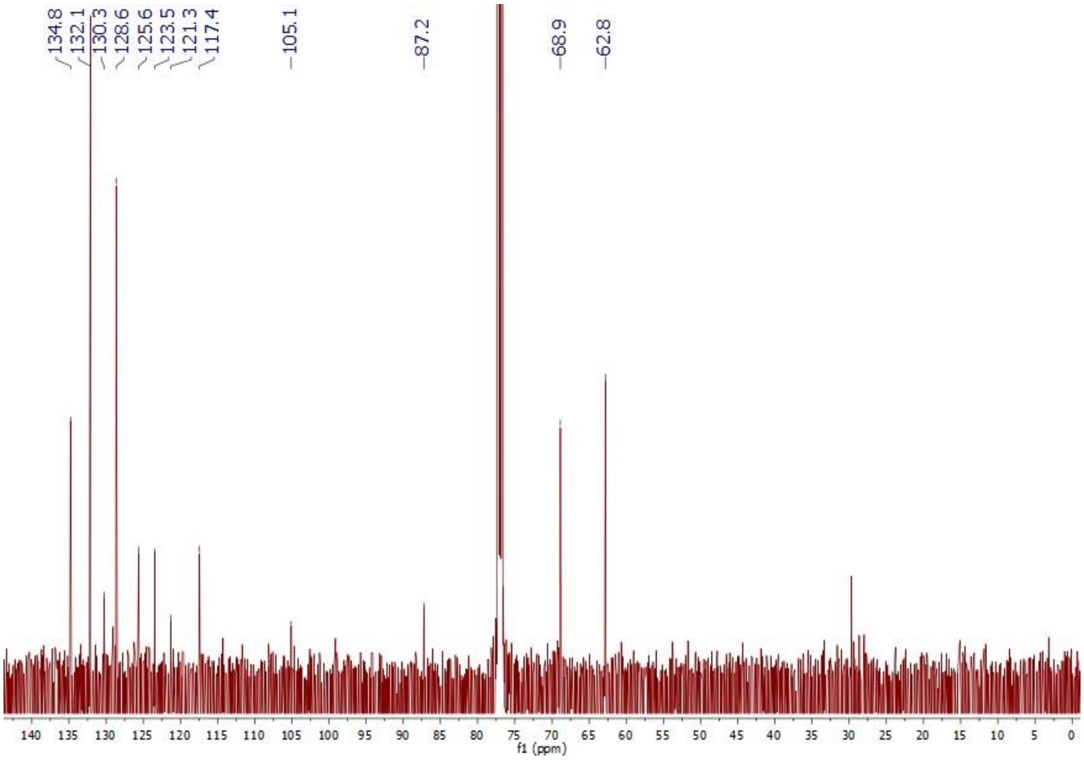

CuAAC "Click" Reaction

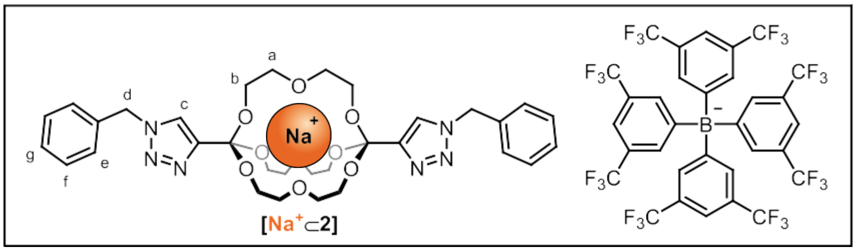

$Cu(MeCN)_4PF_6$ (0.11 µmol, 0.01 equiv., 0.04 mg) and tris[(1-benzyl-1*H*-1,2,3-triazol-4-yl)methyl)]amine (0.11 µmol, 0.01 equiv., 0.06 mg) were dissolved in anhydrous methanol (2 mL). **[Na$^+$⊂*o*-(C≡CH)$_2$-1.1.1]BArF$^-$** (11.0 µmol, 1.0 equiv., 14.0 mg) and benzyl azide (22.0 µmol, 2.0 equiv., 0.5 M in CH_2Cl_2, 44.0 µL) were added. The reaction mixture was stirred at 70 °C for 24 h. The solvent was removed under reduced pressure and the crude product was purified by passing it over a short plug of silica gel. The plug was purged with anhydrous acetonitrile, removal of the solvent gave the product **[Na$^+$⊂2]BArF$^-$** as colourless solid (15.5 mg, 92%).

^1H-NMR (500 MHz, CD_3CN, 293 K): δ (ppm) = 7.84 (s, 2H, c), 7.71 – 7.69 (m, 8H), 7.68 – 7.66 (m, 4H), 7.42 – 7.33 (m, 6H, e-g), 7.33 – 7.27 (m, 4H, e-g), 5.55 (s, 4H, d), 3.92 – 3.85 (m, 12H, b), 3.73 – 3.69 (m, 12H, a).

^{13}C-NMR: (125 MHz, CD_3CN, 293 K): δ (ppm) = 163.2, 162.8, 162.4, 162.0, 145.8, 136.6, 135.7, 130.1, 130.1, 130.0, 130.0, 129.9, 129.8, 129.8, 129.8, 129.8, 129.4, 129.0, 128.7, 126.5, 124.7, 124.4, 122.2, 118.7, 118.7, 118.7, 111.1, 70.7, 64.0, 54.6.

HRMS (ESI$^+$): *m/z* = 675.2742 $[M+Na]^+$ (calcd. 675.2749 for $C_{32}H_{40}N_6NaO_9$).

^{1}H NMR spectrum (500 MHz, CD_3CN, 293 K):

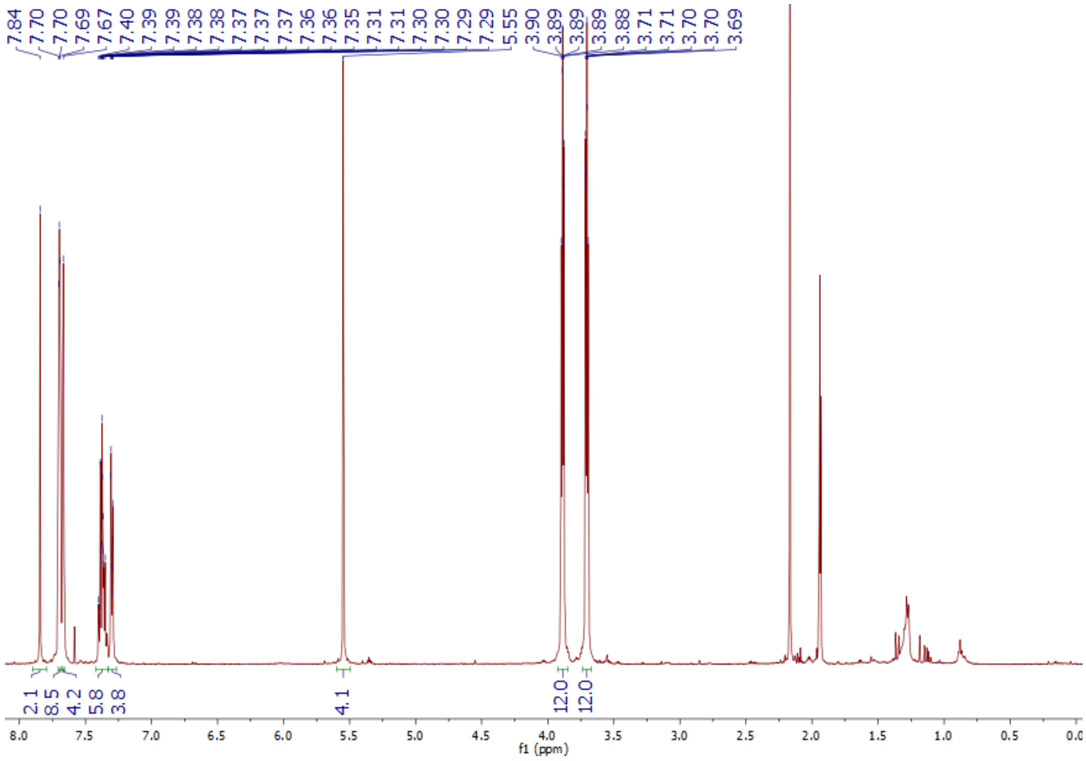

^{13}C NMR spectrum (125 MHz, CD_3CN, 293 K):

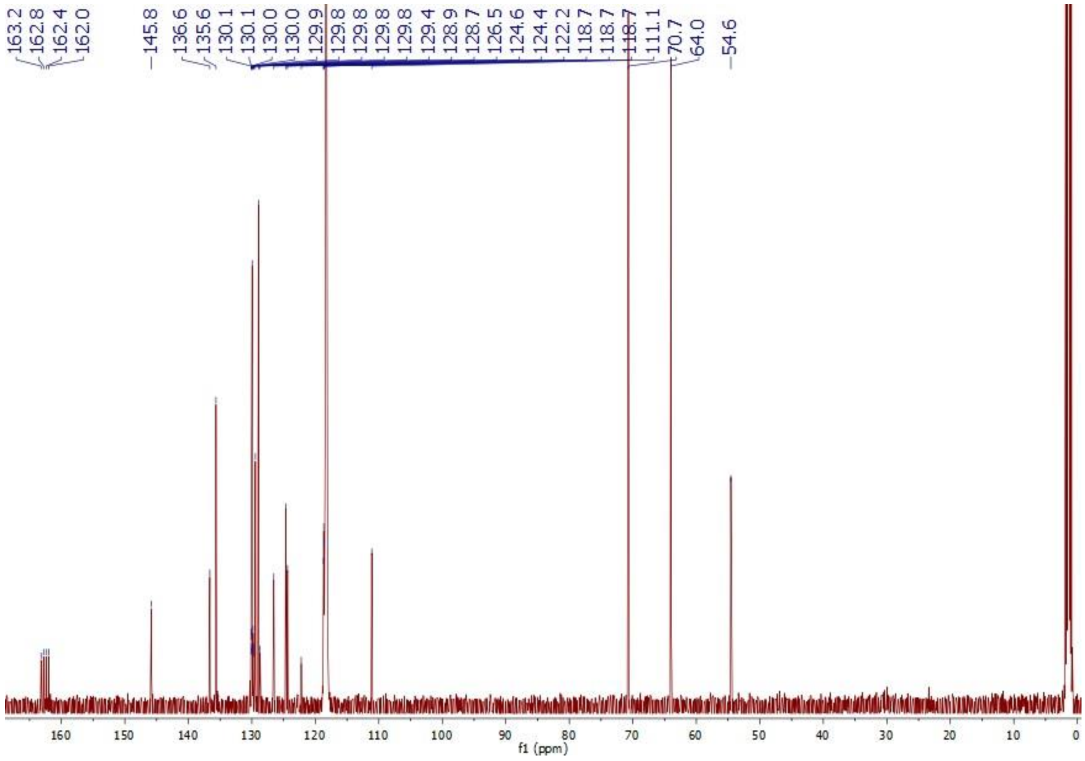

Triazole Methylation

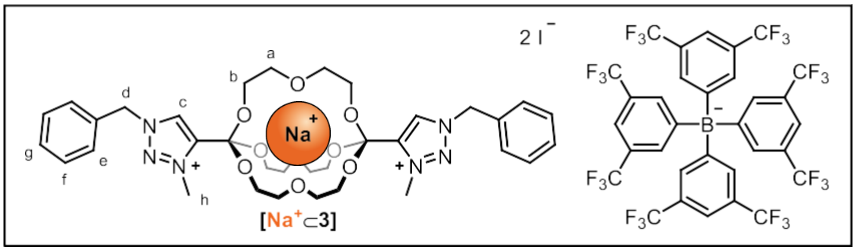

Cryptate **[Na⁺⊂2]BArF⁻** (3.90 µmol, 1.0 equiv., 6 mg) was dissolved in anhydrous acetonitrile (1 mL). Excess iodomethane was added. The reaction mixture was heated to 70 °C for 3 days. The solvent and residual iodomethane were removed under reduced pressure to give the product **[Na⁺⊂3]BArF⁻** in reasonable purity as slightly yellow solid (5.9 mg, 83%).

^{1}H-NMR (500 MHz, CD_3CN, 293 K): δ (ppm) = 8.60 (s, 2H, c), 7.70 – 7.68 (m, 8H), 7.68 – 7.66 (m, 4H), 7.53 – 7.43 (m, 10H, e-g), 5.71 (s, 4H, d), 4.20 (s, 6H, h), 3.88 – 3.83 (m, 12H, b), 3.78 – 3.72 (m, 12H, a).

^{13}C-NMR: (125 MHz, CD_3CN, 293 K): δ(ppm) = 135.6, 132.6, 130.7, 130.5, 130.4, 130.2, 130.0, 129.7, 126.8, 124.1, 119.1, 109.7, 70.5, 64.7, 58.2, 40.8.

HRMS (ESI^{+}): *m/z* = 341.1657 $[M]^{2+}$ (calcd. 341.1658 for $C_{34}H_{46}N_6O_9$).

^{1}H NMR spectrum (500 MHz, CD_3CN, 293 K):

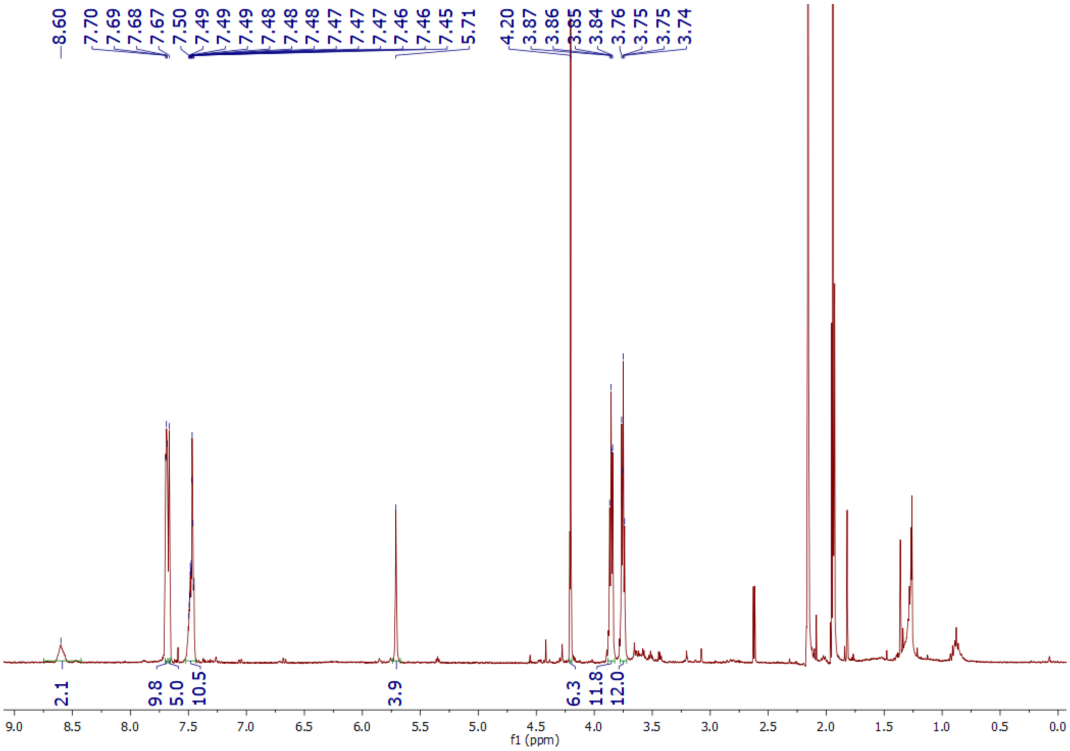

^{13}C NMR spectrum (125 MHz, CD_3CN, 293 K):

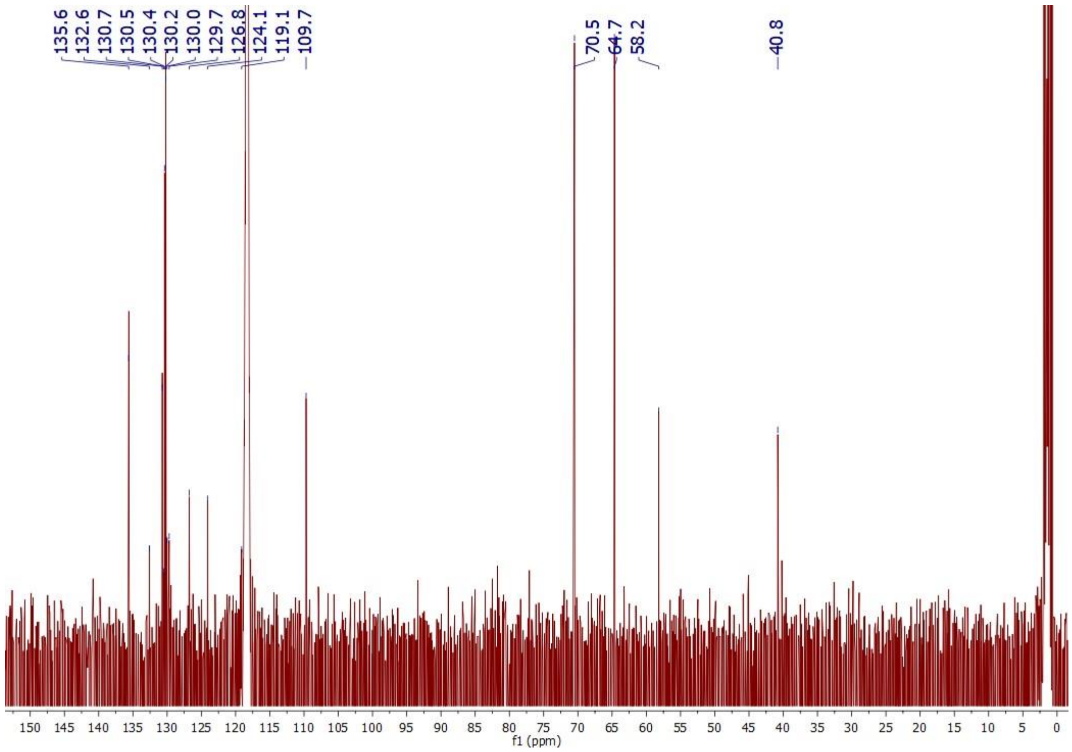

3. Experimental Binding Studies

The NMR titrations were carried out according to general procedure D.

	c [mM]	K_a [M^{-1}]	Fit error [M^{-1}]	avg. K_a [M^{-1}]	u [M^{-1}]	$U_{95\%}$ [M^{-1}]
	5	70	2			
NaBArF	5	60	1	60	5	±20
	10	50	1			

Table S1: Association constants obtained for the titration of *o*-$(H)_2$-1.1.1 with NaBArF in CD_3CN at 298 K. Fit method: Nelder-Mead. Binding model: 1:1.; u: standard uncertainty = $s/\sqrt{\square}$; s: standard deviation; n: number of measurements; U: 95% confidence interval = $t_{(0.05,2)} \times u$; $t_{(\alpha,\ n-1)}$: student-t distribution at a probability α.[8]

	c [mM]	K_a [M^{-1}]	Fit error [M^{-1}]	avg. K_a [M^{-1}]	u [M^{-1}]	$U_{95\%}$ [M^{-1}]
	1	13000	4000			
LiBArF	5	12000	2000	13000	300	±1000
	5	13000	5000			

Table S2: Association constants obtained for the titration of *o*-$(H)_2$-1.1.1 with LiBArF in CD_3CN at 298 K. Fit method: Nelder-Mead. Binding model: 1:1.; u: standard uncertainty = $s/\sqrt{\square}$; s: standard deviation; n: number of measurements; U: 95% confidence interval = $t_{(0.05,2)} \times u$; $t_{(\alpha,\ n-1)}$: student-t distribution at a probability α.[8]

NaBArF 5 mM	http://app.supramolecular.org/bindfit/view/56fafe98-d01f-4442-be8b-0ae35bd2edda
NaBArF 5 mM	http://app.supramolecular.org/bindfit/view/4c4814f0-e502-4836-813a-941e23ab63ec
NaBArF 10 mM	http://app.supramolecular.org/bindfit/view/3cd7bd15-9b97-49c8-8bd8-9ea3ee363ca6
LiBArF 1 mM	http://app.supramolecular.org/bindfit/view/4ea4dc4a-1db3-4639-a809-82d50b77617a
LiBArF 5 mM	http://app.supramolecular.org/bindfit/view/38ef2276-345e-40d6-ace3-c21e7bc07d51
LiBArF 5 mM	http://app.supramolecular.org/bindfit/view/da8bdd85-60e0-48bd-8f66-20f51b131351

Table S3: Links to raw data, calculated fits and statistical information for the titrations.

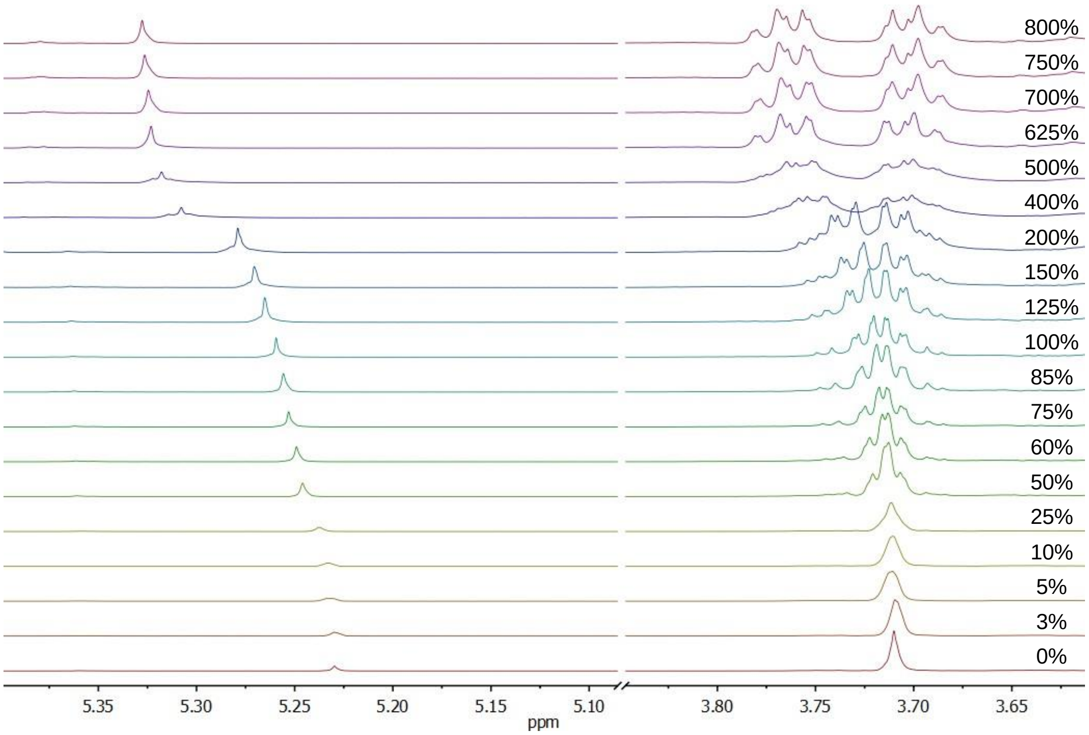

Figure S1: Representative **p**artial ^{1}H NMR (400 MHz, 298 K, CD_3CN) stack plot for a titration of *o*-$(H)_2$-1.1.1 (5 mM) with **NaBArF** from 0-800%.

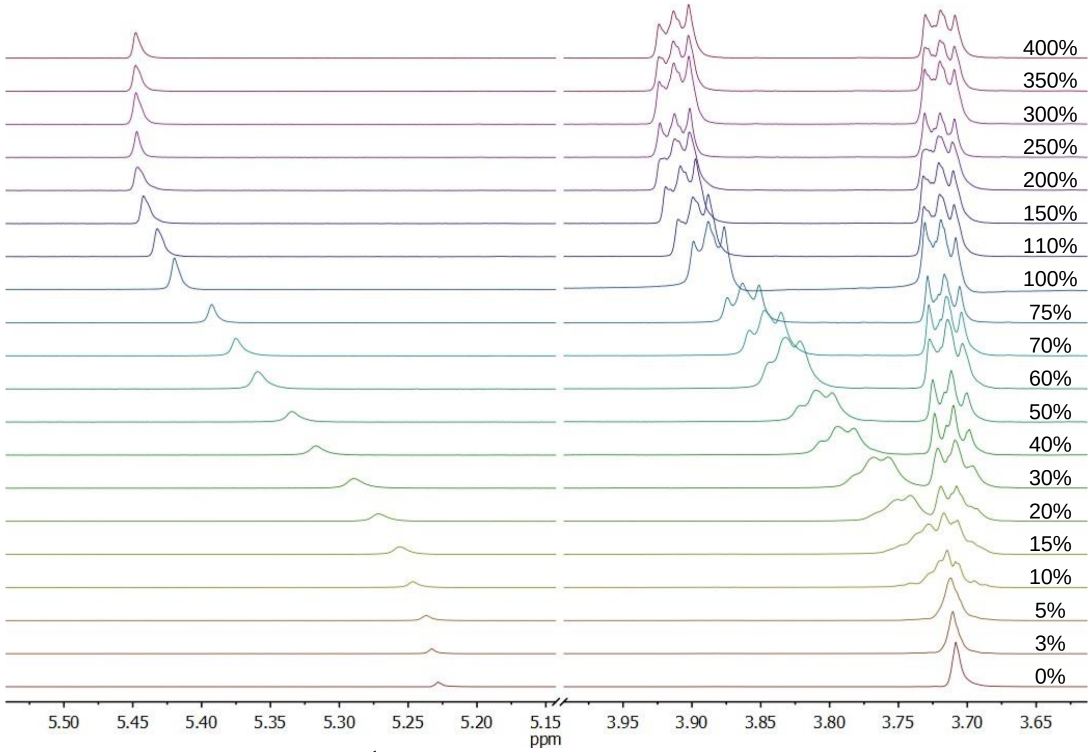

Figure S2: Representative partial ^{1}H NMR (400 MHz, 298 K, CD_3CN) stack plot for a titration of *o*-$(H)_2$-1.1.1 (5 mM) with **LiBArF** from 0-400%.

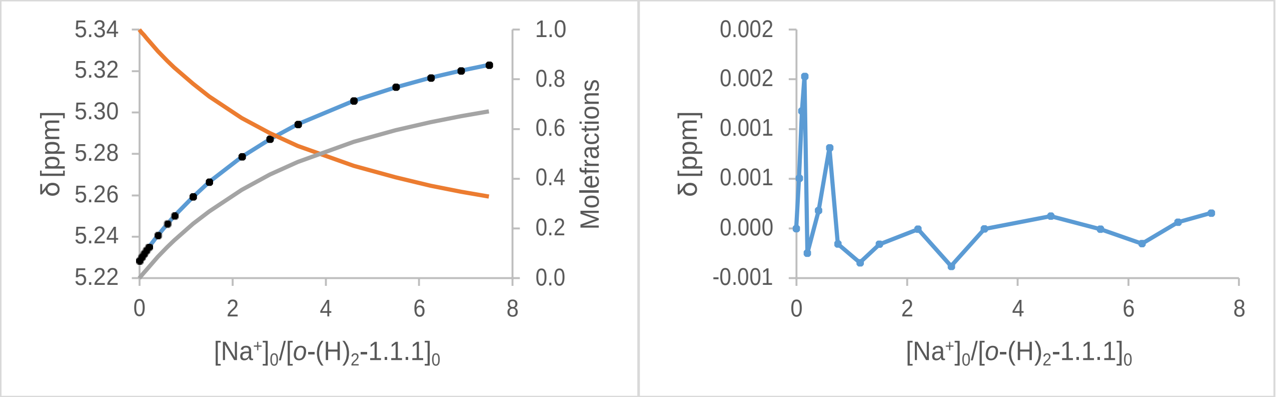

Figure S3: Left: Binding isotherm and species concentration plot for titration of *o*-(H)$_2$-1.1.1 (5 mM) with **NaBArF** in CD_3CN at 298 K. Black dots: Experimental points; Blue line: Fit according to 1:1 model; orange und grey lines: mole fractions of corresponding species. Right: Residual plot.

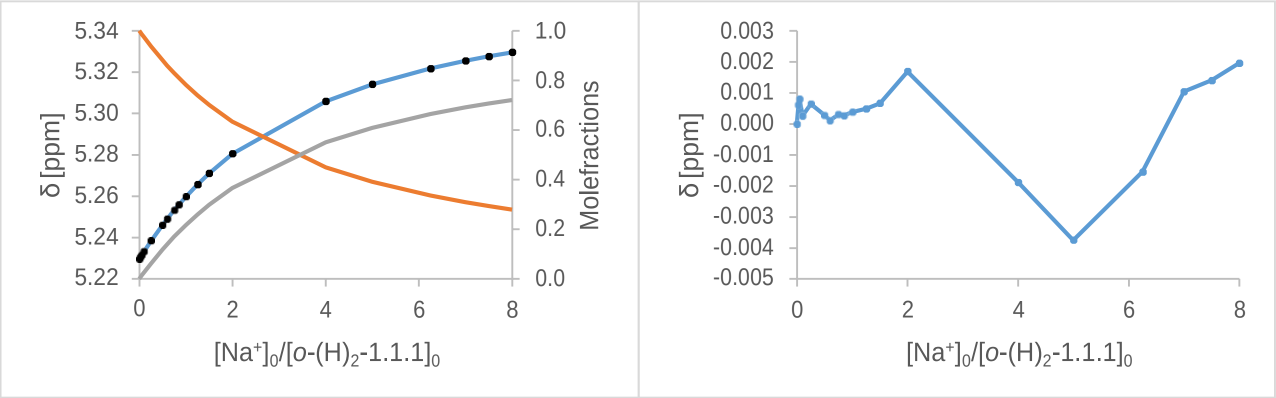

Figure S4: Left: Binding isotherm and species concentration plot for titration of *o*-(H)$_2$-1.1.1 (5 mM) with **NaBArF** in CD_3CN at 298 K. Black dots: Experimental points; Blue line: Fit according to 1:1 model; orange und grey lines: mole fractions of corresponding species. Right: Residual plot.

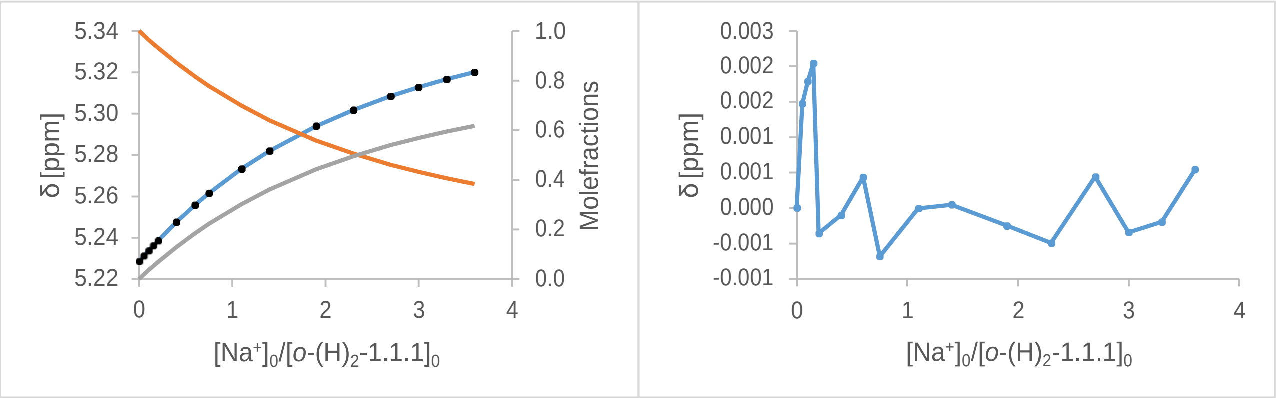

Figure S5: Left: Binding isotherm and species concentration plot for titration of *o*-(H)$_2$-1.1.1 (10 mM) with **NaBArF** in CD_3CN at 298 K. Black dots: Experimental points; Blue line: Fit according to 1:1 model; orange und grey lines: mole fractions of corresponding species. Right: Residual plot.

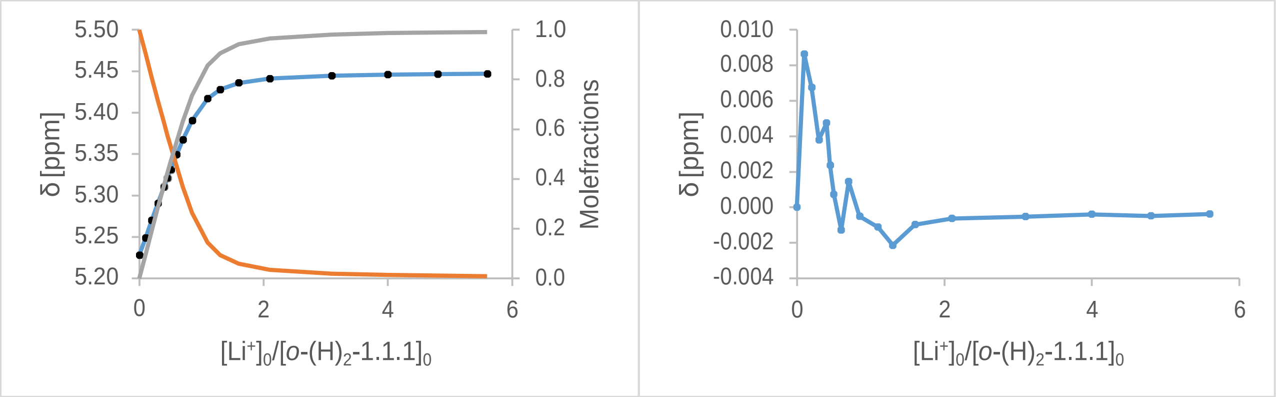

Figure S6: Left: Binding isotherm and species concentration plot for titration of *o*-$(H)_2$-1.1.1 (1 mM) with **LiBArF** in CD_3CN at 298 K. Black dots: Experimental points; Blue line: Fit according to 1:1 model; orange und grey lines: mole fractions of corresponding species. Right: Residual plot.

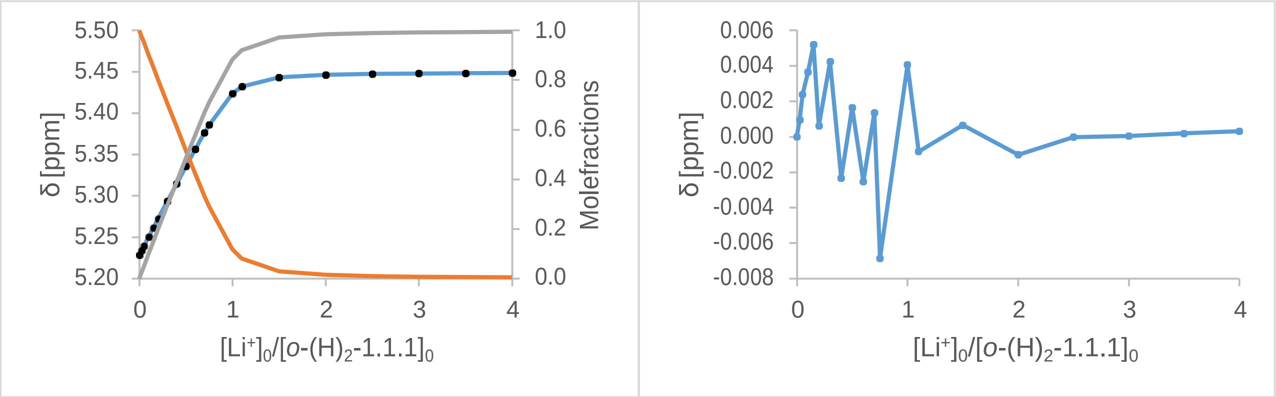

Figure S7: Left: Binding isotherm and species concentration plot for titration of *o*-$(H)_2$-1.1.1 (5 mM) with **LiBArF** in CD_3CN at 298 K. Black dots: Experimental points; Blue line: Fit according to 1:1 model; orange und grey lines: mole fractions of corresponding species. Right: Residual plot.

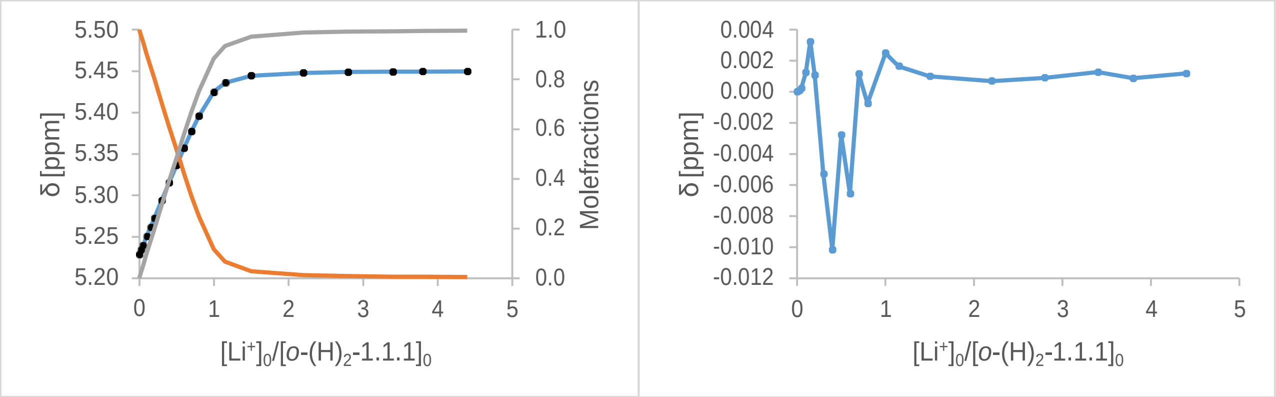

Figure S8: Left: Binding isotherm and species concentration plot for titration of *o*-$(H)_2$-1.1.1 (5 mM) with **LiBArF** in CD_3CN at 298 K. Black dots: Experimental points; Blue line: Fit according to 1:1 model; orange und grey lines: mole fractions of corresponding species. Right: Residual plot.

4. Monitoring of orthoester exchange and hydrolysis

Exchange reactions with ethanol

Reaction scheme:

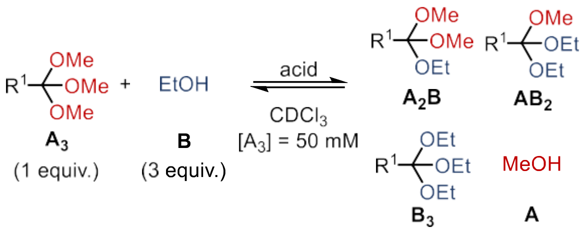

The orthoester exchange reactions were carried out according to general procedure B.

Summary of results:

Acid	R^1	*t* [min]	Ratio A_3:A_2B:AB_2:B_3	Taft
0.01% TFA	-nC_4H_9	10	n.d.	-0.13
	-CH_3	280	26:44:24:6	0.00
0.1% TFA	-$CH_2C_6H_5$	650	24:41:29:6	0.22
1% TFA	-H	40	19:43:31:7	0.49
	-C_6H_5	50	23:42:27:7	0.60
	-C≡C-$Si(CH_3)_3$	90	22:41:29:7	-
	-triazole[a]	130	n.d.	-
	-CH_2Cl	640	16:42:32:10	1.05
10% TFA	-C≡CH	300	32:35:26:6	2.18
50% TfOH	-CCl_3	330	n.d.	2.65
	-triazolium[b]	490	16:39:33:11	-
100% TfOH	-CF_3	>1000	n.d.	2.61
	-CN	Decomposition of CN-group		3.30

Reactivity ↑

Table S4: Kinetics and scope of orthoester exchange reaction and comparison to Taft parameter.[9] *t*: equilibration time, defined as point when 99% conversion to plateau-level of methanol A were exceeded for the first time; n.d.: not detectable via ^{1}H-NMR spectroscopy. [a] 1-benzyl-4-(trimethoxymethyl)-1*H*-1,2,3-triazole. [b] 1-benzyl-3-methyl-4-(trimethoxymethyl)-1*H*-1,2,3-triazol-3-ium.

R^1	Acid	*t* [min]	avg. *t* [min]	*u* [min]	$U_{95\%}$ [min]
-H	1% TFA	37			
-H	1% TFA	38	38	0.5	±2
-H	1% TFA	39			

Table S5: Representative calculation of confidence interval for triplicate orthoester exchange reaction of trimethyl orthoformate with ethanol. *u*: standard uncertainty = $s/\sqrt{□}$; *s*: standard deviation; *n*: number of measurements; *U*: 95% confidence interval = $t_{(0.05,2)} \times u$; $t_{(\alpha,\, n-1)}$: student-t distribution at a probability α.[8]

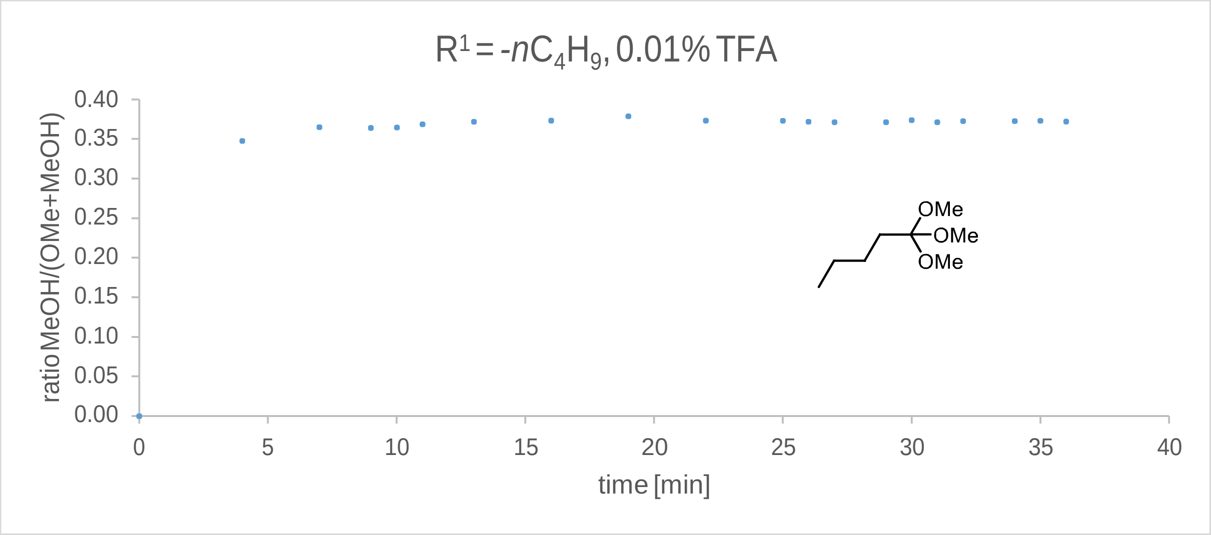

Graph S1: Time-dependent methanol development of the exchange reaction of trimethyl orthovalerate with ethanol.

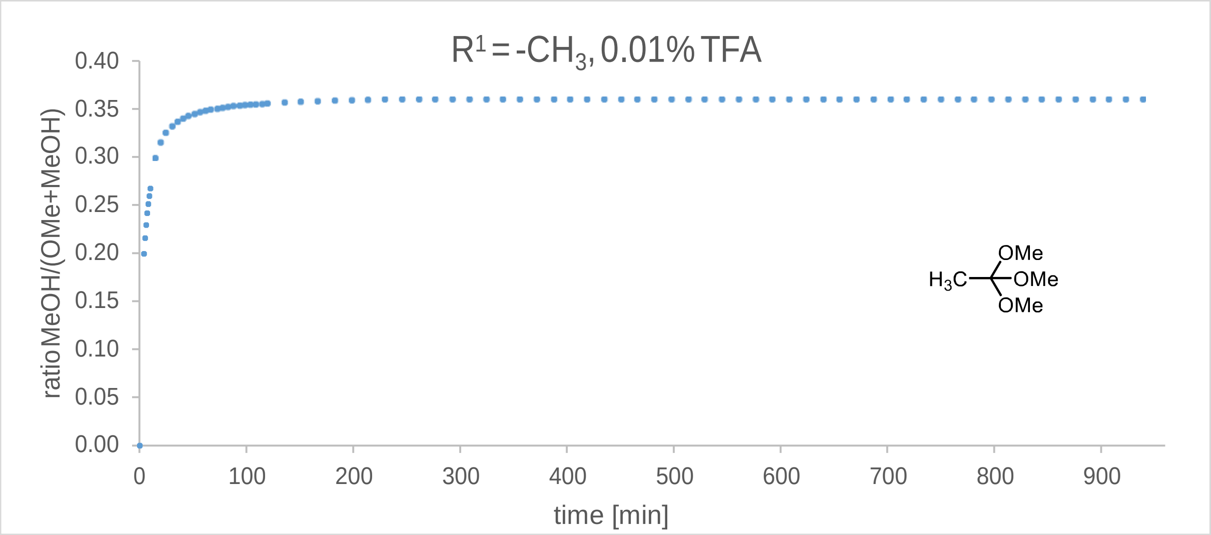

Graph S2: Time-dependent methanol development of the exchange reaction of trimethyl orthoacetate with ethanol.

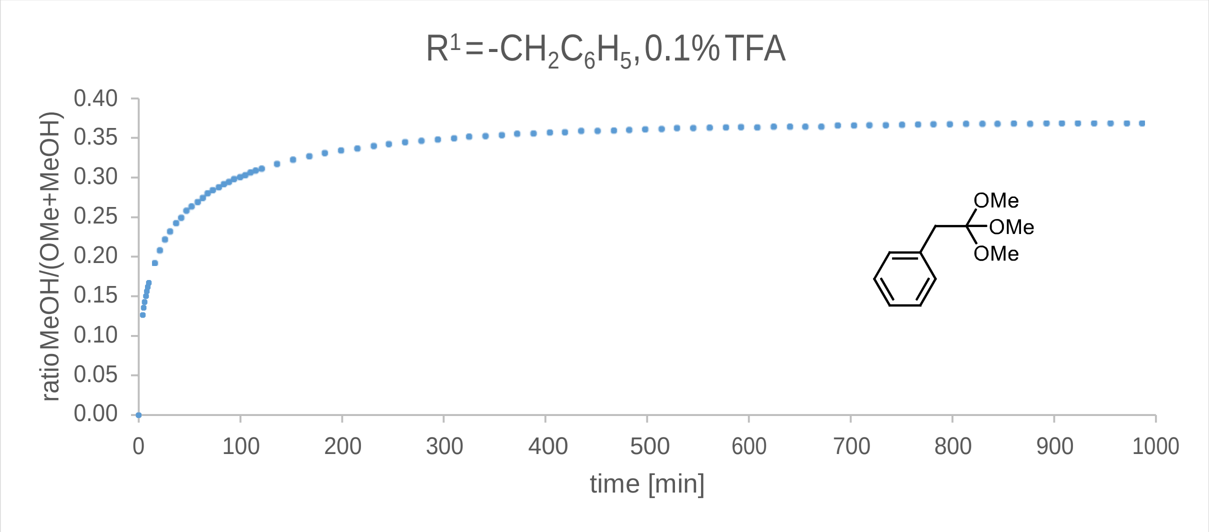

Graph S3: Time-dependent methanol development of the exchange reaction of (2,2,2-trimethoxyethyl)benzene with ethanol.

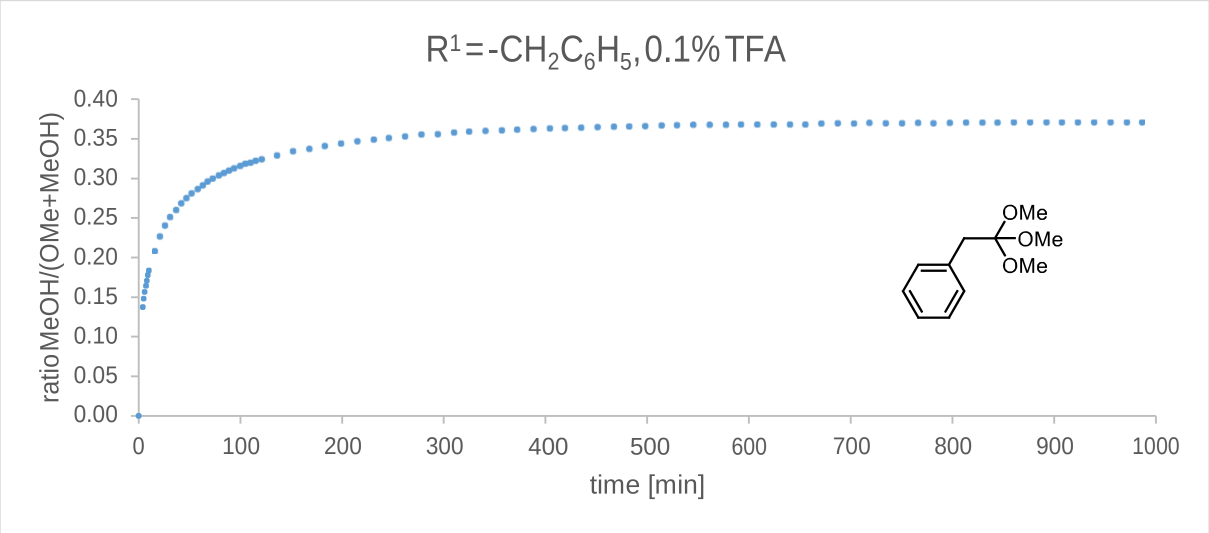

Graph S4: Reproduced time-dependent methanol development of the exchange reaction of (2,2,2-trimethoxyethyl)benzene with ethanol.

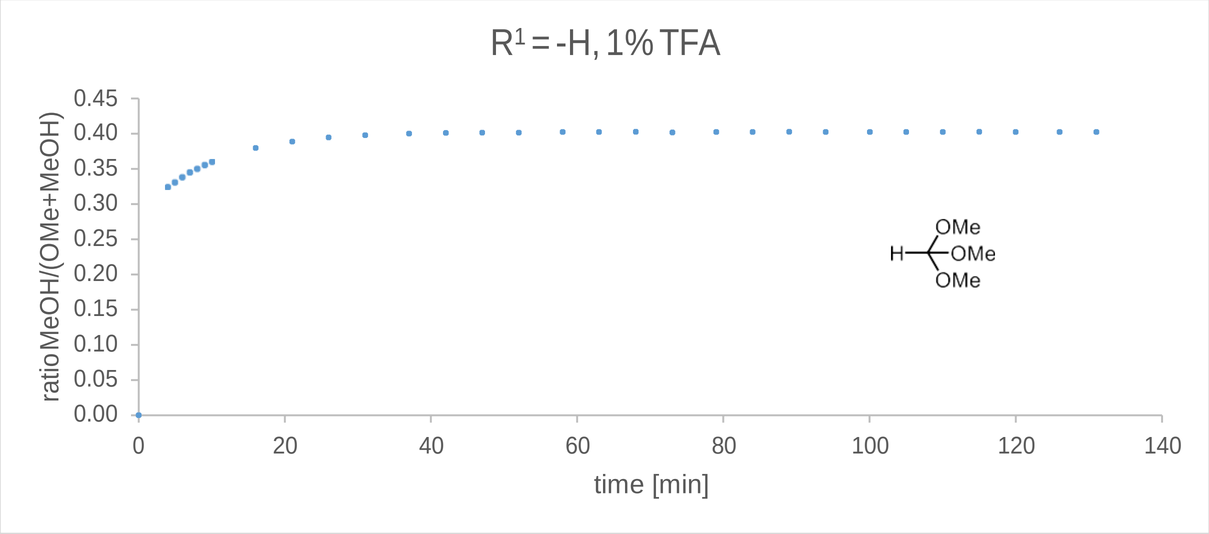

Graph S5: Time-dependent methanol development of the exchange reaction of trimethyl orthoformate with ethanol.

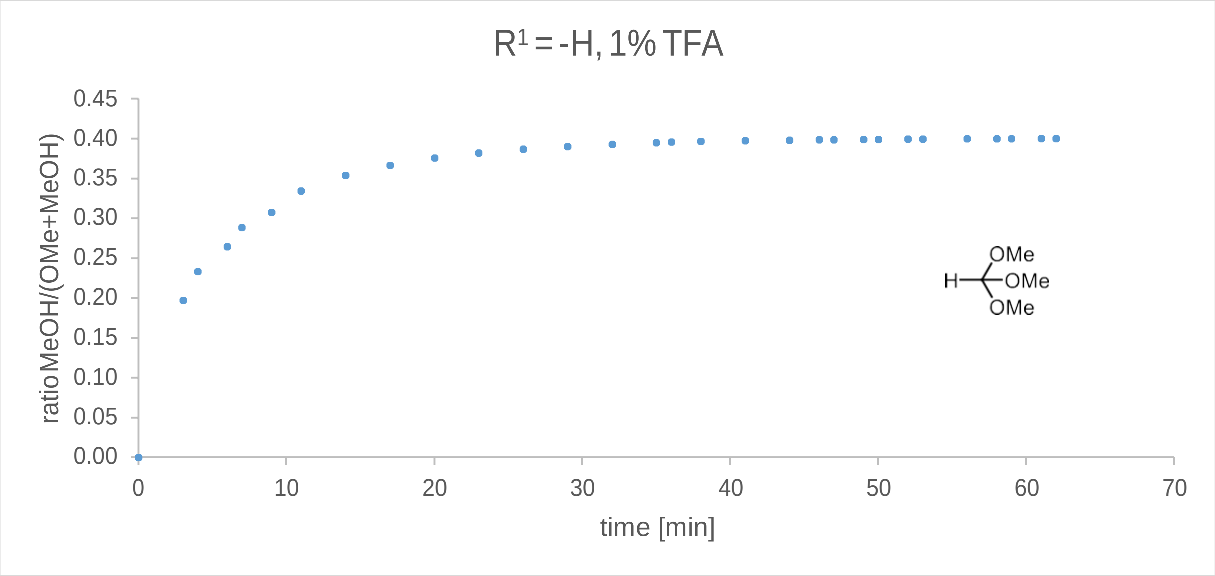

Graph S6: Reproduced time-dependent methanol development of the exchange reaction of trimethyl orthoformate with ethanol.

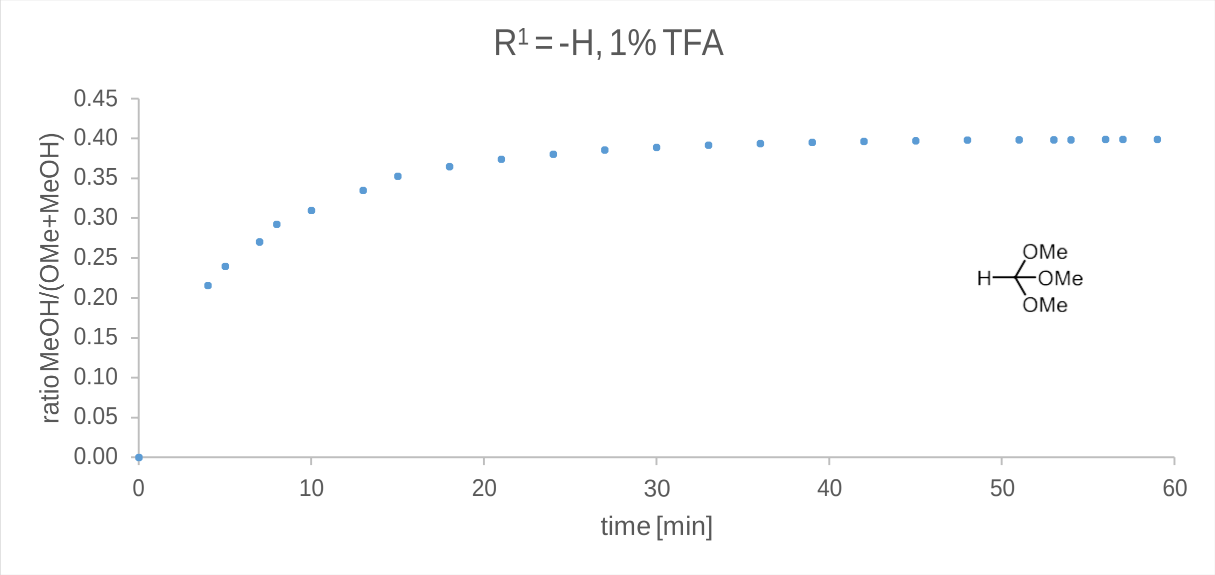

Graph S7: Reproduced time-dependent methanol development of the exchange reaction of trimethyl orthoformate with ethanol.

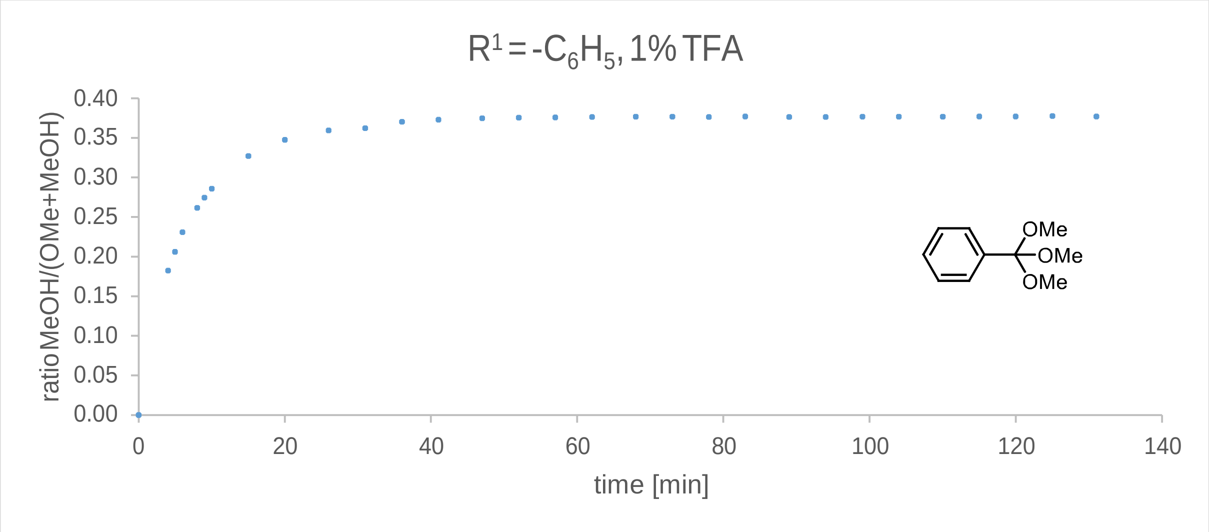

Graph S8: Time-dependent methanol development of the exchange reaction of trimethyl orthobenzoate with ethanol.

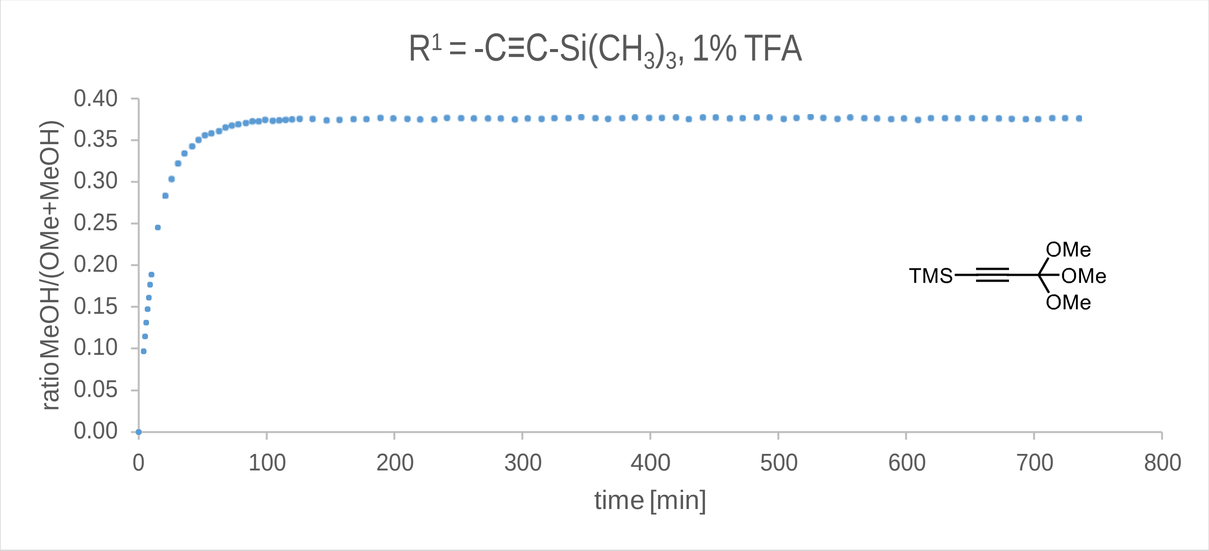

Graph S9: Time-dependent methanol development of the exchange reaction of trimethyl(3,3,3-trimethoxyprop-1-yn-1-yl)silane with ethanol.

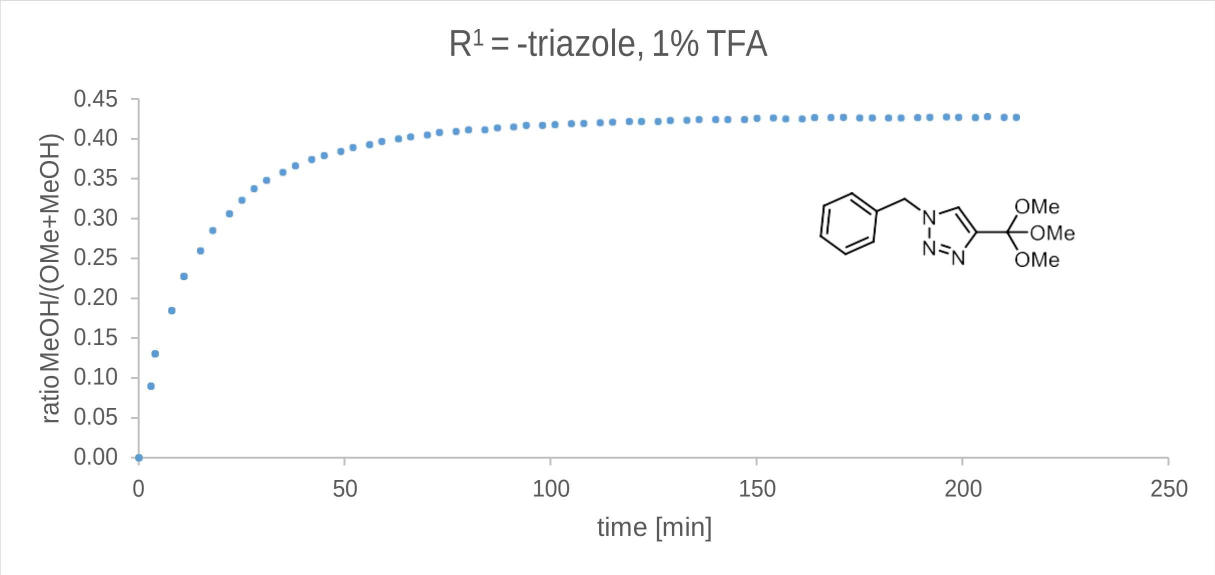

Graph S10: Time-dependent methanol development of the exchange reaction of 1-benzyl-4-(trimethoxymethyl)-1*H*-1,2,3-triazole with ethanol.

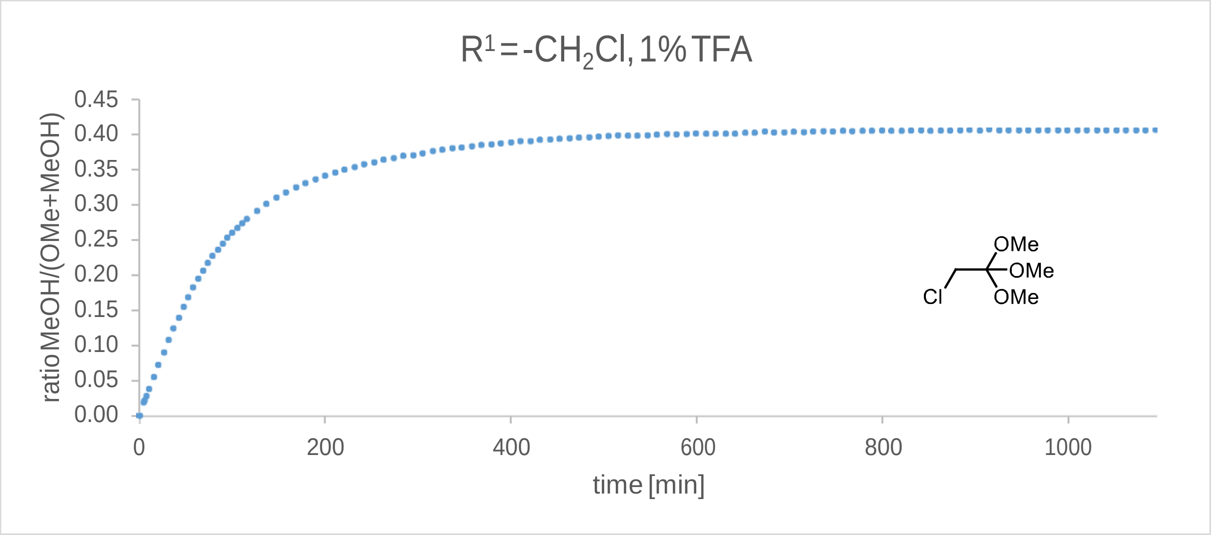

Graph S11: Time-dependent methanol development of the exchange reaction of 2-chloro-1,1,1-trimethoxyethane with ethanol.

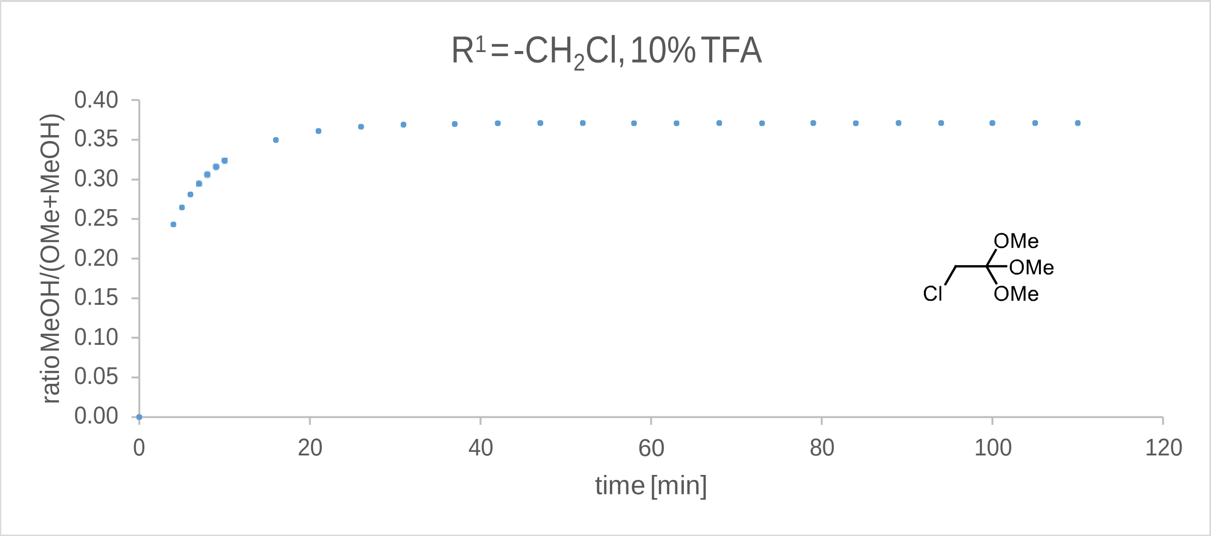

Graph S12: Time-dependent methanol development of the exchange reaction of 2-chloro-1,1,1-trimethoxyethane with ethanol.

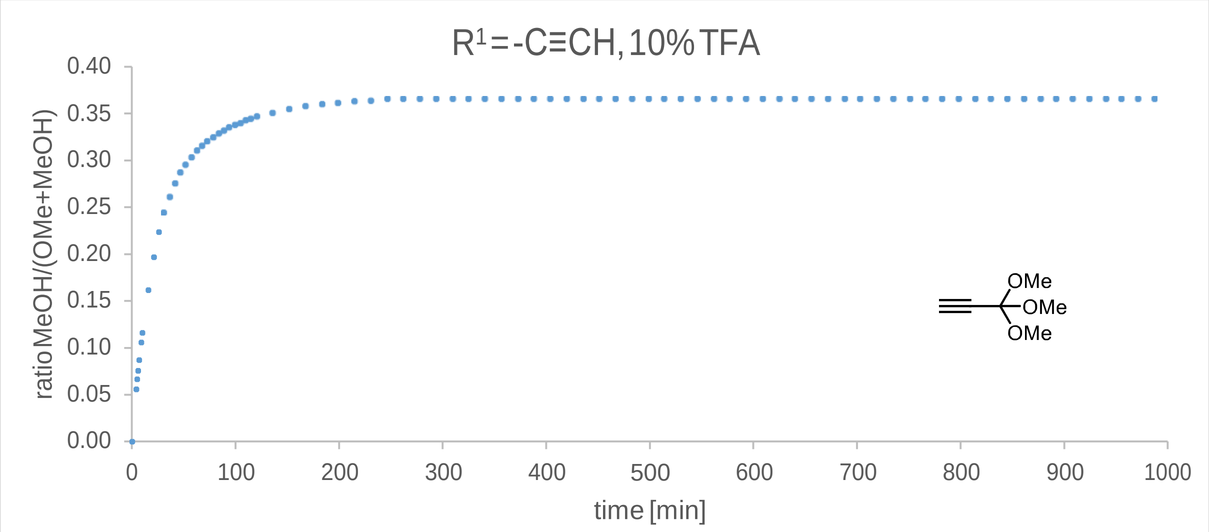

Graph S13: Time-dependent methanol development of the exchange reaction of 3,3,3-trimethoxyprop-1-yne with ethanol.

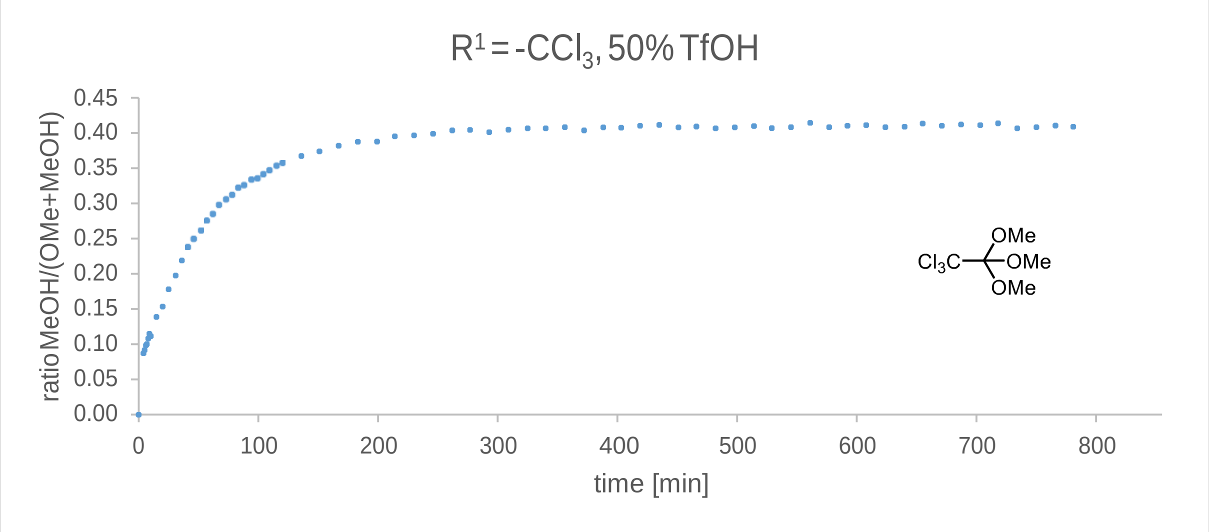

Graph S14: Time-dependent methanol development of the exchange reaction of 1,1,1-trichloro-2,2,2-trimethoxyethane with ethanol.

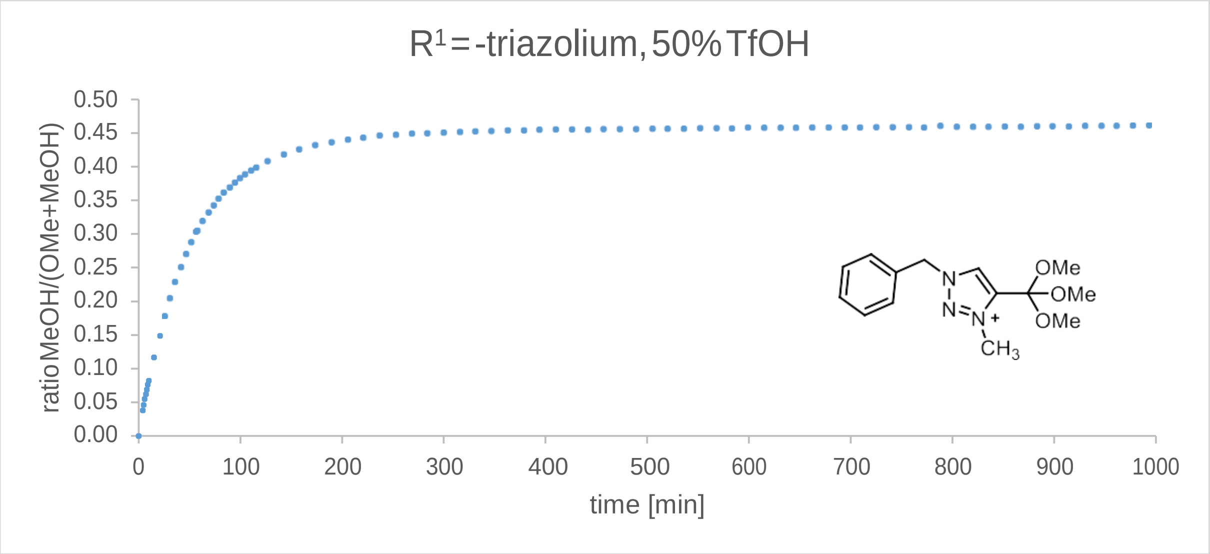

Graph S15: Time-dependent methanol development of the exchange reaction of 1-benzyl-3-methyl-4-(trimethoxymethyl)-1*H*-1,2,3-triazol-3-ium with ethanol.

Product distribution plots

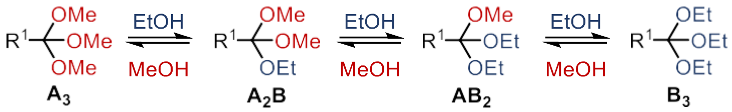

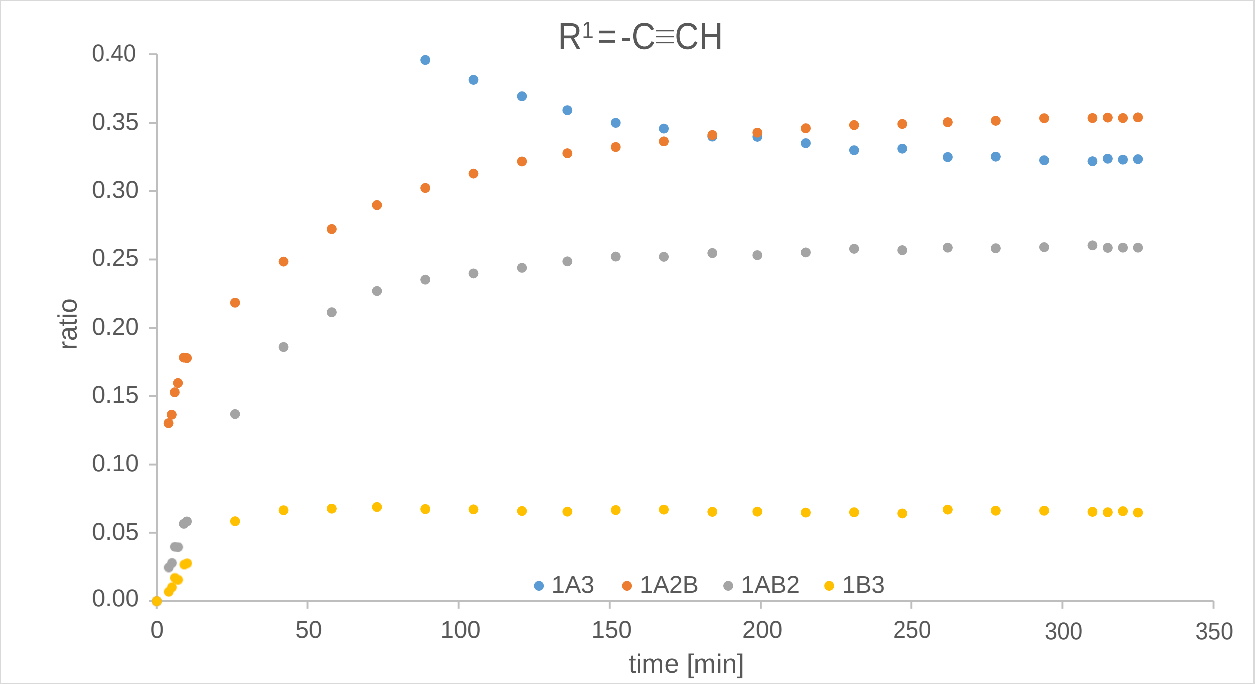

Graph S16: Time-dependent product distribution plot of the exchange reaction of 3,3,3-trimethoxyprop-1-yne with ethanol.

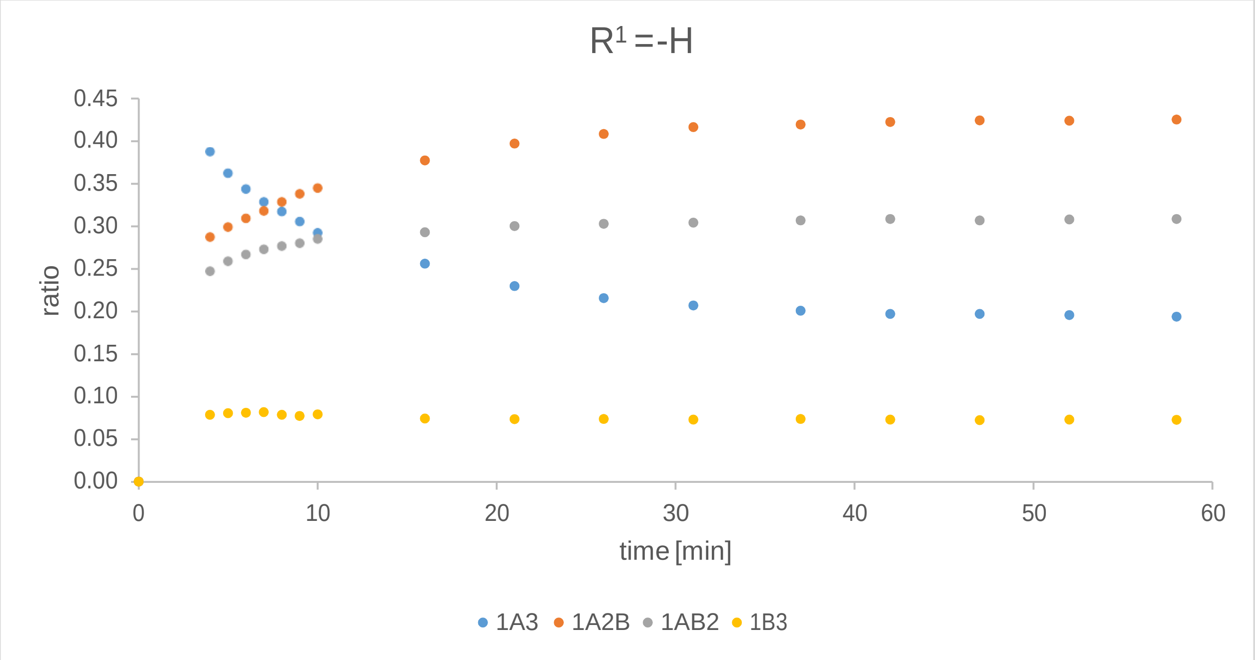

Graph S17: Time-dependent product distribution plot of the exchange reaction of trimethyl orthoformate with ethanol.

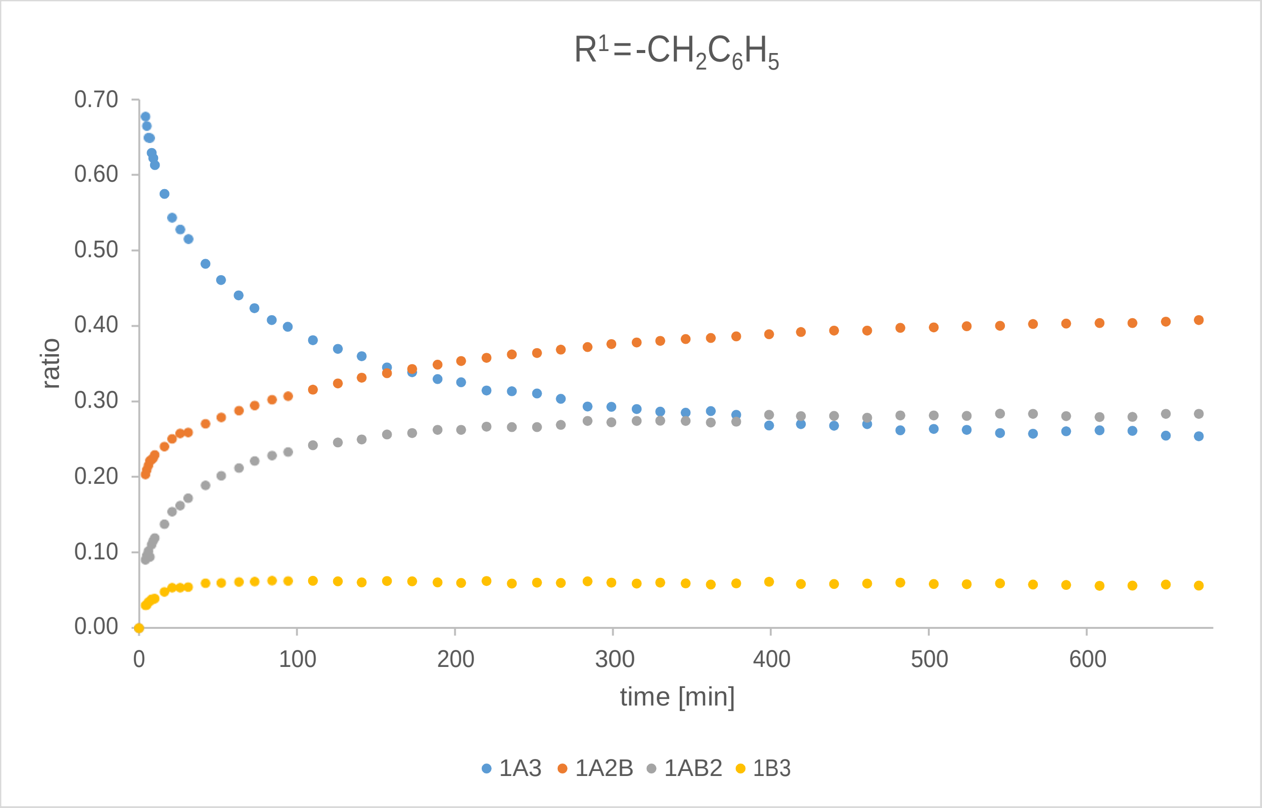

Graph S18: Time-dependent product distribution plot of the exchange reaction of (2,2,2-trimethoxyethyl)benzene with ethanol.

Influence of R^3 on equilibration time and product distribution

Reaction scheme:

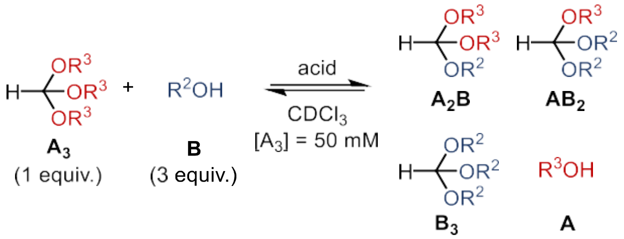

The orthoester exchange reactions were carried out according to general procedure B.

Acid	R^2	R^3	*t* [min]	Ratio (A_3:A_2B:AB_2:B_3)	Taft R^2
1% TFA	$-C_2H_5$	$-CH_3$	40	19:43:31:7	-0.10
1% TFA	$-CH_3$	$-C_2H_5$	40	6:29:44:21	0.00
10% TFA	$-CH_2CH_2Cl$	$-CH_3$	30	40:44:15:1	0.38

Table S6: Influence of R^3 on orthoester exchange reaction and comparison to Taft parameter.[9] *t*: equilibration time, defined as point when 99% conversion to plateau-level of methanol A were exceeded for the first time.

Conclusion: The results indicate that both the kinetics (equilibration time) and the thermodynamics (product ratio) do not change significantly, when substituting either a methyl for an ethyl group and vice versa (Graphs S5 and S16). When looking at the time-dependent product distribution, in both cases the thermodynamically least favoured product (H-C(OC_2H_5)$_3$) reaches its plateau level first (**B_3** in Graph S14 and **A_3** in Graph S17). The same time-dependent product distributions were observed for R^1 = -C≡CH and R^1 = $-CH_2C_6H_5$ (Graphs S13 and S15). The sterically more demanding ethyl groups make R^1-C(OC_2H_5)$_3$ the thermodynamically least favoured products, but **their more electron-donating effect stabilizes the intermediate oxonium ion**. In fact, when starting from trimethyl orthoesters (R^2 = $-C_2H_5$ and R^3 = $-CH_3$) the intermediate leading to the formation of **B_3** is the most stable one, thus leading to faster conversion from **AB_2** to **B_3** than from **A_2B** to **AB_2**. For R^2 = $-CH_3$ and R^3 = $-C_2H_5$, the oxonium ion formed during the reaction from **A_3** to **A_2B** is more stable than from **A_2B** to **AB_2**, which gives an explanation for the observed time-dependent product distribution graphs.

To support this hypothesis, an exchange reaction of trimethyl orthoformate with 2-chloroethanol was carried out (Graph S18, R^2 = $-CH_2CH_2Cl$, R^3 = $-CH_3$). As expected, the reaction proceeds slower and the product distribution differs significantly from previously carried out exchange reactions (Table S4). At equilibrium, only 1 mol% of **B_3** is formed and the thermodynamically favoured products are **A_3** and **A_2B**, which can be explained by the electron-withdrawing effect of the CH_2CH_2Cl-group. Furthermore, **the 2-chloroethanol group destabilizes the intermediate oxonium ions (see Taft value, Table S5), which leads to a markedly different product development profile** (Graph S19). In this case the thermodynamically least favoured product reaches its equilibrium concentration last, because the corresponding intermediate is the least stable.

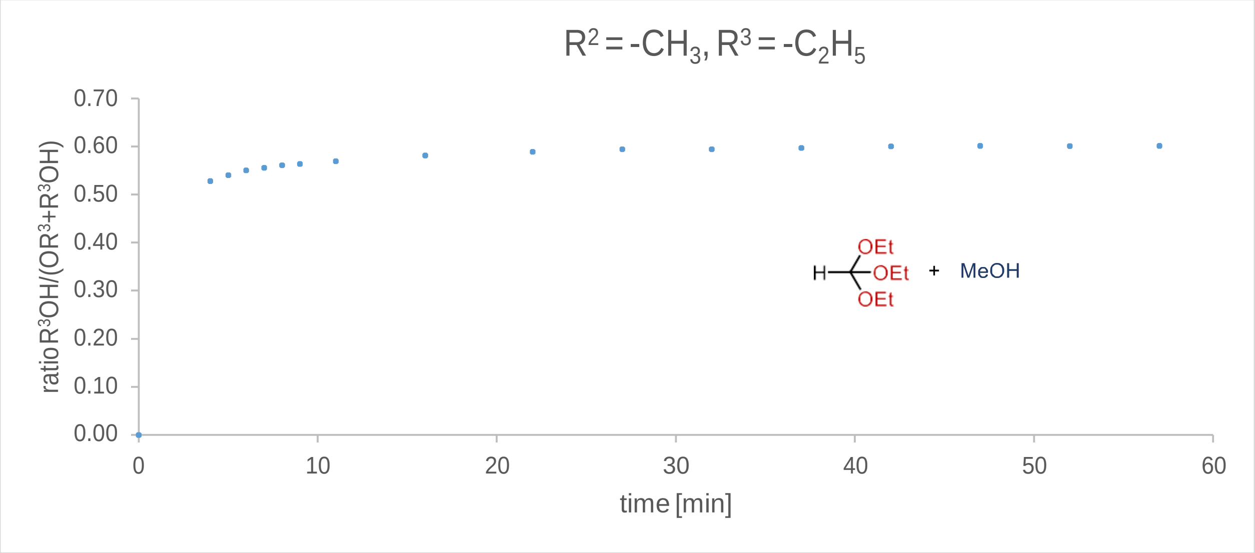

Graph S19: Time-dependent ethanol development of the exchange reaction of triethyl orthoformate with methanol.

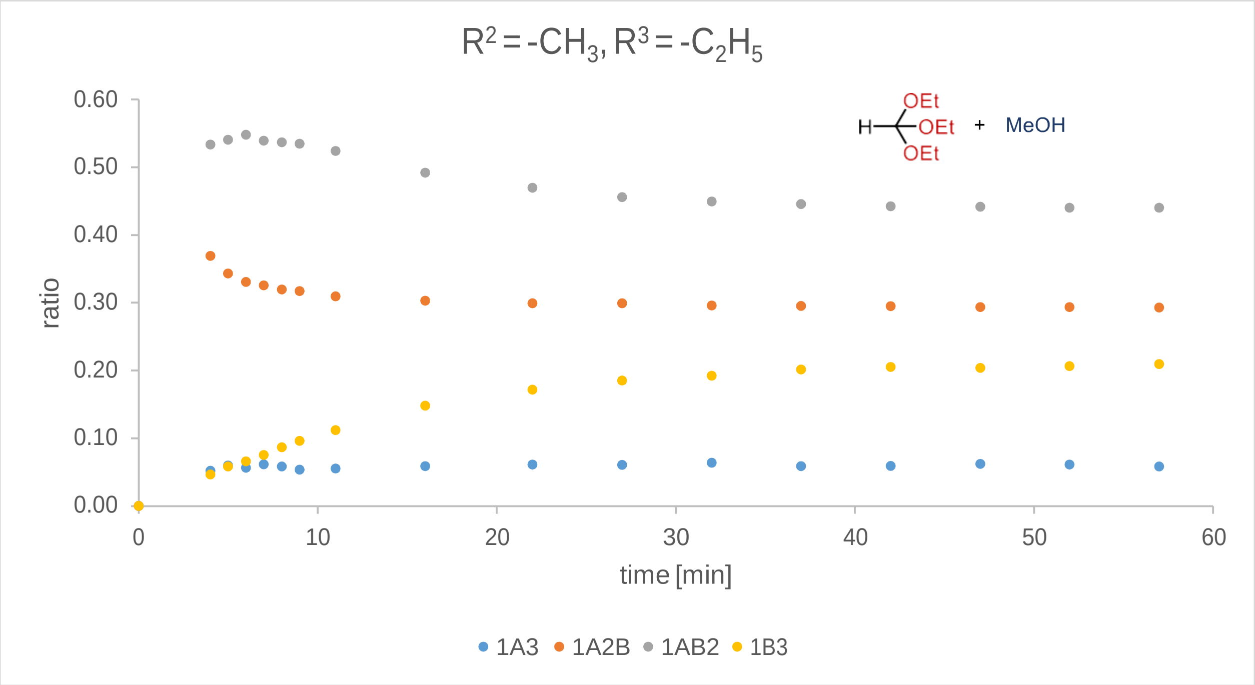

Graph S20: Time-dependent product distribution plot of the exchange reaction of triethyl orthoformate with methanol.

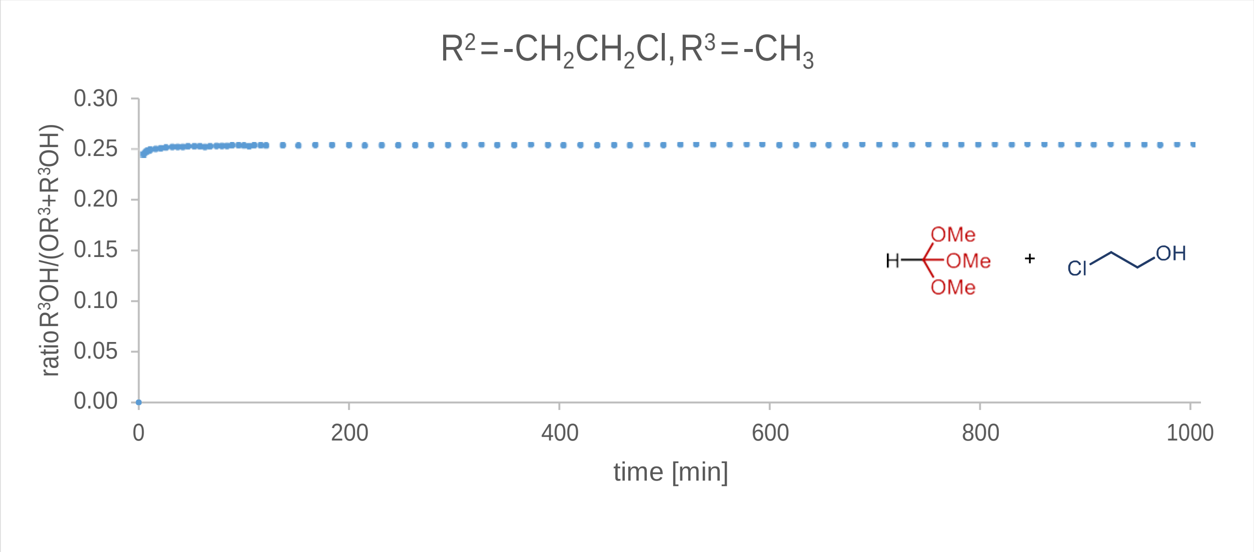

Graph S21: Time-dependent methanol development of the exchange reaction of trimethyl orthoformate with 2-chloroethanol.

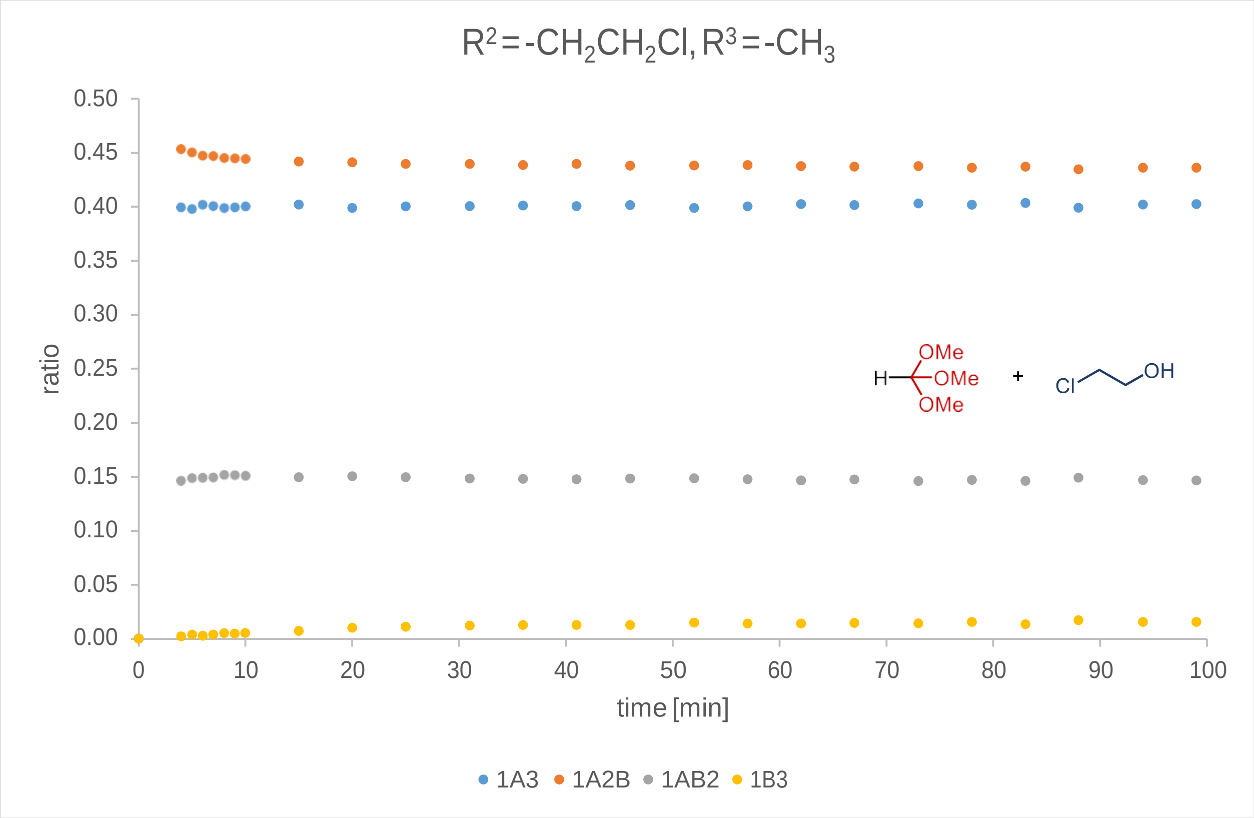

Graph S22: Time-dependent product distribution plot of the exchange reaction of trimethyl orthoformate with 2-chloroethanol.

Simulation of product distributions

All simulations were performed using the free stochastic kinetics simulator Kinetiscope™ (version 1.1.743x64).[10] Rate constants k were estimated using experimental equilibrium constants K, derived from product ratios (Table S6). Simulations were performed for constant volume and pressure at 298.15 K.

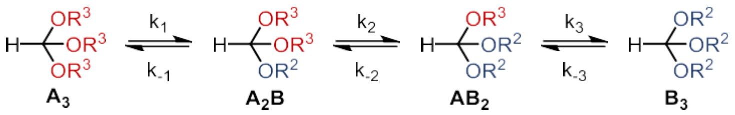

k_1 [min^{-1}]	k_{-1} [min^{-1}]	k_2 [min^{-1}]	K_{-2} [min^{-1}]	k_3 [min^{-1}]	k_{-3} [min^{-1}]
0.10	0.05	1.00	1.50	10.00	49.18

Table S7: Estimated k values used for the simulation of the exchange reaction of trimethyl orthoformate with ethanol.

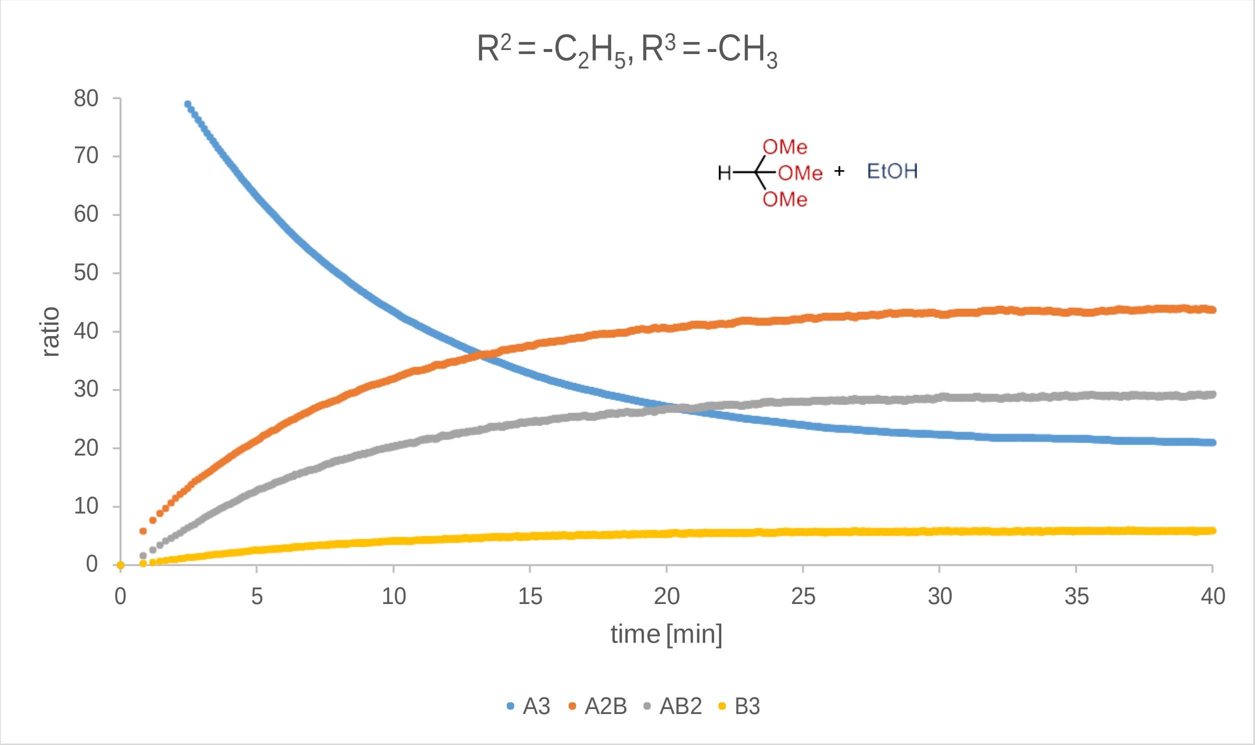

Graph S23: Simulated time-dependent product distribution plot of the exchange reaction of trimethyl orthoformate with ethanol.

k_1 [min^{-1}]	k_{-1} [min^{-1}]	k_2 [min^{-1}]	k_{-2} [min^{-1}]	k_3 [min^{-1}]	k_{-3} [min^{-1}]
49.18	10.00	1.50	1.00	0.05	0.10

Table S8: Estimated k values used for the simulation of the exchange reaction of triethyl orthoformate with methanol.

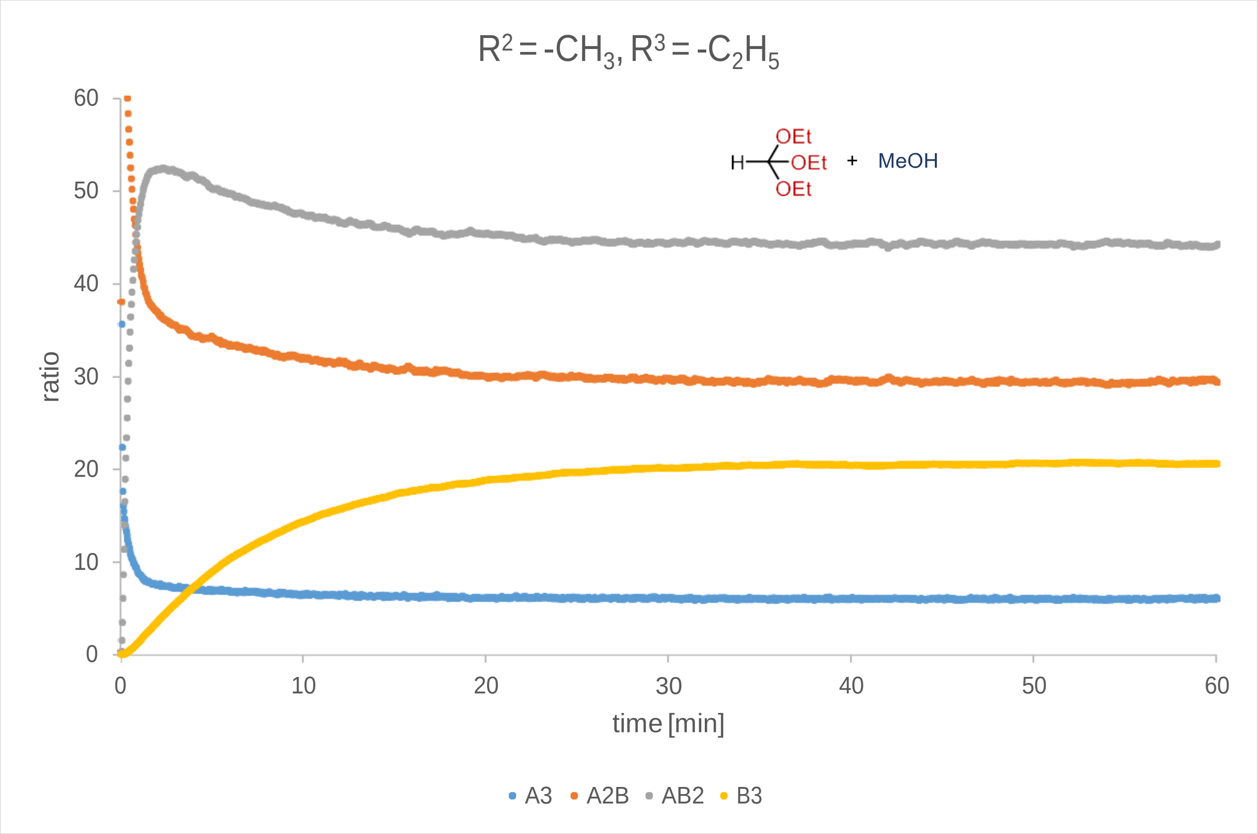

Graph S24: Simulated time-dependent product distribution plot of the exchange reaction of triethyl orthoformate with methanol**.**

k_1 [min^{-1}]	k_{-1} [min^{-1}]	k_2 [min^{-1}]	k_{-2} [min^{-1}]	k_3 [min^{-1}]	k_{-3} [min^{-1}]
5.43	5.00	0.33	1.00	0.01	0.10

Table S9: Estimated k values used for the simulation of the exchange reaction of trimethyl orthoformate with 2-chloroethanol**.**

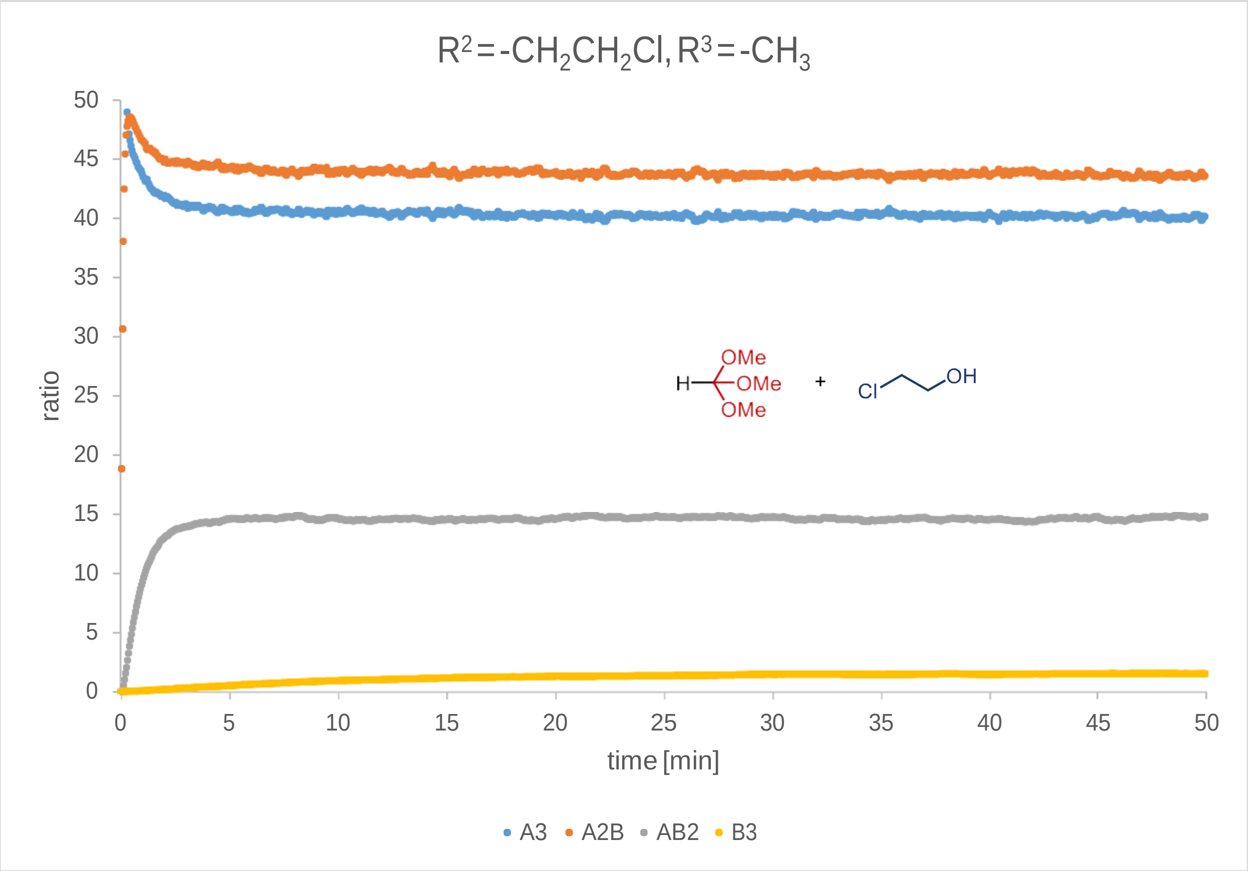

Graph S25: Simulated time-dependent product distribution plot of the exchange reaction of trimethyl orthoformate with 2-chloroethanol**.**

Conclusion: To further support our hypothesis that the observed time-dependent product distributions can be explained by differences in the stability of oxonium intermediates and corresponding differences in reaction rates, basic kinetic simulations were performed. All estimated k values and associated simulations **match the experimental profiles very well on a qualitative level** (Graph S14 and S20, Graph S17 and S21, Graph S19 and S22).

Hydrolysis rates

Reaction scheme:

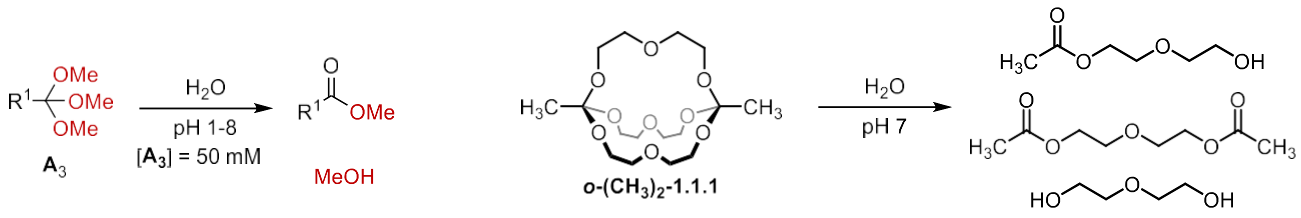

The orthoester hydrolysis reactions were carried out according to general procedure C.

Summary of results:

R^1		pH 8	pH 7	pH 6	pH 5	pH 4	pH 3	pH 1
$-CH_3$	$t_{1/2}$ [min]	60	10	2	<1			
	k_{obs} [s^{-1}]	1.8×10^{-4}	1.3×10^{-3}	6.1×10^{-3}				
-H	$t_{1/2}$ [min]	>1000	510	70	20	7	<1	
	k_{obs} [s^{-1}]		2.5×10^{-5}	1.6×10^{-4}	8.8×10^{-4}	2.0×10^{-3}		
$-CH_2Cl$	$t_{1/2}$ [min]		>1000	660	120	30	<1	
	k_{obs} [s^{-1}]			1.7×10^{-5}	8.6×10^{-5}	2.9×10^{-4}		
-triazolium[a]	$t_{1/2}$ [min]							>1000
	k_{obs} [s^{-1}]							1.6×10^{-6}
$-CCl_3$	$t_{1/2}$ [min]							>1000[b]
***o*-(CH$_3$)$_2$-1.1.1**	$t_{1/2}$ [min]		30					
	k_{obs} [s^{-1}]		3.5×10^{-4}					

Table S10: Hydrolysis rates of orthoesters. $t_{1/2}$: Half-life of starting material, defined as point when 50% of starting material were consumed. [a] 1-benzyl-3-methyl-4-(trimethoxymethyl)-1*H*-1,2,3-triazol-3-ium. [b] 400 µL DMSO added to increase solubility of starting material.

R^1	pH	$t_{1/2}$ [min]	avg. $t_{1/2}$ [min]	u [min]	$U_{95\%}$ [min]
-H	6	70			
-H	6	71	70	0.7	±3
-H	6	68			

Table S11: Representative calculation of confidence interval for triplicate orthoester hydrolysis reaction of trimethyl orthoformate. u: standard uncertainty = $s/\sqrt{\Box}$; s: standard deviation; n: number of measurements; U: 95% confidence interval = $t_{(0.05,2)} \times u$; $t_{(\alpha,\, n-1)}$: student-t distribution at a probability α.[8]

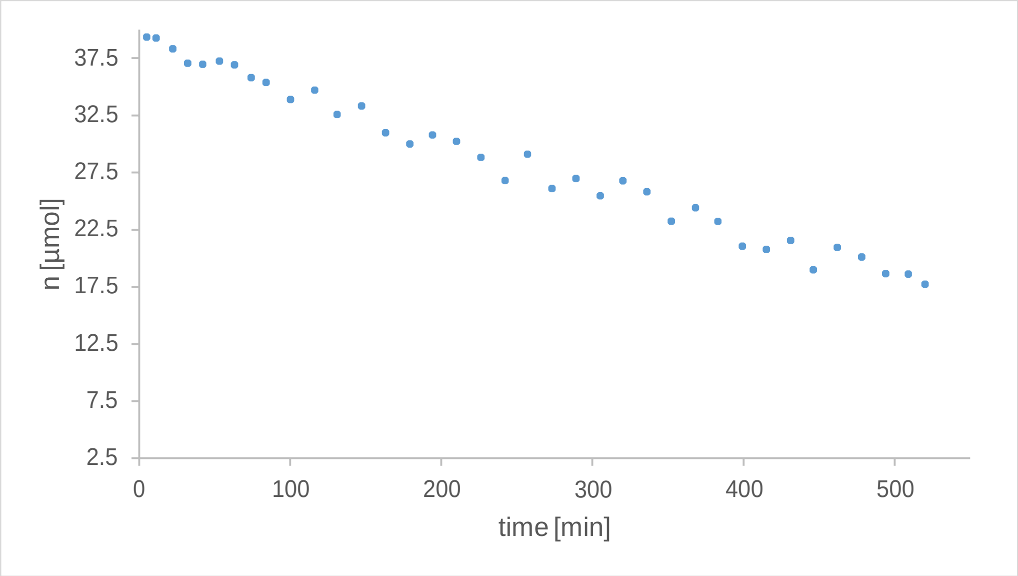

Graph S26: Representative plot of orthoester hydrolysis reaction (R[1] = -H, pH 7). Amount of starting material over time.

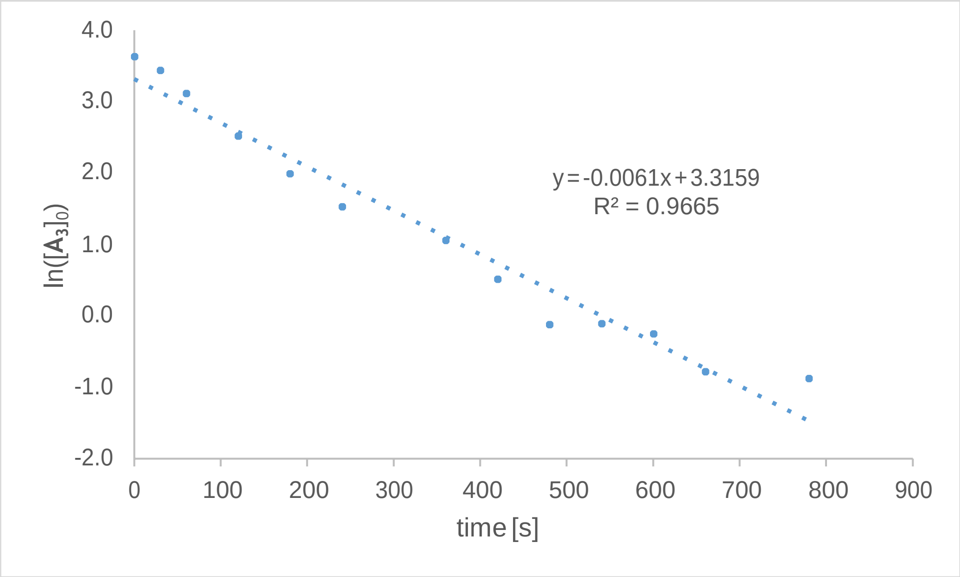

Graph S27: Plot of ln([**A**$_3$]$_0$) versus time for R[1] = -CH_3 at pH 6. Blue dots: Experimental points. Blue line: Linear fit.

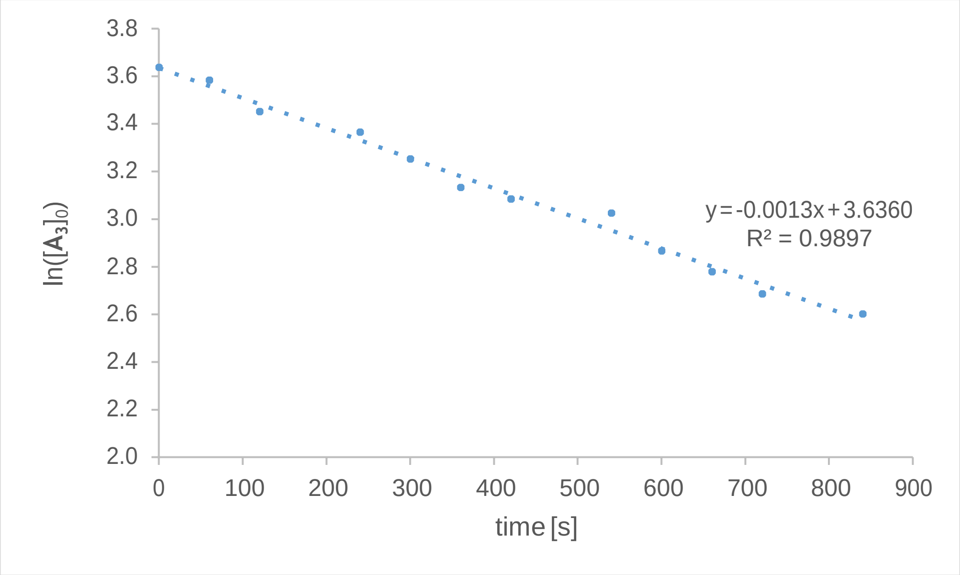

Graph S28: Plot of ln([**A3**]$_0$) versus time for R^1 = -CH_3 at pH 7. Blue dots: Experimental points. Blue line: Linear fit.

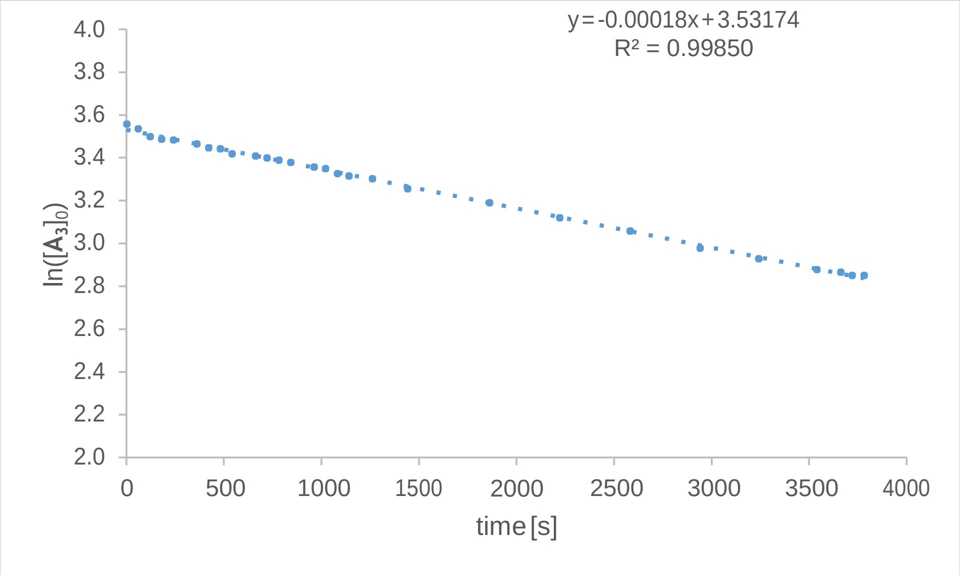

Graph S29: Plot of ln([**A3**]$_0$) versus time for R^1 = -CH_3 at pH 8. Blue dots: Experimental points. Blue line: Linear fit.

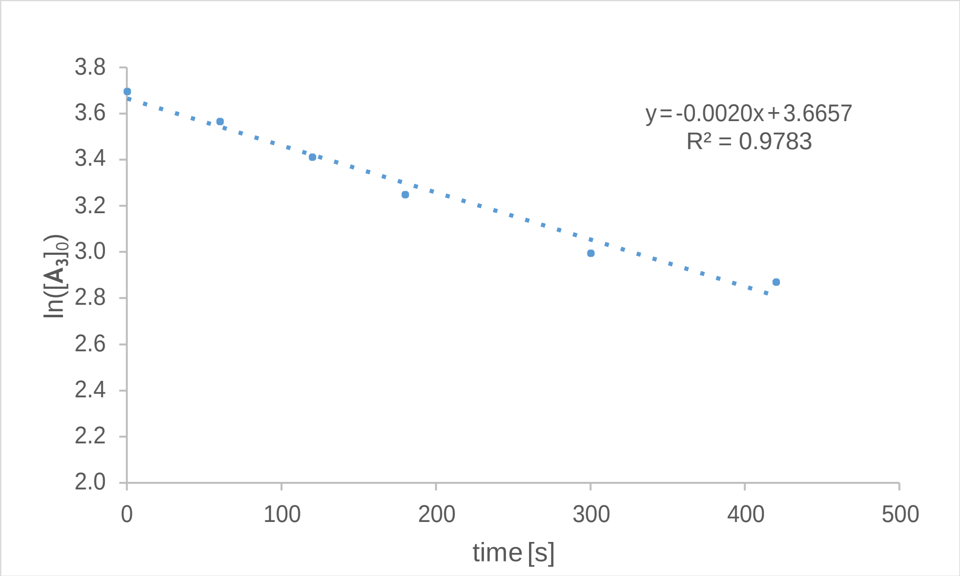

Graph S30: Plot of ln([**A3**]$_0$) versus time for R[1] = -H at pH 4. Blue dots: Experimental points. Blue line: Linear fit.

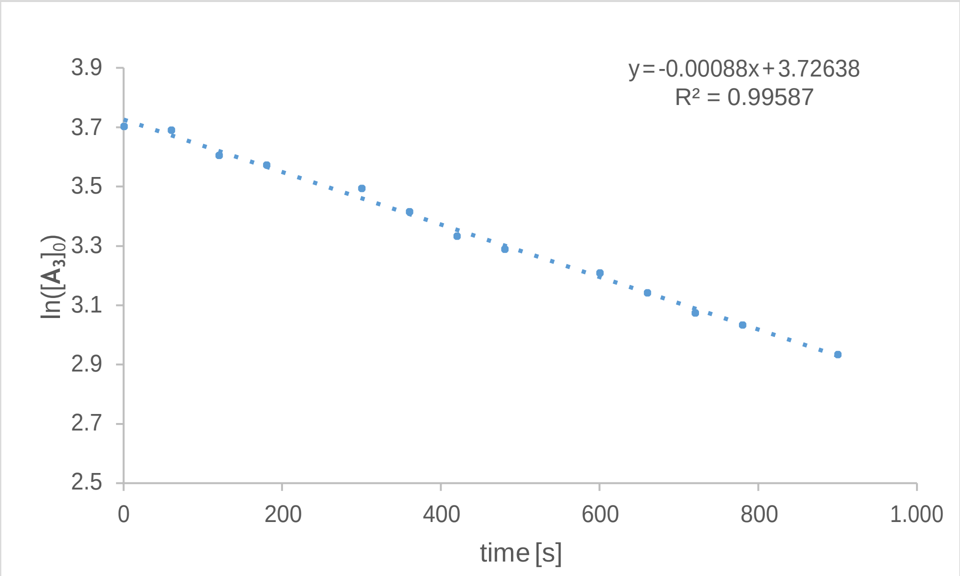

Graph S31: Plot of ln([**A3**]$_0$) versus time for R[1] = -H at pH 5. Blue dots: Experimental points. Blue line: Linear fit.

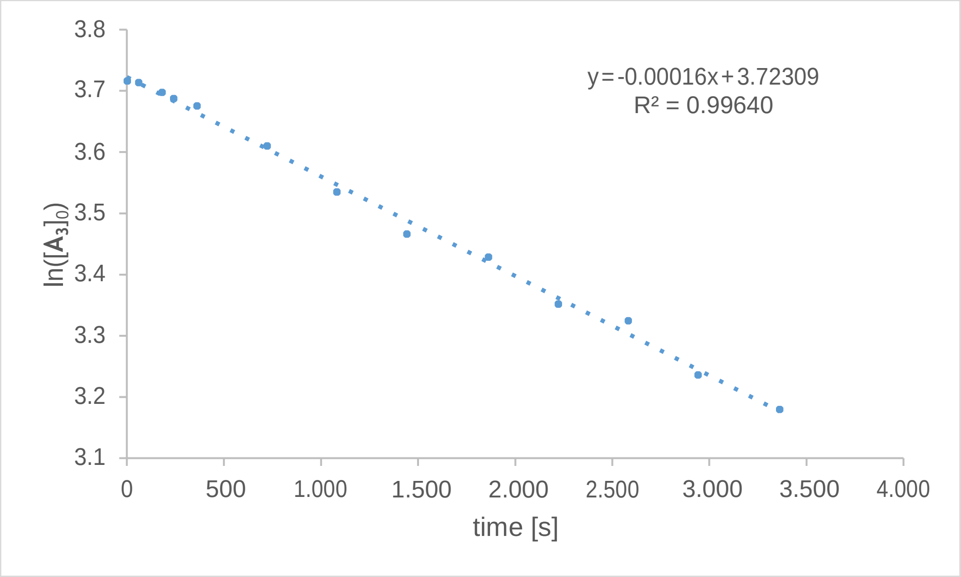

Graph S32: Plot of ln([**A3**]$_0$) versus time for R[1] = -H at pH 6. Blue dots: Experimental points. Blue line: Linear fit.

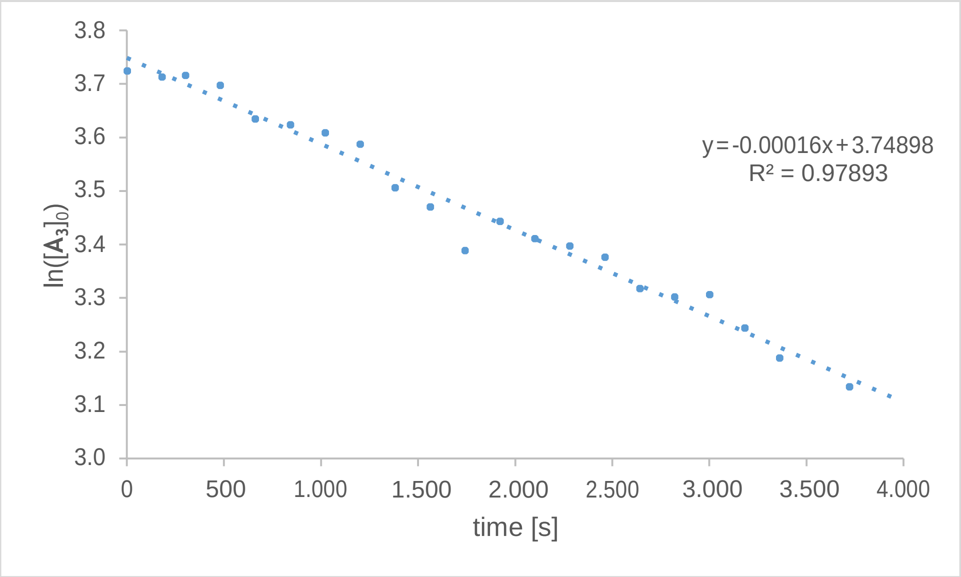

Graph S33: Reproduced plot of ln([**A3**]$_0$) versus time for R[1] = -H at pH 6. Blue dots: Experimental points. Blue line: Linear fit.

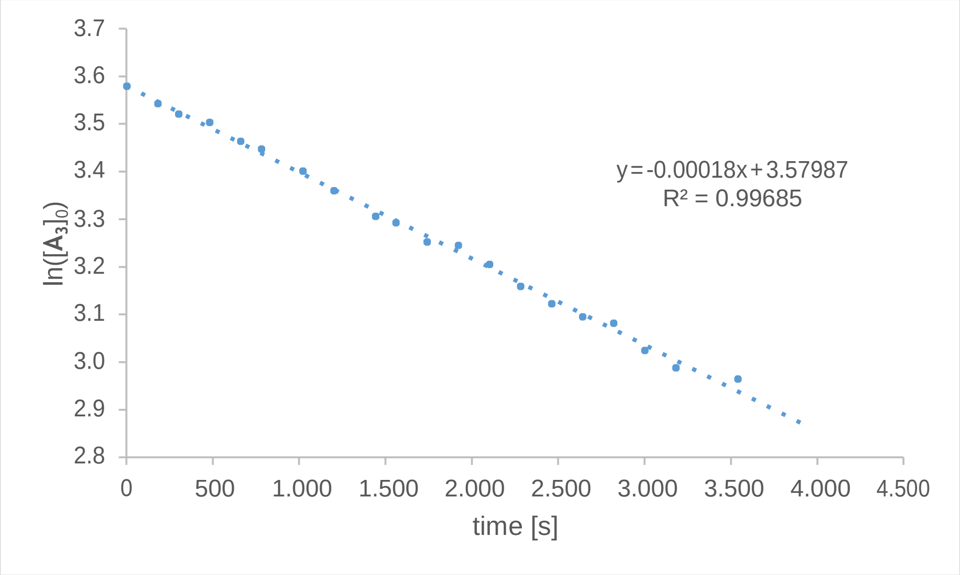

Graph S34: Reproduced plot of ln([**A**$_3$]$_0$) versus time for R[1] = -H at pH 6. Blue dots: Experimental points. Blue line: Linear fit.

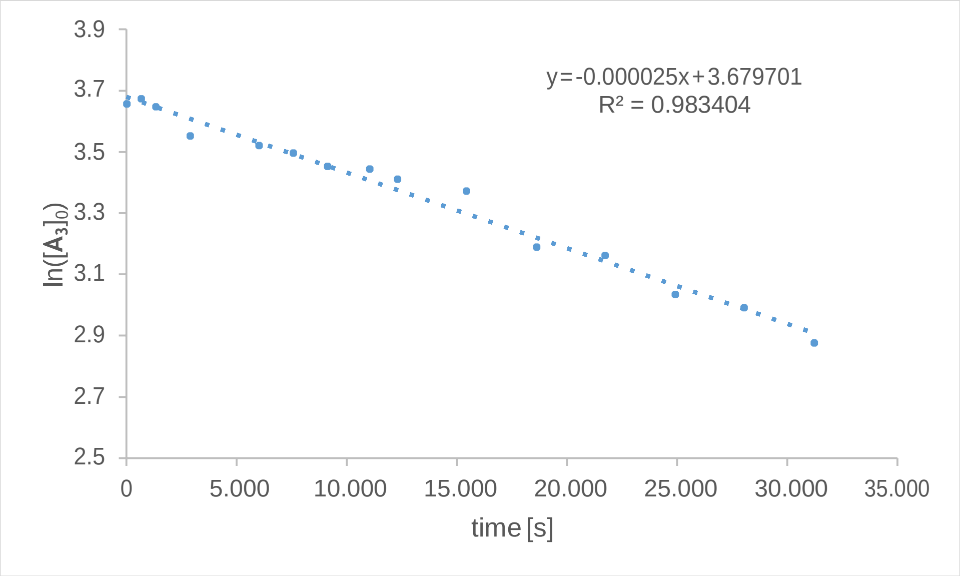

Graph S35: Plot of ln([**A**$_3$]$_0$) versus time for R[1] = -H at pH 7. Blue dots: Experimental points. Blue line: Linear fit.

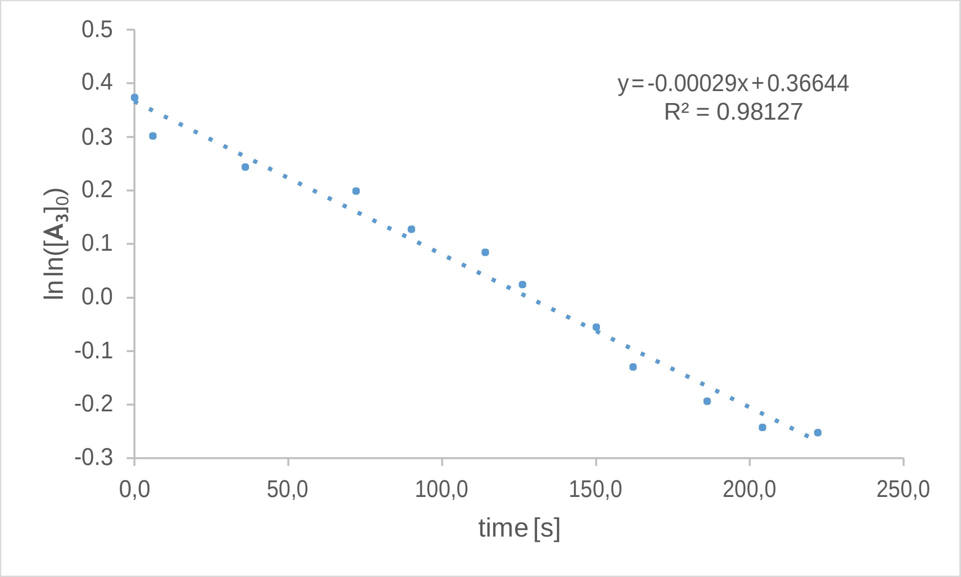

Graph S36: Plot of ln([**A**$_3$]$_0$) versus time for R[1] = -CH_2Cl at pH 4. Blue dots: Experimental points. Blue line: Linear fit.

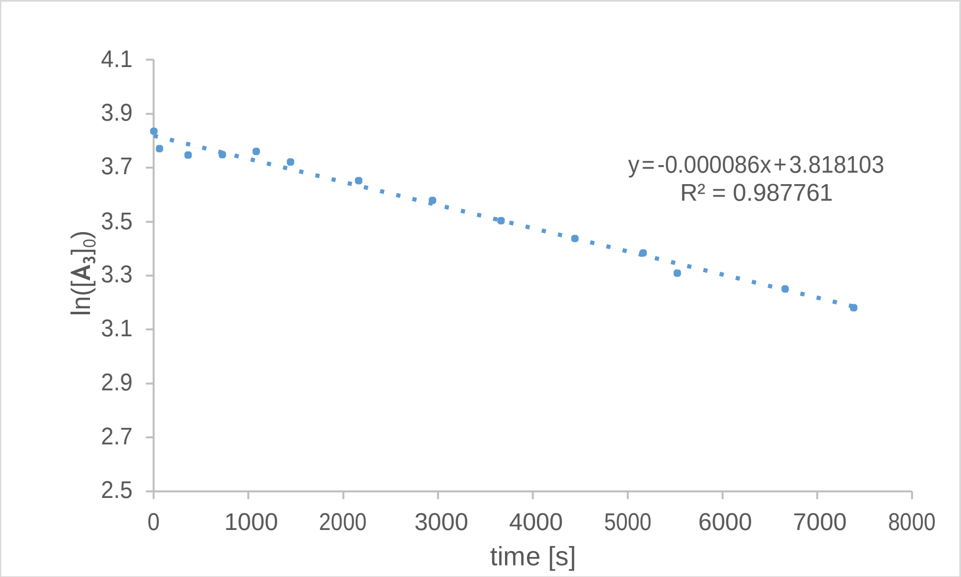

Graph S37: Plot of ln([**A**$_3$]$_0$) versus time for R[1] = -CH_2Cl at pH 5. Blue dots: Experimental points. Blue line: Linear fit.

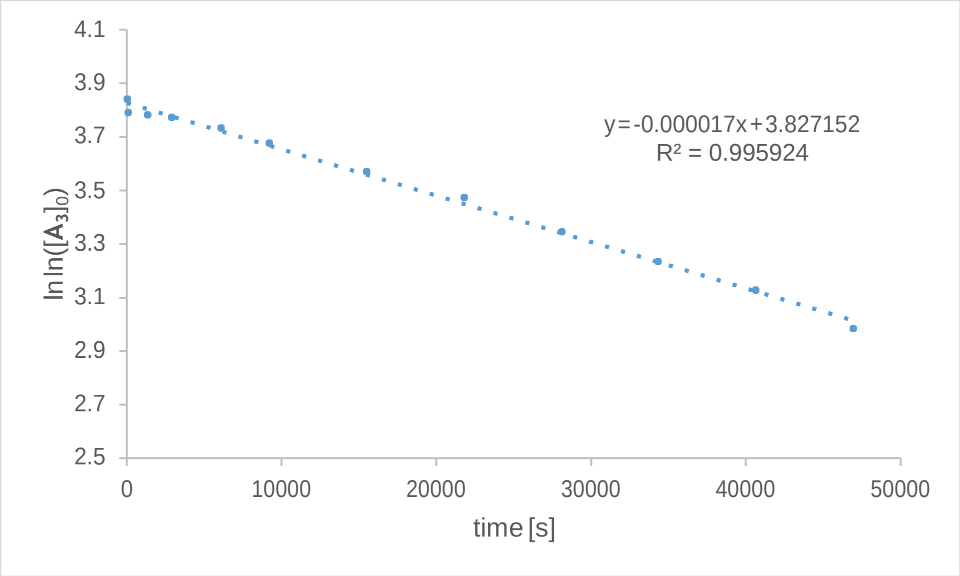

Graph S38: Plot of ln([**A**$_3$]$_0$) versus time for R^1 = -CH_2Cl at pH 6. Blue dots: Experimental points. Blue line: Linear fit.

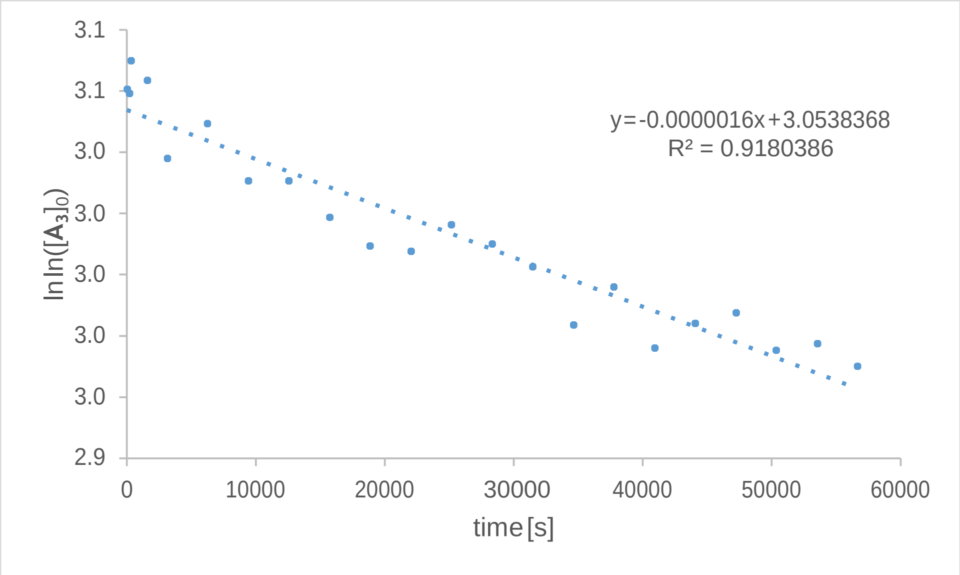

Graph S39: Plot of ln([**A**$_3$]$_0$) versus time for R^1 = -triazolium at pH 1. Blue dots: Experimental points. Blue line: Linear fit.

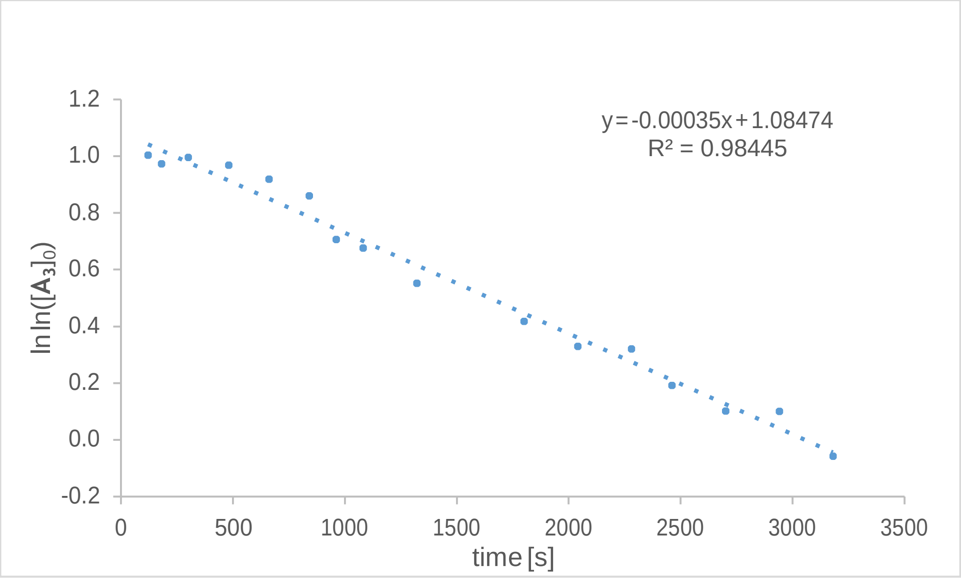

Graph S40: Plot of ln([***o*-(CH$_3$)$_2$-1.1.1**]$_0$) versus time at pH 7. Blue dots: Experimental points. Blue line: Linear fit.

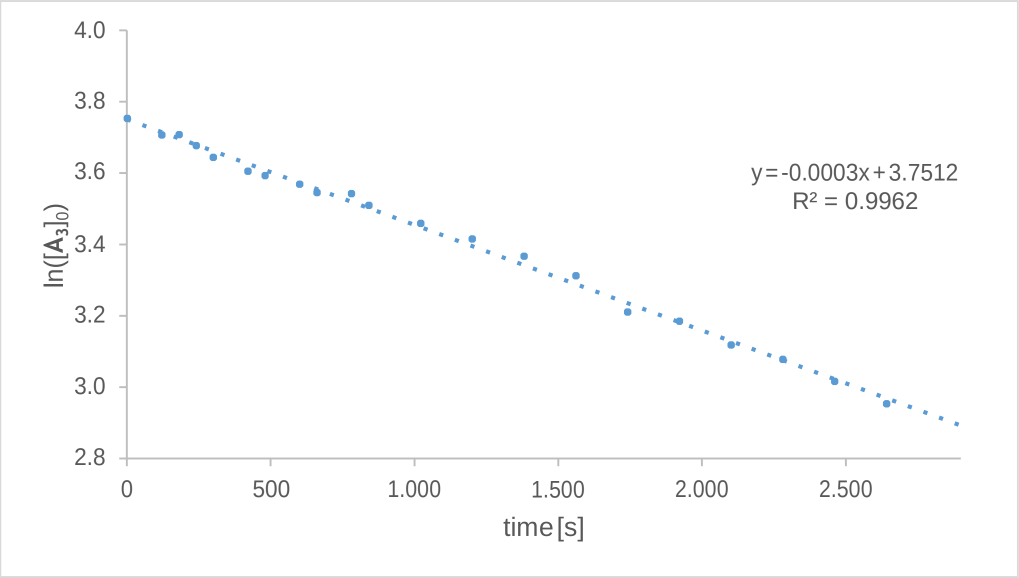

Graph S41: Plot of ln([**A$_3$**]$_0$) versus time for R^1 = -H at pH 4 with addition of 400 µL DMSO. Blue dots: Experimental points. Blue line: Linear fit.

R^1	pH	Solvent	$t_{1/2}$ [min]	k$_{obs}$ [s^{-1}]
-H	4	550 µL buffer solution	7	2.0×10^{-3}
-H	4	250 µL buffer solution + 400 µL DMSO	40	3.0×10^{-4}

Table S12: Influence of DMSO addition on $t_{1/2}$. k$_{obs}$ is decreased by ca. one order of magnitude.

5. Crystallographic Data

Compound [Na^+⊂*o*-(*n*C_4H_9)$_2$-1.1.1]$BArF^-$

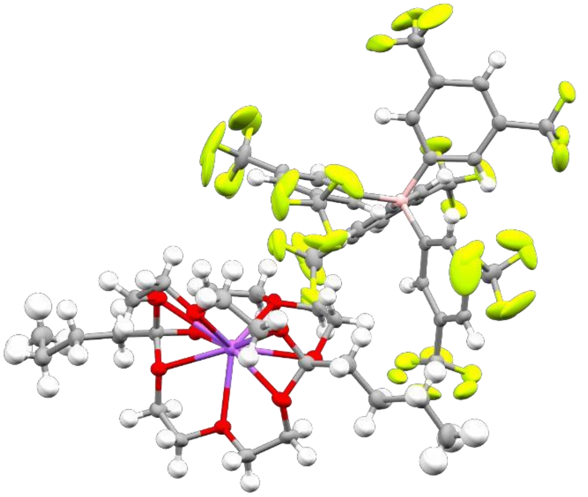

Empirical formula	$C_{54}H_{54}F_{24}NaO_9$
Formula weight	1336.77
Temperature/K	293.15
Crystal system	triclinic
Space group	P-1
a/Å	12.434(4)
b/Å	12.984(5)
c/Å	20.025(7)
α/°	93.798(13)
β/°	107.540(12)
γ/°	104.457(8)
Volume/Å^3	2949.7(18)
Z	2
ρ_{calc}g/cm^3	1.505
μ/mm^{-1}	0.156
F(000)	1364.0
Crystal size/mm^3	0.191 × 0.171 × 0.052
Radiation	MoKα (λ = 0.71073)
2Θ range for data collection/°	4.192 to 50.092
Index ranges	$-14 \leq h \leq 14, -15 \leq k \leq 15, -23 \leq l \leq 23$
Reflections collected	119640
Independent reflections	10400 [R_{int} = 0.1265, R_{sigma} = 0.0411]
Data/restraints/parameters	10400/512/973
Goodness-of-fit on F^2	1.045
Final R indexes [I>=2σ (I)]	R_1 = 0.0492, wR_2 = 0.1186
Final R indexes [all data]	R_1 = 0.0610, wR_2 = 0.1261
Largest diff. peak/hole / e Å^{-3}	0.41/-0.34

[Na^+⊂*o*-(*n*C_4H_9)$_2$-1.1.1]$BArF^-$ was crystallized by solvent layering of hexane over solution of **[Na^+⊂*o*-(*n*C_4H_9)$_2$-1.1.1]$BArF^-$** in chloroform. The cif-file was deposited in the Cambridge structural database under identifier CCDC 1823728.

Compound [Na^+⊂*o*-($CH_2C_6H_5$)$_2$-1.1.1]$BArF^-$

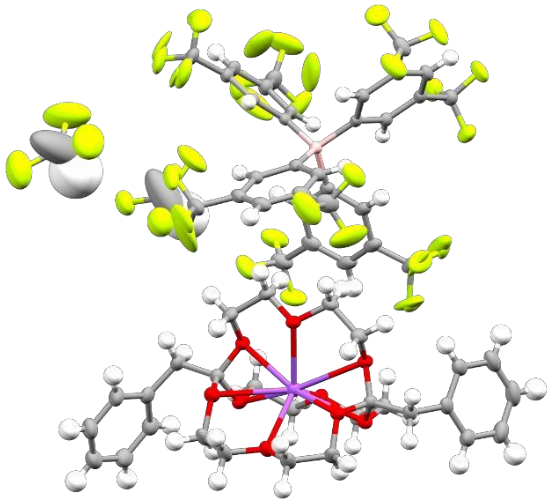

Empirical formula	$C_{62}H_{52}BF_{30}NaO_9$
Formula weight	1544.83
Temperature/K	150.01
Crystal system	monoclinic
Space group	$P2_1/n$
a/Å	20.158(5)
b/Å	17.085(5)
c/Å	20.698(6)
α/°	90
β/°	101.158(14)
γ/°	90
Volume/$Å^3$	6994(3)
Z	4
$\rho_{calc}g/cm^3$	1.467
μ/mm^{-1}	0.155
F(000)	3128.0
Crystal size/mm^3	0.327 × 0.239 × 0.146
Radiation	MoKα (λ = 0.71073)
2Θ range for data collection/°	4.666 to 55.702
Index ranges	$-25 \leq h \leq 26, -22 \leq k \leq 22, -27 \leq l \leq 27$
Reflections collected	122973
Independent reflections	16615 [R_{int} = 0.0474, R_{sigma} = 0.0304]
Data/restraints/parameters	16615/346/1040
Goodness-of-fit on F^2	1.036
Final R indexes [I>=2σ (I)]	R_1 = 0.0752, wR_2 = 0.2112
Final R indexes [all data]	R_1 = 0.0998, wR_2 = 0.2342
Largest diff. peak/hole / e $Å^{-3}$	0.99/-0.86

[Na^+⊂*o*-($CH_2C_6H_5$)$_2$-1.1.1]$BArF^-$ was crystallized by solvent layering of hexane over solution of **[Na^+⊂*o*-($CH_2C_6H_5$)$_2$-1.1.1]$BArF^-$** in chloroform. The cif-file was deposited in the Cambridge structural database under identifier CCDC 1823774.

Compound [Na^+⊂*o*-$(C_6H_5)_2$-1.1.1]$BArF^-$

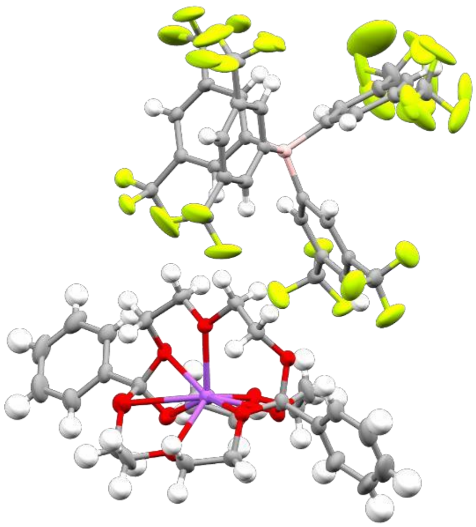

Empirical formula	$C_{58}H_{46}BF_{24}NaO_9$
Formula weight	1356.75
Temperature/K	149.96
Crystal system	orthorhombic
Space group	Pbca
a/Å	19.142(4)
b/Å	24.665(5)
c/Å	26.029(6)
α/°	90
β/°	90
γ/°	90
Volume/Å^3	12289(5)
Z	8
ρ_{calc}g/cm^3	1.488
μ/mm^{-1}	0.153
F(000)	5584.0
Crystal size/mm^3	0.306 × 0.193 × 0.124
Radiation	MoKα (λ = 0.71073)
2Θ range for data collection/°	4.13 to 54.038
Index ranges	$-24 \leq h \leq 21$, $-23 \leq k \leq 31$, $-33 \leq l \leq 26$
Reflections collected	80330
Independent reflections	13422 [R_{int} = 0.0453, R_{sigma} = 0.0350]
Data/restraints/parameters	13422/70/920
Goodness-of-fit on F^2	1.017
Final R indexes [I>=2σ (I)]	R_1 = 0.0451, wR_2 = 0.0786
Final R indexes [all data]	R_1 = 0.0734, wR_2 = 0.1113
Largest diff. peak/hole / e Å^{-3}	0.51/-0.39

[Na^+⊂*o*-$(C_6H_5)_2$-1.1.1]$BArF^-$ was crystallized by solvent layering of hexane over solution of **[Na^+⊂*o*-$(C_6H_5)_2$-1.1.1]$BArF^-$** in chloroform. The cif-file was deposited in the Cambridge structural database under identifier CCDC 1823730.

Compound [Na$^+$⊂*o*-(C≡C-Si(CH$_3$)$_3$)$_2$-1.1.1]BArF$^-$

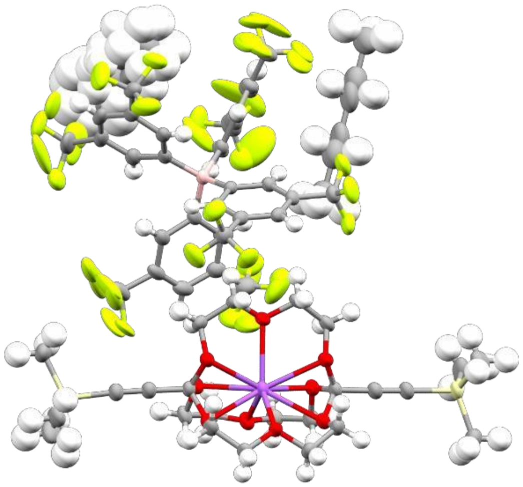

Empirical formula	$C_{65}H_{75}BF_{24}NaO_9Si_2 \cdot C_9H_{21}$ (1.5 x hexane)
Formula weight	1546.23
Temperature/K	150.00(14)
Crystal system	triclinic
Space group	P-1
a/Å	13.9002(3)
b/Å	14.0497(3)
c/Å	21.1609(5)
α/°	101.4174(19)
β/°	103.600(2)
γ/°	101.486(16)
Volume/Å^3	3804.08(16)
Z	2
ρ_{calc}g/cm^3	1.350
μ/mm^{-1}	0.161
F(000)	1594.0
Crystal size/mm^3	0.235 × 0.157 × 0.122
Radiation	MoKα (λ = 0.71073)
2Θ range for data collection/°	5.644 to 58.92
Index ranges	$-19 \le h \le 19$, $-19 \le k \le 19$, $-28 \le l \le 28$
Reflections collected	58033
Independent reflections	18507 [R_{int} = 0.0434, R_{sigma} = 0.0526]
Data/restraints/parameters	18507/872/1095
Goodness-of-fit on F^2	1.043
Final R indexes [I>=2σ (I)]	R_1 = 0.0704, wR_2 = 0.1618
Final R indexes [all data]	R_1 = 0.1137, wR_2 = 0.1836
Largest diff. peak/hole / e Å^{-3}	0.37/-0.36

[Na$^+$⊂*o*-(C≡C-Si(CH$_3$)$_3$)$_2$-1.1.1]BArF$^-$ was crystallized by solvent layering of hexane over solution of **[Na$^+$⊂*o*-(C≡C-Si(CH$_3$)$_3$)$_2$-1.1.1]BArF$^-$** in chloroform. The cif-file was deposited in the Cambridge structural database under identifier CCDC 1832928.

Compound [Na^+⊂*o*-(CH_2Cl)$_2$-1.1.1]BArF$^-$

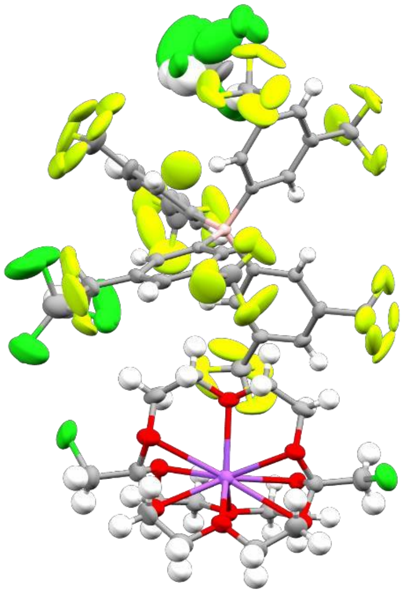

Empirical formula	$C_{48}H_{40}BCl_2F_{24}NaO_9 \cdot C_2H_{1.87}Cl_6$ (2 x $CHCl_3$)
Formula weight	1560.19
Temperature/K	150.00(14)
Crystal system	monoclinic
Space group	$P2_1/c$
a/Å	13.0127(4)
b/Å	19.6275(7)
c/Å	25.4722(8)
α/°	90
β/°	96.246(3)
γ/°	90
Volume/Å^3	6467.1(4)
Z	4
ρ_{calc}g/cm^3	1.602
μ/mm^{-1}	0.475
F(000)	3128.0
Crystal size/mm^3	0.2195 × 0.1546 × 0.1189
Radiation	MoKα (λ = 0.71073)
2Θ range for data collection/°	5.85 to 59.054
Index ranges	$-17 \le h \le 17$, $-23 \le k \le 26$, $-34 \le l \le 32$
Reflections collected	44739
Independent reflections	15699 [R_{int} = 0.0343, R_{sigma} = 0.0417]
Data/restraints/parameters	15699/13107/1290
Goodness-of-fit on F^2	1.015
Final R indexes [I>=2σ (I)]	R_1 = 0.0629, wR_2 = 0.1451
Final R indexes [all data]	R_1 = 0.0975, wR_2 = 0.1671
Largest diff. peak/hole / e Å^{-3}	0.68/-0.67

[Na^+⊂*o*-(CH_2Cl)$_2$-1.1.1]BArF$^-$ was crystallized by solvent layering of hexane over solution of **[Na^+⊂*o*-(CH_2Cl)$_2$-1.1.1]BArF$^-$** in chloroform. The cif-file was deposited in the Cambridge structural database under identifier CCDC 1823819.

Compound [Na^+⊂*o*-(C≡CH)$_2$-1.1.1]BArF$^-$

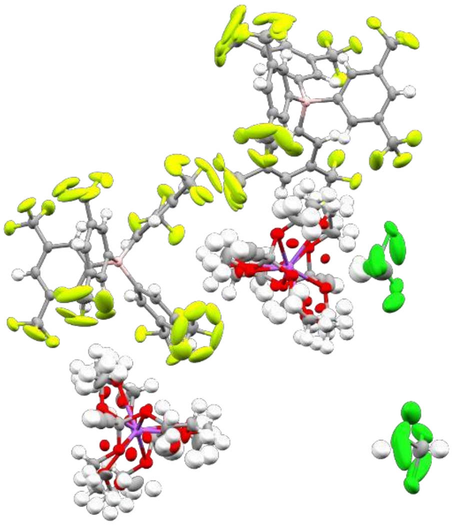

Empirical formula	$C_{100}H_{76}B_2F_{48}Na_2O_{18} \cdot C_2H_2Cl_6$ (2 x $CHCl_3$)
Formula weight	2783.94
Temperature/K	293.15
Crystal system	triclinic
Space group	P-1
a/Å	16.654(8)
b/Å	18.497(8)
c/Å	20.536(9)
α/°	70.108(16)
β/°	83.341(17)
γ/°	89.951(13)
Volume/Å^3	5903(5)
Z	2
ρ_{calc}g/cm^3	1.566
μ/mm^{-1}	0.291
F(000)	2800.0
Crystal size/mm^3	0.289 × 0.13 × 0.111
Radiation	MoKα (λ = 0.71073)
2Θ range for data collection/°	4.25 to 46.518
Index ranges	$-18 \le h \le 18$, $-20 \le k \le 20$, $-22 \le l \le 22$
Reflections collected	122948
Independent reflections	16871 [R_{int} = 0.0556, R_{sigma} = 0.0391]
Data/restraints/parameters	16871/1118/2075
Goodness-of-fit on F^2	1.053
Final R indexes [I>=2σ (I)]	R_1 = 0.0632, wR_2 = 0.1442
Final R indexes [all data]	R_1 = 0.0783, wR_2 = 0.1547
Largest diff. peak/hole / e Å^{-3}	0.67/-0.58

[Na^+⊂*o*-(C≡CH)$_2$-1.1.1]BArF$^-$ was crystallized by solvent layering of hexane over solution of **[Na^+⊂*o*-(C≡CH)$_2$-1.1.1]BArF$^-$** in chloroform. The cif-file was deposited in the Cambridge structural database under identifier CCDC 1823845.

Compound [Na$^+$⊂1]BArF$^-$

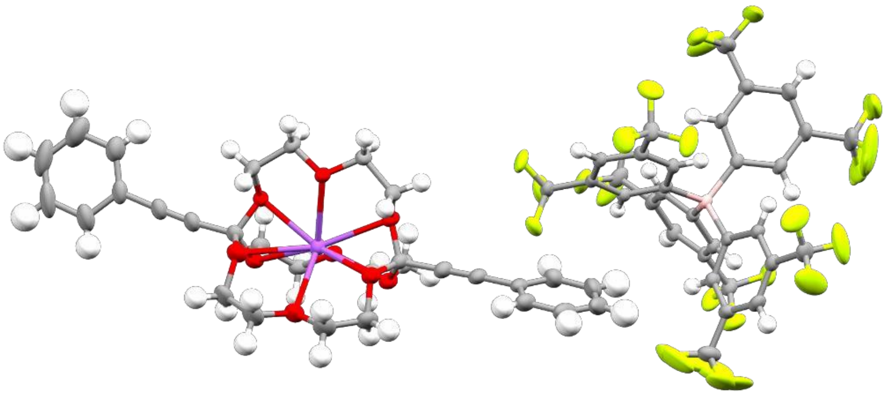

Empirical formula	$C_{62}H_{46}BF_{24}NaO_9$
Formula weight	1424.79
Temperature/K	293.15
Crystal system	triclinic
Space group	P-1
a/Å	14.295(8)
b/Å	14.544(10)
c/Å	17.347(11)
α/°	100.67(3)
β/°	107.46(2)
γ/°	105.51(3)
Volume/Å^3	3174(4)
Z	2
ρ_{calc}g/cm^3	1.491
μ/mm^{-1}	0.151
F(000)	1444.0
Crystal size/mm^3	0.177 × 0.169 × 0.118
Radiation	MoKα (λ = 0.71073)
2Θ range for data collection/°	4.214 to 55.822
Index ranges	$-18 \le h \le 18$, $-19 \le k \le 19$, $-22 \le l \le 22$
Reflections collected	85895
Independent reflections	15097 [R_{int} = 0.0405, R_{sigma} = 0.0310]
Data/restraints/parameters	15097/159/930
Goodness-of-fit on F^2	1.029
Final R indexes [I>=2σ (I)]	R_1 = 0.0524, wR_2 = 0. 1185
Final R indexes [all data]	R_1 = 0. 0734, wR_2 = 0.1311
Largest diff. peak/hole / e Å^{-3}	0.81/-0.61

[Na$^+$⊂1]BArF$^-$ was crystallized by solvent layering of hexane over solution of **[Na$^+$⊂1]BArF$^-$** in chloroform. The cif-file was deposited in the Cambridge structural database under identifier CCDC 1823126.

Compound [Li$^+$⊂*o*-(H)$_2$-1.1.1]BArF$^-$

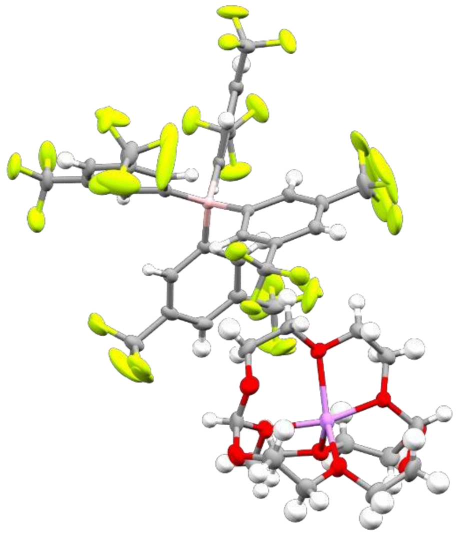

Empirical formula	$C_{46}H_{38}BF_{24}LiO_9$
Formula weight	1208.51
Temperature/K	149.95(20)
Crystal system	orthorhombic
Space group	$P2_12_12_1$
a/Å	12.8768(3)
b/Å	17.6754(4)
c/Å	22.1097(5)
α/°	90
β/°	90
γ/°	90
Volume/Å^3	5032.20(2)
Z	4
ρ_{calc}g/cm^3	1.595
μ/mm^{-1}	0.166
F(000)	2444.0
Crystal size/mm^3	0.268 × 0.142 × 0.055
Radiation	MoKα (λ = 0.71073)
2Θ range for data collection/°	5.59 to 59.1
Index ranges	$-17 \leq h \leq 17$, $-24 \leq k \leq 24$, $-25 \leq l \leq 29$
Reflections collected	74805
Independent reflections	12892 [R_{int} = 0.0393, R_{sigma} = 0.0316]
Data/restraints/parameters	12892/1200/979
Goodness-of-fit on F^2	1.051
Final R indexes [I>=2σ (I)]	R_1 = 0.0399, wR_2 = 0. 0798
Final R indexes [all data]	R_1 = 0. 0577, wR_2 = 0.00872
Largest diff. peak/hole / e Å^{-3}	0.48/-0.36
Flack parameter	-0.23(13)

[Li$^+$⊂*o*-(H)$_2$-1.1.1]BArF$^-$ was crystallized by solvent layering of hexane over solution of **[Li$^+$⊂*o*-(H)$_2$-1.1.1]BArF$^-$** in chloroform. The cif-file was deposited in the Cambridge structural database under identifier CCDC 1824092.

Comparison of M^+-O distances and O-C-O angles

entry	Compound	M^+-O distance [Å] (orthoester oxygens)	M^+-O distance [Å] (chain oxygens)	O-C-O angle [°]
1	**[Na^+⊂*o*-(*n*C_4H_9)$_2$-1.1.1]BArF$^-$**	2.445(2), 2.494(2), 2.532(2), 2.620(2), 2.799(2), 2.918(2)	2.484(2), 2.525(2), 2.533(2)	103.9(2), 104.7(2), 105.3(2), 105.5(2), 106.0(2), 106.7(2)
2	**[Na^+⊂*o*-($CH_2C_6H_5$)$_2$-1.1.1]BArF$^-$**	2.472(2), 2.504(2), 2.610(2), 2.640(2), 2.706(2), 2.756(2)	2.514(2), 2.538(2), 2.567(2)	104.7(2), 104.9(2), 105.1(2), 105.5(2), 106.3(2), 106.5(2)
3	**[Na^+⊂*o*-(C_6H_5)$_2$-1.1.1]BArF$^-$**	2.320(2), 2.399(2), 2.709(2), 2.771(2), 2.782(2), 3.530(2)	2.368(2), 2.419(2), 2.456(2)	103.6(1), 104.6(1), 105.1(2), 106.8(2), 109.0(2), 109.7(2)
4	**[Na^+⊂*o*-(C≡C-Si(CH_3)$_3$)$_2$-1.1.1]BArF$^-$**	2.479(3), 2.501(2), 2.513(2), 2.517(3), 2.538(2), 2.591(2)	2.542(2), 2.591(2), 2.674(3)	105.3(2), 105.4(2), 105.6(2), 105.6(2), 105.8(2), 106.1(2)
5	**[Na^+⊂*o*-(CH_2Cl)$_2$-1.1.1]BArF$^-$**	2.467(2), 2.475(2), 2.552(2), 2.584(2), 2.804(2), 2.889(2)	2.483(2), 2.501(2), 2.572(2)	105.3(2), 105.6(2), 106.6(2), 106.7(2), 106.7(2), 107.1(2)
6	**[Na^+⊂*o*-(C≡CH)$_2$-1.1.1]BArF$^-$**	2.464(4), 2.518(4), 2.541(4), 2.546(4), 2.547(4), 2.663(4)	2.526(4), 2.663(4), 2.671(5)	104.3(3), 104.6(3), 105.3(3), 105.3(3), 106.5(3), 106.9(3)
7	**[Na^+⊂1]BArF$^-$**	2.428(2), 2.456(2), 2.508(2), 2.549(2), 2.601(2), 2.694(2)	2.563(2), 2.610(3), 2.614(2)	105.5(2), 105.5(2), 105.5(2), 105.6(2), 106.1(2), 106.2(2)
8	**[Li^+⊂*o*-(H)$_2$-1.1.1]BArF$^-$**	1.957(5), 1,963(5), 2.940(5), 3.157(5), 3.620(5), 3.672(5)	2.163(5), 2.176(6), 2.232(5)	105.9(2), 106.4(2), 110.4(2), 110.9(3), 112.1(3), 112.2(2)

Table S13: M^+-O distances and O-C-O angles derived from solid state structures of orthoester cryptands.

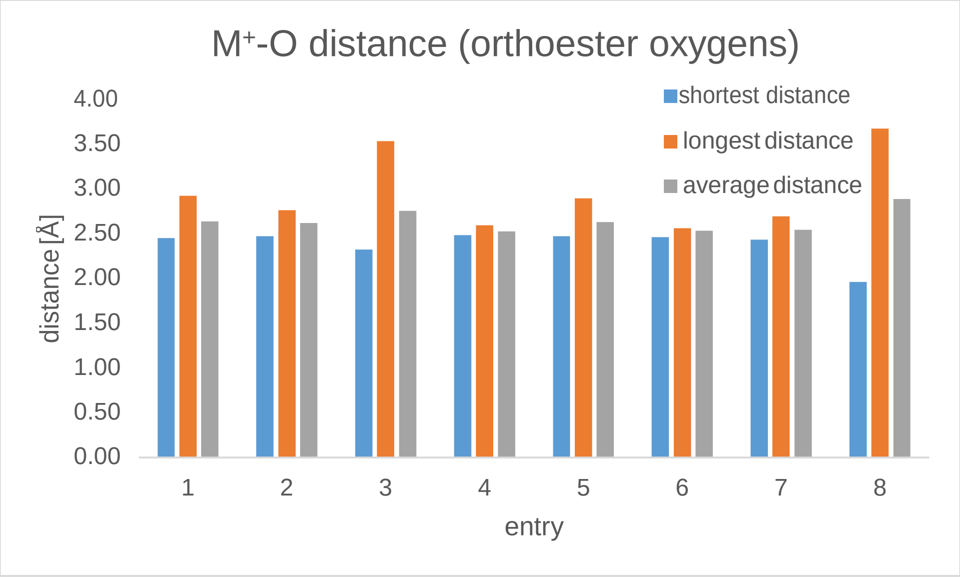

Graph S42: Comparison of M^+-O distances (orthoester oxygens).

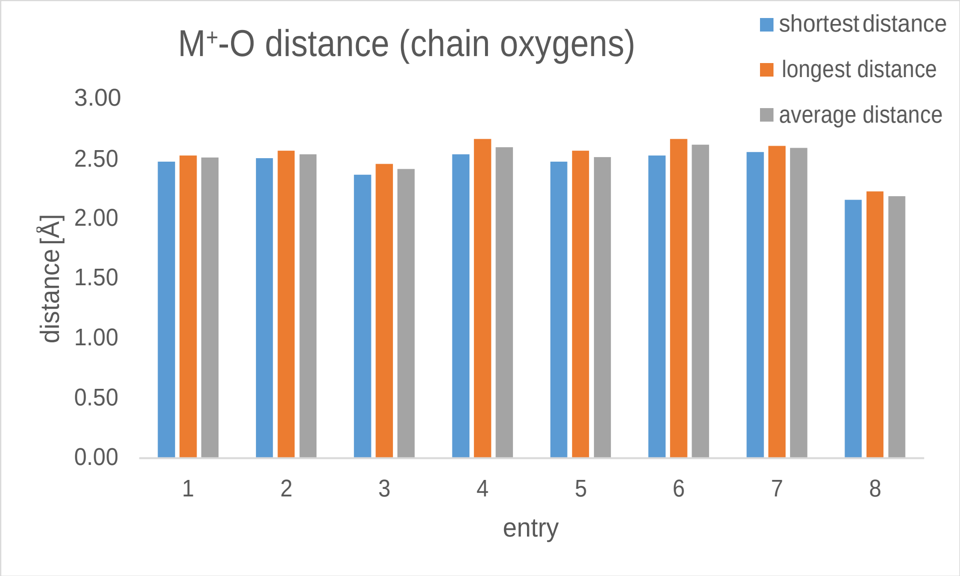

Graph S43: Comparison of M^+-O distances (chain oxygens).

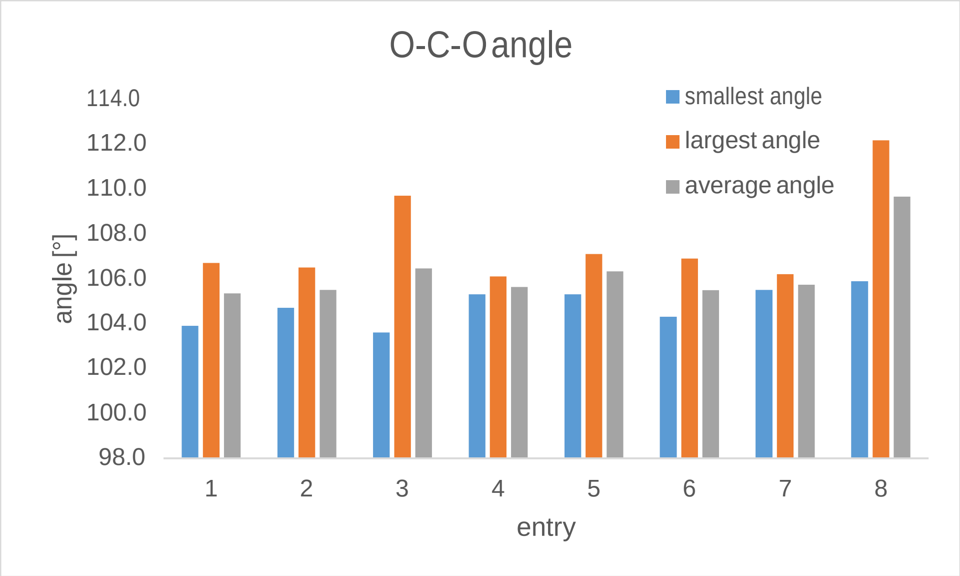

Graph S44: Comparison of O-C-O angles.

6. References

(1) DeWolfe, R. H. *Synthesis* **1974**, *3*, 152–172.

(2) Talbott, R. L. *Fluorocarbon Orthoesters*. US3415847A, 1968.

(3) Kirchmeyer, S.; Mertens, A.; Arvanaghi, M.; Olah, G. A. *Synthesis* **1983**, *6*, 498–500.

(4) Yamamoto, K.; Abrecht, S.; Scheffold, R. *Chimia* **1991**, *45*, 86–88.

(5) Shyshov, O.; Brachvogel, R. C.; Bachmann, T.; Srikantharajah, R.; Segets, D.; Hampel, F.; Puchta, R.; von Delius, M. *Angew. Chem., Int. Ed.* **2017**, *56* (3), 776–781.

(6) Tanaka, R.; Rodgers, M.; Simonaitx, R.; Millers, S. I.; Cor, H. J. *Tetrahedron* **1971**, *27*, 2651–2669.

(7) Brachvogel, R.-C.; Hampel, F.; von Delius, M. *Nat. Commun.* **2015**, *6* (May), 7129.

(8) Brynn Hibbert, D.; Thordarson, P. *Chem. Commun.* **2016**, *52* (87), 12792–12805.

(9) Dean, J. A.; Lange, N. A. *Lange's Handbook of Chemistry*, 15th ed.; McGraw-Hill: New York, 1999.

(10) http://www.hinsberg.net/kinetiscope/.
